# Light Paths and Dark Valleys: Topographic Complexity and Mammal Occupancy in a Semi‐Arid Mountain Landscape

**DOI:** 10.1002/ece3.73100

**Published:** 2026-02-13

**Authors:** Maya Beukes, Travis Perry, Dan Parker, Nokubonga Mgqatsa

**Affiliations:** ^1^ Audiovisual Biodiversity Research, Terrestrial Zoology Senckenberg Research Institute and Nature Museum Frankfurt am Main Germany; ^2^ Wildlife and Reserve Management Research Group, Department of Zoology and Entomology Rhodes University Makhanda South Africa; ^3^ Department of Biology Furman University Greenville South Carolina USA; ^4^ School of Biology and Environmental Sciences University of Mpumalanga Mbombela South Africa

**Keywords:** aspect, habitat selection, microclimate heterogeneity, mountain landscape ecology, ruggedness, slope, solar radiation, topographic gradients, topography and biodiversity

## Abstract

Topographically complex mountainous regions are widely recognized as important for biodiversity conservation due to their environmental heterogeneity, which can promote species turnover, niche differentiation, and the persistence of specialized taxa. Such landscapes are often associated with high biodiversity value and provide critical resources and connectivity for wildlife and human communities. In this study, we deployed 131 camera traps to assess the occupancy of 34 mammal species in relation to key topographic variables in a semi‐arid mountain catchment in South Africa. Multispecies occupancy models were used to evaluate the probability of habitat use concerning topographic complexity, characterized by features such as catchment aspect, slope, ruggedness, solar gain and landscape units. The results identified floodplains, valleys, low slopes, and areas with low ruggedness as biodiversity hotspots, offering critical resources like water and forage and supporting high species richness. Conversely, steep slopes, rugged terrains, and high solar gain areas, while supporting fewer species, served as critical refuges for specialized taxa such as leopard, klipspringer, caracal, and grey rhebok. While topographic features like ruggedness may have a limited impact at the community level, their importance becomes more pronounced at the species level. This study underscores the value of incorporating detailed topographic metrics into ecological research, particularly in mountainous landscapes where these features govern species distribution. Conservation strategies should integrate both community‐level and species‐specific monitoring approaches to safeguard the unique biodiversity and ecological dynamics of topographically complex mountain landscapes.

## Graphical abstract

Topographically complex mountainous regions are critical for biodiversity conservation, supporting high beta diversity, endemic species, and essential ecosystem services. Despite their importance, the fine‐scale effects of topographic metrics such as slope, ruggedness, and solar gain on mammal habitat use remain poorly understood. This study aimed to assess how topographic complexity shapes mammal occupancy patterns in a semi‐arid mountainous landscape by integrating detailed digital elevation model (DEM)‐derived metrics with multispecies occupancy models.

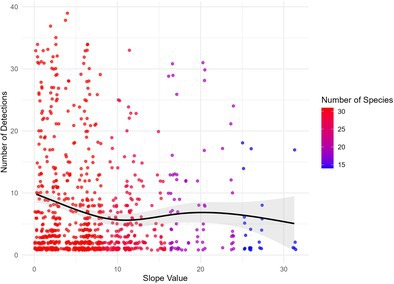

## 1 Introduction

Topographically complex regions, such as vast mountain ranges and deeply dissected plateaus, are globally recognized as biodiversity hotspots due to their ecological heterogeneity and the microhabitat diversity they support (Bouchet et al. 2015; Marchese 2015). Globally, topographic complexity (TC) is a significant driver of species richness, influencing taxonomic diversity at various spatial scales and across different groups (Badgley et al. 2017; Roell et al. 2021). TC also serves as a critical facilitator of ecological and evolutionary processes, providing refuge for species, promoting endemism, and enabling movement across fragmented landscapes (Yu et al. 2015).

The TC of landscapes arises from features such as wind, currents, bathymetry, and terrain, as well as structural elements, both natural and artificial (Buckland et al. 2005). These features influence how animals navigate their environments, making TC a central factor in shaping behavior and movement ecology (Tarolli 2014). Metrics such as slope, elevation, ruggedness, and solar gain add to TC, particularly in mountainous regions, where they constrain animal movement and habitat use (Jenness 2004; Sappington et al. 2007; Shepard et al. 2013; Tarolli 2014; Heit et al. 2023).

The interplay between TC and ecosystem dynamics often resists simple cause‐and‐effect explanations (Green et al. 2006). Movement occurs across one, two, or three dimensions depending on habitat structure and species‐specific adaptations, while steep or uneven slopes dictate routes, energy costs, and predation risk (Alderman and Hinsley 2007). Aspect and solar insolation further shape microclimates and vegetation patterns (Florinsky 2012; Gedir et al. 2020). Yet fine‐scale topographic variation remains underrepresented in ecological studies, limiting understanding of how these features drive species' behavior and distribution in mountainous and semi‐arid ecosystems (Sultaire et al. 2023). Neglecting such multidimensional interactions risks introducing bias into ecological models and highlights the need for comprehensive, fine‐scale approaches to monitoring and analysis (Montgomery et al. 2020).

Vegetation patterns are also intricately linked to TC, as elevation gradients and terrain features influence plant community composition and habitat quality (Dorner et al. 2002). In southern Africa, plant species richness is intricately linked to TC (Thuiller et al. 2006) and accounts for up to 75% of the variation in mammal species richness, emphasizing the strong ecological connection between vegetation, topography, and mammal distributions (Andrews and O'Brien 2000). Topographic complexity further drives fine‐scale ecological speciation and biodiversity gradients along elevation zones, creating niche opportunities for both generalist and specialist species (Fine 2015; Dilts et al. 2023).

Advances in geospatial technology, such as Geographic Information Systems (GIS) and Digital Elevation Models (DEMs), have transformed our ability to quantify terrain attributes and integrate them into ecological models (Lichtenberg et al. 2025). The DEMs derived from high‐resolution data sources such as LiDAR or the Shuttle Radar Topography Mission (SRTM) allow for precise calculations of slope, ruggedness, aspect, and solar gain (Reuter et al. 2007; Farr et al. 2007; Jarvis et al. 2008). These metrics enable researchers to explore the relationships between landscape structure and ecological processes at scales that were previously unattainable (Bolstad 2016; Wilson and Gallant 2000).

Despite the recognized importance of topographic heterogeneity in structuring ecological communities, the fine‐scale effects of terrain attributes such as slope, ruggedness, solar gain, and landscape units on mammal habitat use remain poorly understood, particularly in semi‐arid mountain systems. The primary objective of this study was to evaluate how multiple dimensions of topographic complexity influence mammal habitat use at both community and species levels within a semi‐arid mountainous catchment. Specifically, we assessed whether resource‐rich and more accessible landscape units (e.g., floodplains and valleys) are associated with higher community occupancy, while steeper, more rugged, and thermally exposed areas support fewer species overall but may function as important habitats for specialized taxa. By focusing on fine‐scale topographic features rather than broad elevation gradients, this study aims to provide a more nuanced understanding of how terrain heterogeneity shapes mammal distributions in complex landscapes (McCain 2005; Roell et al. 2021).

## 2 Materials and Methods

### 2.1 Study Area

**FIGURE 1:**
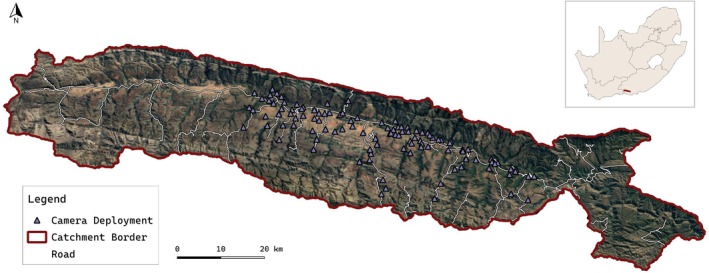
Location of the Baviaanskloof catchment within South Africa, with camera trap deployments and secondary roads shown, overlaid on a satellite image.

The study site is the 1234 km^2^ Baviaanskloof catchment, a semi‐arid, mountainous watershed located in South Africa's Eastern Cape Province (Figure 1). Situated within the Cape Fold Mountain Belt, the catchment is dominated by steep mountains underlain by quartzitic sandstone geology (Boshoff 2005). Historical faulting and uplifts have shaped the landscape, forming a central valley that runs parallel between the Baviaans and Kouga mountain ranges (Holmes 2012). The central valley floodplain varies in width, spanning up to one kilometer but narrowing to just 100 m in places. Steep, deeply incised tributary valleys feed perpendicularly into the central valley, with many terminating in alluvial fans along the floodplain margin (Glenday 2015). The valleys, floodplains, and alluvial fans account for only 5% of the catchment area, and feature gentle slopes of 0.6%. In contrast, the remainder of the catchment has an average slope of 38% (Powell 2009).

Soils across the catchment vary significantly. The cliffs, hillslopes, and plateaus are characterized by thin, rocky soils (0–100 cm depth), consisting mainly of loamy sands with a high rock content of 30%–40% (Glenday 2015). Vegetation distribution aligns closely with the topography, with fynbos, composed of woody shrubs, grasses, and herbs, dominating the plateaus and upper slopes; subtropical thicket, featuring large succulents and woody shrubs, found on cliffs and lower slopes; riparian forests occupying narrow tributary gorges and stretches of the main valley; and savanna covers the expansive floodplains (Euston‐Brown 2006).

Rainfall is highly variable, averaging 270 mm annually but ranging from 100 to 500 mm with no clear seasonal pattern (del Río‐Mena et al. 2021). Temperatures fluctuate from over 40°C in summer to below 0°C in winter (Van Luijk et al. 2013). Combined with diverse topography and soils, this climatic variability creates a unique ecological gradient that supports varied vegetation and land uses, with fertile valley bottoms largely devoted to agriculture (Ndeketeya 2012).

### 2.2 Camera Trap Survey Design

Between January 2020 and April 2022, 131 unique camera traps were deployed across four survey sessions, with each session lasting approximately 180 days to account for species with variable encounter rates (Tobler and Powell 2013; Beukes et al. 2025b). The camera trap array comprised Cuddeback Color X‐Change cameras (*n* = 119), Bushnell Trophy Cam cameras (*n* = 9), and Ltl‐Acorn cameras (*n* = 3). Camera placement followed a stratified random design, incorporating topographical variables alongside ecological and land‐use factors. Stratification was based on vegetation types (thicket, fynbos, forest, savanna), agricultural activities (e.g., cultivated lands, grazing lands, and resting farmland), degradation levels (transformed, severely degraded, moderately degraded, intact vegetation), and key topographic features (e.g., slope, aspect, elevation, and ruggedness). Stratified points were generated using Geospatial Modeling Environment 0.7.2 RC2 (Spatial Ecology LLC 2012).

To maximize the detection of both generalist and elusive species, cameras were placed on animal trails, roads, and off‐track locations and operated continuously for 24 h a day. Cameras were configured to capture a single photograph per trigger event, with a 30‐s delay between consecutive triggers (Sollmann et al. 2013; Tobler and Powell 2013). They were checked every 30–45 days to download images, replace batteries, verify functionality, and remove obstructing vegetation (Kok 2016). Sampling effort was quantified in camera trap nights, calculated as the total functional nights per camera (Colyn et al. 2018). Mammals weighing over 1 kg were identified using a field guide (Skinner and Chimimba 2005). Consecutive captures of the same species within a 30‐min time frame were treated as a single event to minimize autocorrelation (Tobler et al. 2008; Tambling et al. 2015). The image database was managed using Timelapse software (Greenberg et al. 2019).

### 2.3 Topographic Classification

We derived a 30 m Digital Elevation Model (DEM) from processed SRTM v4.1 data (Reuter et al. 2007; Jarvis et al. 2008; Bolstad 2016) and used GRASS GIS (GRASS Development Team 2024) to calculate four key terrain variables: slope (°), aspect (°), ruggedness (Terrain Ruggedness Index), and solar gain (Wh/m^2^) (Appendix S1). To capture the seasonal variation of solar gain, estimates were derived for 12 cloud‐free days (one per month) across 12 months, then averaged to produce an annual mean. For each camera deployment, all topographic variables were extracted within a 100 m buffer and averaged to provide spatially robust estimates of the surrounding conditions. Although seven geomorphological landscape units were initially identified (Appendix S2), cliff habitats were excluded from subsequent analyses due to inaccessibility and the absence of camera trap deployments, resulting in six landscape units being retained for analysis.

Each topographic variable was subsequently classified into ecologically meaningful categories to facilitate analysis and interpretation (Table 1). Classification thresholds were defined using natural breaks (Jenks optimization) applied to the empirical distribution of each variable across the study area, ensuring that category boundaries reflected distinct gradients in terrain structure and energy availability rather than arbitrary cut‐offs (North 2009). Resulting classes (e.g., low, medium, high) therefore correspond to specific value ranges for each variable, with full threshold values provided in Table 1 and detailed in Appendix S1. The same classification scheme was applied consistently across all analyses, figures, and supplementary tables.

**TABLE 1:** Summary of topographic variables derived from the DEM and their classification.

Variable	Description	Calculation method (GRASS GIS)	Units/Range	Classification	Ecological rationale/references
Solar gain	Measure of solar energy received at the surface, influencing microclimates and vegetation productivity	*r.sun*; incorporates slope, aspect, and terrain shadowing; averaged across 12 monthly values	Wh/m^2^	4 classes: low, medium‐low, medium‐high, high	Influences microclimate and vegetation productivity; expected nonlinear effects on habitat use (Fu and Rich 2002; Neteler and Mitasova 2008; Sears and Angilletta Jr 2015)
Slope	Rate of elevation change affects hydrology, erosion, and movement cost	*r.slope.aspect:* moving‐window gradient of DEM cells	Degrees (0°–32°)	4 classes: low, medium, high, very high	Increasing slope increases movement cost and limits forage, reducing community occupancy but favouring slope‐adapted species (Wilson and Gallant 2000; Alderman and Hinsley 2007; Yang et al. 2020)
Aspect	Cardinal direction of slope; shapes microclimate and vegetation patterns	*r.slope.aspect*: cell orientation 0°–360°	Degrees (°)	4 categories: N, E, S, W	Drives thermal and moisture gradients affecting species‐specific habitat use (Bennie et al. 2008; Dobrowski 2011; Lemenkova 2022)
Ruggedness	Terrain roughness: higher values indicate complex, uneven terrain	*r.terrain* (TRI): elevation differences between cells	TRI index (1–130)	3 classes: low, medium, high	Provides refuge and structural complexity; expected to favour specialists while reducing generalist use (Jenness 2004; Bouchet et al. 2015; Smith et al. 2019)
Landscape units	Distinct landform types combine vegetation, hydrology, and topography	Delineated following Glenday (2015)	Categorical	6 types: hillslopes, plateaus, cliffs, canyon floors, floodplains, alluvial fans	Integrate hydrology, vegetation, and topography, shaping resource availability and habitat suitability (Ludwig et al. 2005; Glenday 2015; Snider et al. 2024)

### 2.4 Data Analysis

Analyses were conducted in R 4.3.0 (R Core Team 2022). Species‐by‐site detection histories were compiled in binary format using the *camtrapR* package (Niedballa et al. 2015), with five‐day sampling occasions to balance temporal independence and detectability (Burton et al. 2015). Temporal independence of capture events was addressed by defining independent detection events as records of the same species at the same site separated by ≥ 30 min, thereby reducing temporal autocorrelation associated with repeated capture events of the same individuals (Tobler et al. 2008; Tambling et al. 2015). This temporal filtering approach is commonly used in camera‐trap studies and is appropriate for occupancy modeling, where independence is assumed at the level of sampling occasions rather than individual capture events (MacKenzie et al. 2002). All recorded species were included in species richness estimates, whereas only species with more than five capture events were included in occupancy models to ensure model convergence (MacKenzie et al. 2002). Camera effort (trap‐nights) was included as a covariate to account for uneven sampling among deployments.

We applied Bayesian hierarchical multispecies occupancy models (MSOM) in a single‐season framework (Dorazio and Royle 2005; Kéry and Royle 2021) to jointly estimate species occupancy (*ψ*) and detection probabilities (*p*) while correcting for imperfect detection (MacKenzie et al. 2002). Detection probability was estimated as part of the occupancy framework to account for imperfect detection, but results for *p* are not reported because the study focuses on patterns in occupancy (*ψ*) across topographic gradients. Each camera site (*i*) was treated as an independent sampling unit, with > 1 km spacing between cameras supporting spatial independence.

Following the standard detection–occupancy decomposition (Dorazio and Royle 2005; Rota et al. 2016), occupancy state for species *s* at site *i* was modeled as:

$$z_{\text{is}}∼\text{Bernoulli}\left(\psi_{\text{is}}\right)$$

where *z*_*is*_ = 1 if species *s* occurs at site *i*, and 0 otherwise.

Observed capture events were conditional on true occupancy:

$$y_{\mathrm{ijs}}∼\text{Bernoulli}\left(z_{\text{is}}\cdot p_{\mathrm{ijs}}\right)$$

where *y*_*ijs*_ denotes detection/non‐detection of species *s* at site *i* during occasion *j*.

In this study, detection probability (*p*) is interpreted biologically as the probability that a species present at a site is recorded by a camera trap during a sampling occasion, reflecting a combination of animal movement, activity patterns, habitat structure, and camera performance rather than true abundance. Detection probability was modeled as a function of topographic context, including landscape unit and aspect as categorical covariates and slope, ruggedness, and solar gain as continuous covariates, allowing detectability to vary across terrain types due to differences in visibility, animal movement, and camera performance.

Occupancy probabilities were modeled as a function of TC, represented by categorical classes (landscape unit, aspect) and continuous covariates (slope, ruggedness, solar gain, all *z*‐standardized).

Formally, the logit‐linear predictor for occupancy was:

$$\text{logit}\left(\psi_{\text{is}}\right)=\beta_{0s}+\beta_{s,\mathrm{TC}}\left({\mathrm{TC}}_{i}\right)+\beta_{s,k}X_{\mathrm{ik}}$$

where *β*_*0s*_ is the species‐specific intercept, *β*_*s*,TC_ is the effect of topographic class at site *i*, and *β*_*s,k*_ is the species‐specific slopes for continuous covariates *X*_*ik*_.

Detection probability was modeled as:

$$\text{logit}\left(p_{\text{is}}\right)=\alpha_{0s}+\alpha_{s,\mathrm{TC}}\left({\mathrm{TC}}_{i}\right)$$

allowing detectability to vary across topographic contexts.

We used weakly informative priors: *β*_*0s*_, *β*_*s*,TC_, *β*_*s,k*_ ∼ *N*(0,0.001) for occupancy intercepts and covariate effects, and *P* ∼ Beta (1,1) as an uninformative prior on detection probabilities.

Models were fit in JAGS using *rjags* and *jagsUI* (Plummer 2003), with three MCMC chains of 100,000 iterations, a burn‐in of 50,000, and thinning of 10, yielding 15,000 posterior samples per parameter. Convergence was assessed with trace plots and Gelman–Rubin statistics (all ≤ 1.1) (Gelman et al. 2014). Posterior means and 95% Bayesian credible intervals (BCIs) were reported. Covariate effects were considered strongly supported if the 95% BCI did not overlap zero and moderately supported if the 75% BCI did not overlap zero. Posterior means are reported in the Results, while full posterior summaries, including BCIs, are provided in the Supporting Information. Occupancy estimates are interpreted as habitat use probabilities, not true occupancy, since some species' ranges may extend beyond the sampling units and could violate closure assumptions (Efford and Dawson 2012).

In addition to model‐based occupancy estimates, we report descriptive metrics derived directly from camera‐trap data to summarize patterns of mammal occurrence across topographic categories. These include (i) independent capture events, defined as temporally filtered records (≥ 30 min separation) of the same species at the same site; (ii) proportion of capture events, calculated as the proportion of total independent events recorded within each category; and (iii) species richness, defined as the total number of species recorded per category. These descriptive metrics are intended to provide contextual information on sampling outcomes and observed community patterns and were not subjected to formal statistical modeling.

## 3 Results

### 3.1 Survey Design

Camera deployments were distributed across all major landscape units and topographic gradients, ensuring comprehensive coverage of environmental conditions. Deployment distributions by topographic category are provided in Appendix S3. Across 21,020 camera‐trap nights, a total of 6099 independent capture events were recorded, representing 34 mammal species. Species identities, taxonomic groupings, and sampling frequencies (number of capture events and sites) are detailed in Appendix S4.

### 3.2 Community‐Level Responses to Topography

Patterns of mammal occurrence varied across topographic gradients, as reflected by differences in independent capture events, species richness, and model‐based occupancy probabilities (*ψ*) (Appendices S3 and S5). Aspect‐shaped communities with east‐facing slopes yielded the highest capture events (36%), and south‐facing sites represented the highest occupancy (*ψ* = 0.33, BCI = 0.13–0.54), while west‐facing sites were the least productive (9% capture events; *ψ* = 0.24, BCI = −0.00–0.49). Ruggedness showed a similar gradient, with low‐ruggedness sites hosting most detections (61%) and species (32), compared to high‐ruggedness sites with low capture events (7%) and occupancy (*ψ* = 0.25, BCI = 0.07–0.44).

Solar gain exhibited a nonlinear effect, with intermediate medium‐high levels maximizing richness (29 species) and community occupancy (*ψ* = 0.34, BCI = 0.13–0.55), whereas high solar gain areas reduced capture events (9%) and occupancy (*ψ* = 0.24, BCI = 0.01–0.46). Similarly, slope influenced distributions, with low‐slope sites dominating capture events (66%) and species richness (30), while high slopes supported fewer capture events (11%) but the highest occupancy estimate (*ψ* = 0.34, BCI = 0.12–0.59).

Landscape units reflected solar gain, with valleys and floodplains supporting the highest proportion of capture events (23% and 28%) and relatively diverse assemblages (28 and 23 species), while high plateaus had the lowest capture events (7%) and fewer species (22). Occupancy estimates were broadly consistent across units, with floodplains slightly higher (*ψ* = 0.31, BCI = 0.08–0.54) than plateaus (*ψ* = 0.26, BCI = 0.05–0.48). However, at the species level, pronounced differences across topographic features emerge (Appendices S6 and S5).

### 3.3 Species‐Level Occupancy Responses

#### 3.3.1 Occupancy by Aspect

**FIGURE 2:**
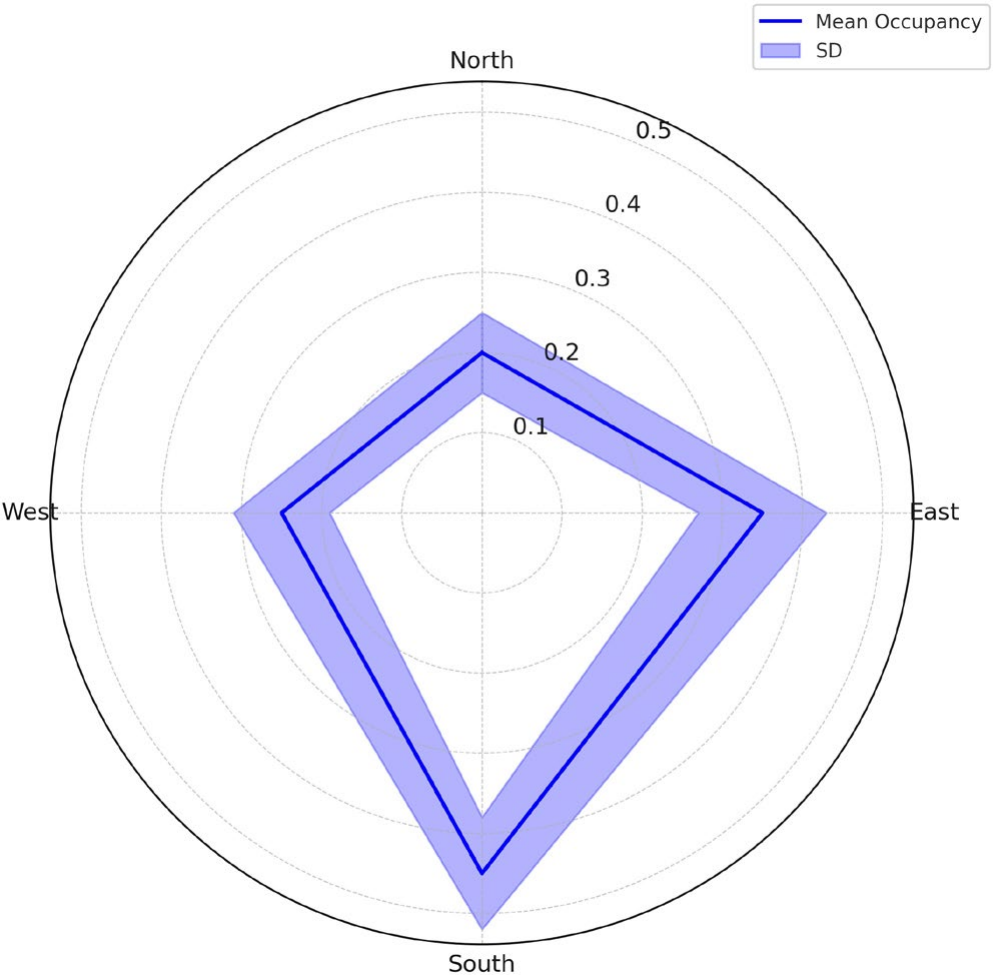
Community occupancy rates in relation to aspect, aggregated across species for each cardinal aspect category (North, East, South, and West). The radial axis corresponds to the mean occupancy rates, ranging from 0 at the center to the maximum observed occupancy value (e.g., 1) at the plot's outer edge, with shaded bands representing 95% Bayesian credible intervals.

North‐facing slopes supported high occupancy probabilities for species like bontebok ( *Damaliscus pygargus* ; *ψ* = 0.88), Cape grysbok (*Raphicerus melanotis; ψ* = 0.87), and scrub hare ( *Lepus saxatilis* ; *ψ* = 0.80), reflecting the suitability of these areas for grazers and small herbivores. Mountain reedbuck ( *Redunca fulvorufula* ; *ψ* = 0.71) also demonstrated a preference for North‐facing aspect, while leopard ( *Panthera pardus* ; *ψ* = 0.49) and bat‐eared fox ( *Otocyon megalotis* ; *ψ* = 0.48) preferred these terrains to a lesser extent (Figure 2).

South‐facing slopes exhibited the highest occupancy probabilities among aspects, particularly for fossorial and nocturnal species such as aardvark ( *Orycteropus afer* ; *ψ* = 0.95), Cape porcupine ( *Hystrix africaeaustralis* ; *ψ* = 0.93), caracal (*Caracal caracal; ψ* = 0.92), and klipspringer ( *Oreotragus oreotragus* ; *ψ* = 0.86), highlighting the importance of these coolers, shaded habitats for both predators and small herbivores. Other notable occupants include bushpig ( *Potamochoerus larvatus* ; *ψ* = 0.61), aardwolf ( *Proteles cristata* ; *ψ* = 0.59), gemsbok ( *Oryx gazella* ; *ψ* = 0.47), and chacma baboon ( *Papio ursinus* ; *ψ* = 0.47).

East‐facing slopes provided moderate occupancy probabilities for species such as eland ( *Taurotragus oryx* ; *ψ* = 0.92), Smith's red rock hare ( *Pronolagus rupestris* ; *ψ* = 0.66), and vervet monkey ( *Chlorocebus pygerythrus* ; *ψ* = 0.49), possibly suggesting their preference for the morning sunlight and associated vegetation productivity. Cape grey mongoose (*Herpestes pulverulentus*; *ψ* = 0.47) and black‐backed jackal (*Canis mesomelus*; *ψ* = 0.43) also inhabited these areas, alongside grazers like springbok ( *Antidorcas marsupialis* ; *ψ* = 0.40), impala ( *Aepyceros melampus* ; *ψ* = 0.34), and bushbuck (*Tragelaphus sylvaticus*; *ψ* = 0.34).

West‐facing slopes supported fewer species with high occupancy probabilities; however, grey rhebok (*Pelea capreolus; ψ* = 0.56) and African wild cat (*Felis lybica cafra; ψ* = 0.49) showed relatively high habitat‐use probabilities in these aspects.

#### 3.3.2 Occupancy by Ruggedness

**FIGURE 3:**
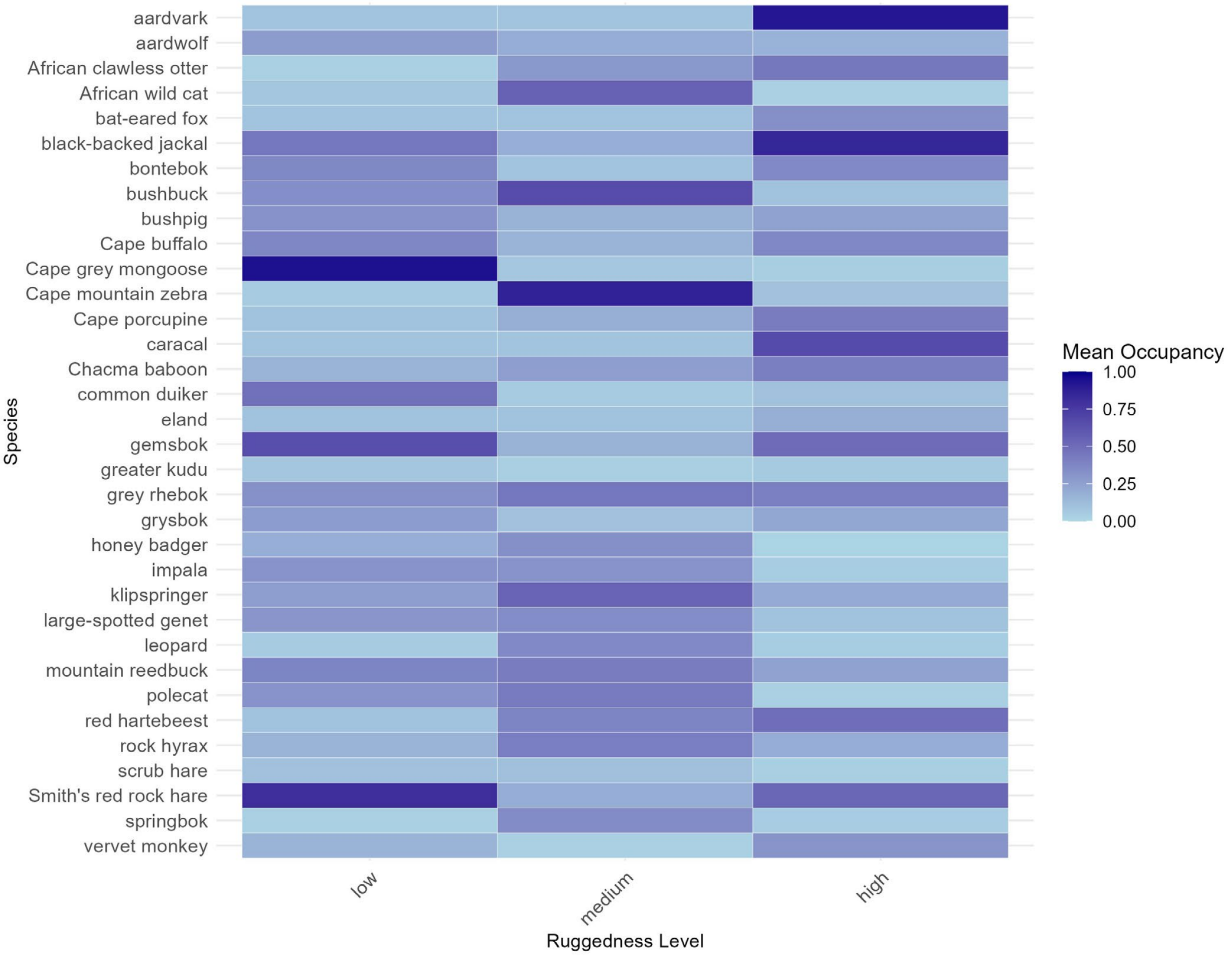
Heatmap depicting variation in species occupancy across ruggedness levels (low, medium, and high). The color gradient represents mean occupancy, with lighter shades of blue indicating lower occupancy and darker shades representing higher occupancy.

The influence of ruggedness on species occupancy varied, with many species showing no significant effects, indicating that ruggedness had a weaker impact on occupancy estimates for some species (Figure 3). Non‐significant results were observed for species such as bat‐eared fox, bontebok, bushbuck, eland, impala, leopard, Cape Mountain zebra, Cape porcupine, and scrub hare. However, for certain species, ruggedness played a more notable role in shaping habitat use. For instance, high‐ruggedness terrain was favoured by aardvark (*ψ* = 0.92), which had the highest occupancy probability in these areas. Larger herbivores like Cape buffalo ( *Syncerus caffer* ; *ψ* = 0.67) and red hartebeest (*Alcelaphus buselaphus; ψ* = 0.50) also utilized high‐ruggedness terrains. Species such as Cape grysbok (*ψ* = 0.45), black‐backed jackal (*ψ* = 0.44), chacma baboon (*ψ* = 0.41), mountain reedbuck (*ψ* = 0.38), vervet monkey (*ψ* = 0.31), and large spotted genet (*Genetta tigrina; ψ* = 0.30) showed moderate occupancy in high‐ruggedness.

Medium‐ruggedness terrains were hotspots for small herbivores and carnivores, with grey rhebok (*ψ* = 0.94) and klipspringer (*ψ* = 0.85) showing the highest occupancy probabilities, indicative of their adaptability to rocky terrains. Common duiker ( *Sylvicapra grimmia* ; *ψ* = 0.66), African wild cat (*ψ* = 0.55), polecat ( *Ictonyx striatus* ; *ψ* = 0.45), rock hyrax ( *Procavia capensis* ; *ψ* = 0.42), and springbok (*ψ* = 0.35) also occupied areas of medium‐ruggedness. Whereas low‐ruggedness terrains were preferred by species requiring open habitats with easier mobility, such as African clawless otter ( *Aonyx capensis* ; *ψ* = 0.87) and Smith's red rock hare (*ψ* = 0.81). Species like caracal (*ψ* = 0.67), gemsbok (*ψ* = 0.67), greater kudu ( *Tragelaphus strepsiceros* ; *ψ* = 0.54), Cape grey mongoose (*ψ* = 0.50), and honey badger ( *Mellivora capensis* ; *ψ* = 0.41) also showed moderate occupancy in low‐ruggedness.

#### 3.3.3 Occupancy by Slope

**FIGURE 4:**
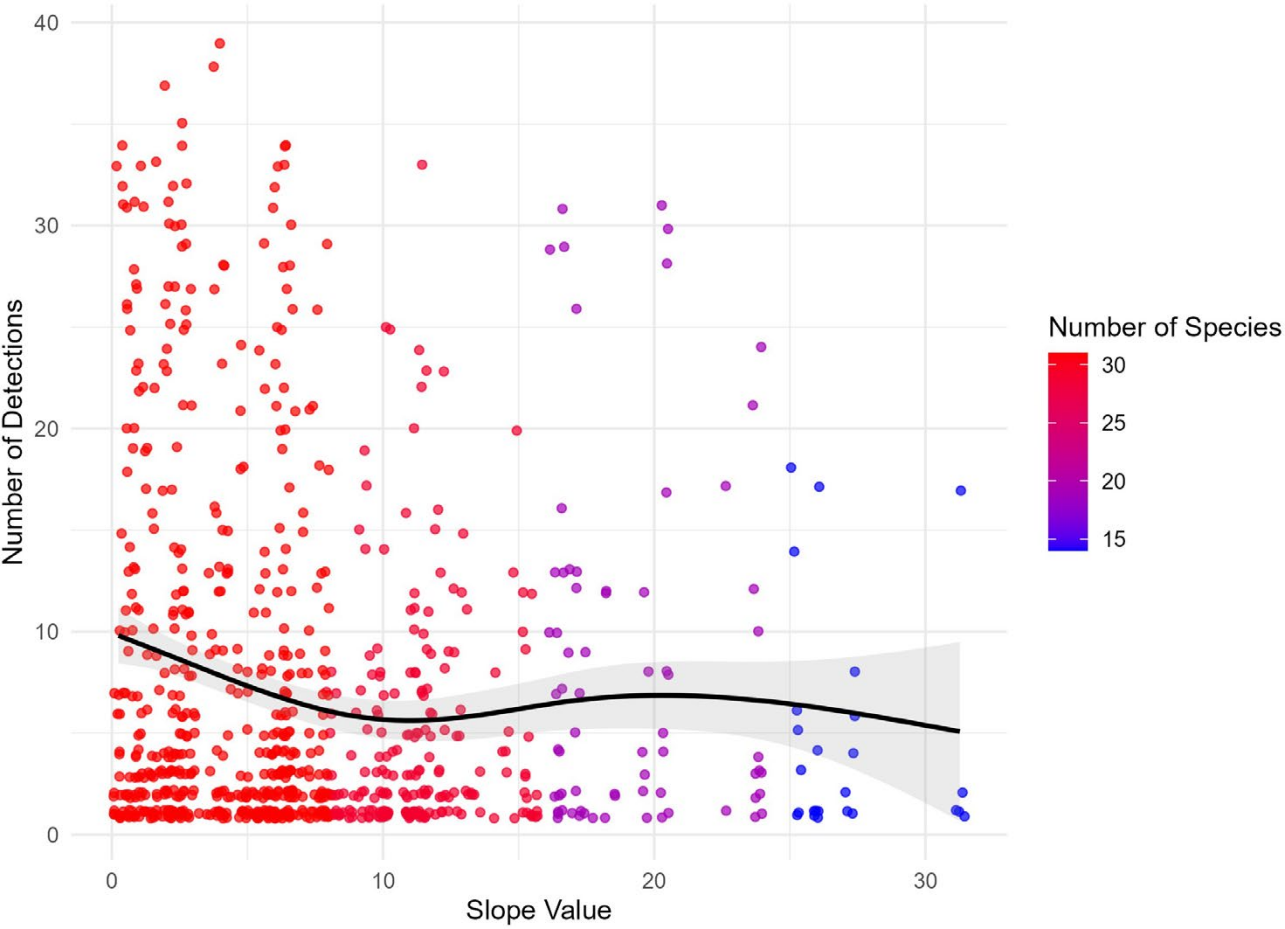
The interaction between terrain steepness, detections (independent capture events), and species richness across camera deployments. Points representing species capture events per deployment are color‐coded by the number of unique species per slope category: Low (0°–8°), medium (9°–16°), high (17°–24°), and very high (> 24°). Slope values range from 0° (flat) to 32° (steep). A GAM trendline shows the overall detection trend, with a shaded ribbon indicating confidence intervals.

The analysis of species occupancy and richness in relation to slope revealed varying habitat preferences tied to topographic inclination, with species richness and capture events declining with increased slope (Figure 4). Species like Cape grysbok (*ψ* = 0.91), mountain reedbuck (*ψ* = 0.77), bontebok (*ψ* = 0.71), greater kudu (*ψ* = 0.60), bat‐eared fox (*ψ* = 0.46), and grey rhebok (*ψ* = 0.48) exhibited high occupancy in areas with low slope gradients, suggesting preference for flat or rolling landscapes. In medium‐slope areas, species like eland (*ψ* = 0.91), African clawless otter (*ψ* = 0.73), gemsbok (*ψ* = 0.56), Smith's red rock hare (*ψ* = 0.50), Cape grey mongoose (*ψ* = 0.43), impala (*ψ* = 0.35), and large spotted genet (*ψ* = 0.35) exhibited the highest occupancy. Cape Mountain zebra (*ψ* = 0.30) and Cape buffalo (*ψ* = 0.21) appeared to utilize medium slopes more frequently, despite their more generalized slope preferences.

Klipspringer (*ψ* = 0.91), caracal (*ψ* = 0.91), and Cape porcupine (*ψ* = 0.90) dominate steep (high slope) terrains. Black‐backed jackal (*ψ* = 0.67), rock hyrax (*ψ* = 0.53), common duiker (*ψ* = 0.56), and chacma baboon (*ψ* = 0.28) also exhibited moderate occupancy in high slope terrains. Species such as red hartebeest (*ψ* = 0.54), leopard (*ψ* = 0.51), and polecat (*ψ* = 0.48) occupied very steep slopes. Aardwolf (*ψ* = 0.26) also exhibited low but measurable occupancy in very steep terrain. Species such as bushbuck, bushpig, honey badger, scrub hare, springbok, vervet monkey, and African wild cat showed no significant slope preferences.

#### 3.3.4 Occupancy by Solar Gain

**FIGURE 5:**
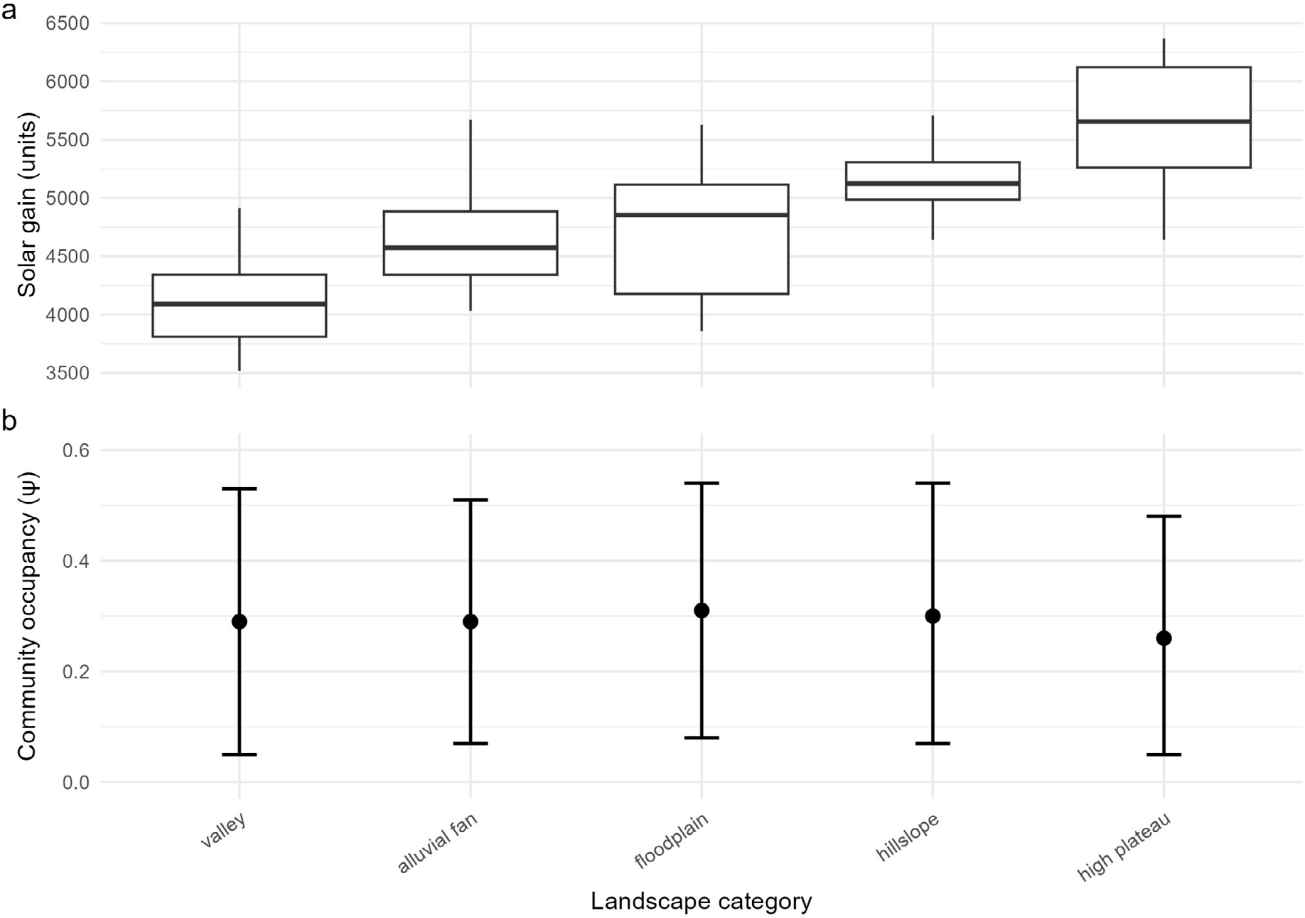
Distributions of (a) solar gain and (b) community‐level occupancy (*ψ*) across landscape categories. Solar gain is shown as boxplots (median, interquartile range; whiskers = 1.5 × IQR). Community occupancy points represent posterior means with 95% Bayesian credible intervals.

The analysis of species occupancy relative to solar gain reveals distinct preferences across different levels of solar exposure (Figure 5). At low solar gain, species such as bontebok (*ψ* = 0.89), scrub hare (*ψ* = 0.84), Cape grysbok (*ψ* = 0.72), gemsbok (*ψ* = 0.67), grey rhebok (*ψ* = 0.61), and Smith's red rock hare (*ψ* = 0.58) showed the highest mean occupancy, indicating that these species preferred areas with limited solar radiation. At medium‐low solar gain, species such as eland (*ψ* = 0.94), mountain reedbuck (*ψ* = 0.84), vervet monkey (*ψ* = 0.76), chacma baboon (*ψ* = 0.60), African wild cat (*ψ* = 0.54), and rock hyrax (*ψ* = 0.46) demonstrated higher occupancy. For medium‐high solar gain, species like klipspringer (*ψ* = 0.96), caracal (*ψ* = 0.94), aardvark (*ψ* = 0.79), and red hartebeest (*ψ* = 0.62) showed the strongest occupancy. In high solar gain areas, common duiker (*ψ* = 0.67), leopard (*ψ* = 0.67), and bat‐eared fox (*ψ* = 0.52) exhibited significant occupancy. Whereas, aardwolf, Cape buffalo, bushbuck, honey badger, greater kudu, large spotted genet, Cape Mountain zebra ( *Equus zebra* ), and springbok showed no significant association with any specific solar gain level.

#### 3.3.5 Occupancy by Landscape Unit

Mammal occupancy probabilities revealed distinct habitat preferences across landscape units, closely paralleling patterns observed for solar gain (Figure 5), underscoring the role of landscape units in supporting diverse species (Appendices S6 and S5).

Alluvial fans emerged as critical habitats for species like rock hyrax (*ψ* = 0.91), bushbuck (*ψ =* 0.75), and leopard (*ψ =* 0.71), which thrived in the structural complexity and resource availability of these areas. Smith's red rock hare (*ψ =* 0.59) and polecat (*ψ =* 0.47), to a lesser extent, also utilized this terrain.

Hillslopes hosted the highest occupancy probabilities for leopard (*ψ =* 0.92) and African clawless otter (*ψ =* 0.89), indicative of the specialized niches found in these steeper terrains. Bushpig (*ψ =* 0.66), gemsbok (*ψ =* 0.59), aardwolf (*ψ =* 0.57), Cape buffalo (*ψ =* 0.54), and regionally iconic species like greater kudu (*ψ =* 0.54), chacma baboon (*ψ =* 0.51), and Cape Mountain zebra (*ψ =* 0.48) highlight the ecological value of hillslopes for diverse trophic levels.

Floodplains stood out for their high occupancy probabilities for aardvark (*ψ =* 0.92) and bat‐eared fox (*ψ =* 0.92), emphasizing their suitability for fossorial and insectivorous species. Cape grysbok (*ψ =* 0.79) and springbok (*ψ =* 0.78) illustrated the importance of these areas for small to medium herbivores, while impala (*ψ =* 0.62) and vervet monkey (*ψ =* 0.56) highlight the floodplain's role in supporting grazers.

High plateaus provided critical habitats for species like bushbuck (*ψ =* 0.93), common duiker (*ψ =* 0.90), and eland (*ψ =* 0.73), which showed strong preferences for these elevated terrains. Bontebok (*ψ =* 0.54) and Cape porcupine (*ψ =* 0.39) also utilized these areas, reflecting the plateau's role in supporting both browsers and grazers.

Valleys were characterized by high occupancy probabilities for honey badger (*ψ =* 0.84) and klipspringer (*ψ =* 0.79), species that benefit from the valley's shelter and resource availability. Scrub hare (*ψ =* 0.67), black‐backed jackal (*ψ =* 0.53), and mountain reedbuck (*ψ =* 0.45) also thrived in this terrain, alongside Cape grey mongoose (*ψ =* 0.40) and grey rhebok (*ψ =* 0.40).

Landscape units did not significantly affect caracal, large spotted genet, and red hartebeest. These findings illustrate how mammal communities adapt to the unique environmental features of each landscape unit, with specific species demonstrating strong preferences that reflect their ecological requirements and behavioral adaptations.

## 4 Discussion

Mountainous regions are widely recognized as biodiversity hotspots because strong topographic gradients create pronounced environmental heterogeneity, which promotes species turnover and the coexistence of taxa with contrasting habitat requirements (Li et al. 2018; Roell et al. 2021; Theobald et al. 2024). In this study, we show that topographic complexity (TC) within a semi‐arid mountainous catchment plays a decisive role in structuring mammal habitat use, with distinct occupancy patterns emerging across slope, ruggedness, aspect, and solar gain gradients. Although beta diversity was not explicitly quantified, the presence of 34 mammal species exhibiting strongly divergent topographic associations is consistent with high spatial turnover in species composition across the landscape, a pattern commonly reported in heterogeneous mountain systems (McCain 2005; Rahbek et al. 2019).

While many ecological studies underrepresent the role of fine‐scale topography (Snider et al. 2024; Sultaire et al. 2023), our results demonstrate that broad community‐level averages can obscure pronounced species‐specific habitat use. For example, greater kudu primarily occupied gentle slopes with high solar gain, whereas klipspringer were restricted to steep, rugged terrain. Similar contrasts have been documented in savanna and montane systems, where browsers, grazers, and carnivores partition habitat along gradients of slope, aspect, and cover availability (Lagendijk et al. 2015; Davies et al. 2016). These patterns highlight the importance of integrating both community‐level and species‐specific perspectives when assessing biodiversity and informing conservation planning. More detailed descriptions of species‐specific topographic niches are provided in Appendix S7.

### 4.1 Aspect

Aspect‐driven microclimates play a key role in shaping mammal distributions, often outweighing regional temperature patterns (Dobrowski 2011). In our study, south‐facing slopes, which are cooler and less exposed, supported species such as porcupine, caracal, and klipspringer. This aligns with observations in Mediterranean and Andean systems, where shaded slopes function as refuges for thermally sensitive taxa (Bennie et al. 2008; Lawson et al. 2014).

In contrast, north‐facing slopes supported a greater number of species despite lower average community occupancy probabilities, indicating that these areas are used by a wider range of species, each occurring at relatively lower probabilities. This pattern likely reflects temporally partitioned site use, whereby nocturnal or wide‐ranging species (e.g., leopard and aardvark) use north‐facing slopes intermittently. Such temporally staggered use increases the number of species recorded within this aspect category without implying sustained or frequent use by all species. Similar nocturnal exploitation of hot slopes has been recorded in arid rangelands (Florinsky 2012; Gedir et al. 2020). East‐facing slopes, which warm early, were favored by species such as grey rhebok and vervet monkeys, consistent with studies showing herbivores concentrate on these slopes for morning foraging (Lagendijk et al. 2015). West‐facing slopes supported fewer capture events overall, echoing findings from semi‐arid savannas where late‐day heat and reduced vegetation limit habitat use. These results emphasize the role of aspect in structuring both thermal refuges (south‐facing) and resource opportunities (east‐facing).

### 4.2 Slope

Slope influences forage availability, accessibility, and predation risk, shaping mammal distributions in diverse ecosystems (Badano et al. 2005; Yang et al. 2020). In the Baviaanskloof, vegetation varies along altitudinal gradients, with slopes often providing richer forage resources than valley bottoms (Euston‐Brown 2006; Petz et al. 2014). Gentle slopes, known for their ecological stability and resource abundance, supported the highest mammal capture events and species richness. Species such as Cape grysbok, bontebok, and greater kudu were frequently associated with these areas, a pattern corroborated by studies showing that lower elevations generally sustain higher richness and relative abundance than higher elevations (Lagendijk et al. 2015; Snider et al. 2024). These species benefit from the gentle terrain through ease of movement and reliable forage, reducing the energetic costs of traversing steeper gradients. Moderately steep slopes, in contrast, supported species such as grey rhebok, impala, and large spotted genet, which appear to take advantage of the structural heterogeneity these areas provide, balancing access to forage with cover and shelter.

Steeper slopes, while exhibiting lower average occupancy probabilities at the community level, supported species such as klipspringer, caracal, and black‐backed jackal. Very steep slopes serve as strategic vantage points or refuges for species like leopards and red hartebeest, mirroring studies from East African highlands and the Drakensberg, where steep escarpments provide both refuge and seasonal forage (Rowe‐Rowe 1983; Davies et al. 2016). These gradients facilitate resource partitioning, with nocturnal species like aardvark and porcupine exploiting steep areas at night, reducing competition and thermal stress. Such diel strategies are consistent with findings from other arid systems (Sears and Angilletta Jr 2015).

### 4.3 Solar Gain

Solar gain shapes local thermal environments, influencing how mammals balance forage access with heat stress (Bennie et al. 2008; Sears and Angilletta Jr 2015). Microclimates result from the interaction of physical factors such as ambient temperature and solar radiation, which define the thermal conditions at ground level (Bennie et al. 2008). Slope and aspect are critical in determining the amount of solar radiation received, directly influencing local temperature variations (Badano et al. 2005). This spatial variation in thermal heterogeneity dictates how and when organisms interact with landscape patches, influenced by their thermal preferences and physiological limits (Sears and Angilletta Jr 2015). Recognizing these dynamics is crucial for pinpointing localized thermal refuges offering relief from temperature extremes and broader‐scale thermal refugia that buffer against climatic changes over time (Keppel and Wardell‐Johnson 2012).

Areas with lower solar gain, such as shaded or cooler slopes, often serve as vital habitats by mitigating extreme temperatures and supporting thermally sensitive species (Morelli et al. 2016). Similarly, our results show that areas of low to medium‐high solar gain supported higher species richness and higher average occupancy probabilities across species. Klipspringer and caracal were most common in medium‐high solar gain sites, while impala and grysbok were associated with medium‐low levels. Similar patterns have been observed in Mediterranean shrublands and Australian woodlands, where moderate insolation promotes both vegetation productivity and thermal refuge (Keppel and Wardell‐Johnson 2012; Morelli et al. 2016).

High solar gain areas, by contrast, supported fewer species overall, consistent with studies from desert systems where exposure and heat stress limit diurnal use (Florinsky 2012). Nonetheless, some species, such as leopard and aardvark, occupied these areas, likely shifting activity to cooler night hours, a strategy widely reported across arid regions (Montgomery et al. 2020). Other species, such as aardwolf and buffalo, showed no clear solar gain preference, indicating broad tolerance or stronger influences from forage and water availability.

### 4.4 Ruggedness

Ruggedness provides structural complexity that shapes accessibility, refuge, and predator–prey interactions (Riley et al. 1999; Bouchet et al. 2015). At the community level, average occupancy probabilities declined with increasing ruggedness, but species‐level results showed contrasting patterns. Generalist grazers such as kudu and impala were concentrated in smoother terrain, whereas cliff‐dwelling specialists such as klipspringer and rock hyrax relied on rugged terrain for denning and escape cover. These associations mirror findings from the Andes, Himalayas, and southern Africa, where rugged landscapes sustain prey refuges and carnivore populations while excluding bulk grazers (Smith et al. 2019; Mann et al. 2020; Singh et al. 2022; Hinde et al. 2023; Ghimirey et al. 2024).

Temporal interactions were also evident. Hartebeest avoided rugged areas during the day but not at night, suggesting diel adjustments in predator avoidance, as similarly reported for antelope in savanna landscapes (Davies et al. 2016). Such examples underscore that ruggedness, while weak at a community scale, is ecologically critical for specialist herbivores and carnivores.

### 4.5 Landscape Units

Catchments divided into distinct landscape units are characterized by specific dominant first‐order hydrological processes, such as vegetation, hydrology, and topography, and enhance our understanding of ecological functions (Savenije 2010; Gao et al. 2014). Previous studies in the Baviaanskloof catchment highlighted that valley bottoms and lower hillslopes provide ecosystem services more efficiently than mountaintops (Petz et al. 2014; Glenday 2015). However, current findings indicate that valleys and floodplains are crucial biodiversity hotspots for large mammals, showing the highest proportion of capture events and species richness, indicating both frequent use and broad taxonomic representation (Webster et al. 2021; Snider et al. 2024).

Floodplains, with readily available water resources, attract species like aardvark, African clawless otters, Cape grysbok, vervet monkey, and bat‐eared fox, while valleys support species like honey badgers, klipspringer, and black‐backed jackals, which benefit from structural complexity and resource availability. These valleys act as sinks for water runoff and nutrients in semi‐arid regions, creating microhabitats with enhanced water and forage availability, thus supporting both generalist and specialist species and playing a vital role in biodiversity conservation (Ludwig et al. 2005; Viviroli and Weingartner 2004; Yang et al. 2020).

In contrast, high plateaus, while accommodating specialized species such as bushbuck, Cape porcupine, and common duiker, showed the lowest proportion of capture events and species richness. These results highlight the ecological complementarity of landscape units, with lower elevations and valley bottoms acting as resource sinks and refuges, and uplands supporting more specialized fauna (Scott et al. 2001; Lagendijk et al. 2015; Drouilly et al. 2018).

### 4.6 Limitations and Recommendations for Mammal Occupancy Studies

While this study offers valuable insights into the influence of topographic complexity on mammal occupancy patterns, several limitations warrant acknowledgement. The temporal coverage of the camera trap surveys (~2 years) may not fully capture daily, seasonal, or inter‐annual variations in species behavior and resource use, crucial for understanding patterns over longer periods (Si et al. 2014; Kays et al. 2020). Incorporating analyses of temporal activity patterns, such as daily and seasonal fluctuations, could provide deeper insights into how mammals navigate and utilize topographically complex landscapes, especially for nocturnal species which may adjust their behavior in response to anthropogenic activity, changes in predator–prey dynamics or environmental risks during specific periods (Webster et al. 2021; Sosibo et al. 2023; Bouderka et al. 2023; Beukes et al. 2025a). Furthermore, while camera placement was designed to capture diverse topographic gradients, the reliance on trails and accessible terrain may introduce sampling bias (Mann et al. 2020; Hofmeester et al. 2021). Finally, the occupancy models used assume site closure within survey periods, a condition that may not hold for highly mobile species or those with large home ranges (Efford and Dawson 2012). Although Bayesian hierarchical modeling helps account for imperfect detection by incorporating species‐specific detection probabilities, the multispecies occupancy framework estimates habitat use for the observed community and does not explicitly model total or asymptotic species richness via latent community size parameters or data augmentation. Further exploration of temporal autocorrelation in species‐specific capture events could refine occupancy estimates and improve their accuracy (MacKenzie et al. 2002; Kéry and Royle 2016). Incorporating these factors could lead to a more comprehensive understanding and more effective conservation strategies tailored to the needs of diverse mammal communities (Montgomery, Ortiz‐Calo, and Heit, 2020; Sosibo et al. 2023).

## 5 Conclusion

Floodplains, valleys, gentle slopes, low‐ruggedness terrain, and south‐facing aspects emerge as critical habitats for sustaining diverse mammal communities, offering ecological stability, resource availability, and high species richness. In contrast, extreme features such as steep slopes, high solar gain, and rugged terrains support fewer species overall but remain essential for specialized taxa adapted to these challenging environments. This underscores the importance of maintaining a topographic mosaic in semi‐arid mountain catchments, where both generalist and specialist species depend on different facets of landscape complexity (Sultaire et al. 2023). While broad‐scale assessments of mammal community space use can obscure the microhabitat preferences that only become apparent at finer resolutions (Montgomery et al. 2020), our findings highlight the need to identify species‐specific drivers of habitat use for effective conservation. Management strategies in semi‐arid mountain catchments should therefore integrate both community‐ and species‐level perspectives, ensuring that landscape heterogeneity is preserved to support ecological resilience across taxa (Beukes et al. 2025a). Embedding these insights into long‐term planning, such as the design of ecological corridors (Lichtenberg et al. 2025), will be vital for conserving biodiversity under the dynamic interplay of topographic complexity and environmental change.

## Author Contributions

**Maya Beukes:** conceptualization (equal), data curation (equal), formal analysis (equal), funding acquisition (equal), investigation (equal), methodology (equal), project administration (equal), resources (equal), software (equal), visualization (equal), writing – original draft (equal), writing – review and editing (equal). **Travis Perry:** conceptualization (equal), methodology (equal), project administration (equal), resources (equal), supervision (equal), writing – review and editing (equal). **Dan Parker:** conceptualization (equal), methodology (equal), project administration (equal), supervision (equal), writing – review and editing (equal). **Nokubonga Mgqatsa:** conceptualization (equal), funding acquisition (equal), methodology (equal), project administration (equal), supervision (equal), writing – review and editing (equal).

## Funding

This research was supported by the National Research Foundation, South Africa, under the auspices of Rhodes University.

## Conflicts of Interest

The authors declare no conflicts of interest.

## Supporting information


**Appendix S1:** Representation of topographic complexity (landscape units, aspect, ruggedness, solar gain, and slope) within the Baviaanskloof catchment and the distribution of camera trap deployments across the study area.
**Appendix S2:** Topographical landscape units.
**Appendix S3:** Summary of camera deployments, detections, and community occupancy.
**Appendix S4:** List of mammal species recorded during the camera‐trap survey, total number of independent capture events, and number of camera trap sites at which each species was recorded.
**Appendix S5:** Posterior summaries of species occupancy and detection probabilities across topographic covariates.
**Appendix S6:** Occupancy probabilities for mammal species across five landscape categories.
**Appendix S7:** Detailed discussion on species‐specific habitat preferences.

## Data Availability

The camera trap capture event data supporting this study are publicly available in the Zenodo repository at https://doi.org/10.5281/zenodo.18392835.

## References

[ece373100-bib-0001] Alderman, J. , and S. A. Hinsley . 2007. “Modelling the Third Dimension: Incorporating Topography Into the Movement Rules of an Individual‐Based Spatially Explicit Population Model.” Ecological Complexity 4, no. 4: 169–181. 10.1016/j.ecocom.2007.06.009.

[ece373100-bib-0002] Andrews, P. , and E. M. O'Brien . 2000. “Climate, Vegetation, and Predictable Gradients in Mammal Species Richness in Southern Africa.” Journal of Zoology 251, no. 2: 205–231. 10.1111/j.1469-7998.2000.tb00605.x.

[ece373100-bib-0003] Badano, E. I. , L. A. Cavieres , M. A. Molina‐Montenegro , and C. L. Quiroz . 2005. “Slope Aspect Influences Plant Association Patterns in the Mediterranean Matorral of Central Chile.” Journal of Arid Environments 62, no. 1: 93–108. 10.1016/j.jaridenv.2004.10.012.

[ece373100-bib-0004] Badgley, C. , T. M. Smiley , R. Terry , et al. 2017. “Biodiversity and Topographic Complexity: Modern and Geohistorical Perspectives.” Trends in Ecology & Evolution 32, no. 3: 211–226. 10.1016/j.tree.2016.12.010. 28196688 PMC5895180

[ece373100-bib-0005] Bennie, J. , B. Huntley , A. Wiltshire , M. O. Hill , and R. Baxter . 2008. “Slope, Aspect and Climate: Spatially Explicit and Implicit Models of Topographic Microclimate in Chalk Grassland.” Ecological Modelling 216, no. 1: 47–59. 10.1016/j.ecolmodel.2008.04.010.

[ece373100-bib-0006] Beukes, M. , T. Perry , D. Parker , O. Beukes , and N. Mgqatsa . 2025a. “Biodiversity Beyond Protected Areas: Mammal Responses to Land‐Use and Vegetation in a Semi‐Arid Mixed‐Use Landscape.” Preprint. Available at SSRN 5275969. 10.2139/ssrn.5275969.

[ece373100-bib-0007] Beukes, M. , T. Perry , D. M. Parker , and N. Mgqatsa . 2025b. “Refining Camera Trap Surveys for Mammal Detection and Diversity Assessment in the Baviaanskloof Catchment, South Africa.” Wild 2, no. 2: 15. 10.3390/wild2020015.

[ece373100-bib-0008] Bolstad, P. 2016. GIS Fundamentals: A First Text on Geographic Information Systems. Eider Press.

[ece373100-bib-0009] Boshoff, A. F. 2005. “The Baviaanskloof Mega‐Reserve: An Environmentally, Socially and Economically Sustainable Conservation and Development Initiative.” In Terrestrial Ecology Research Unit Report: 52. Nelson Mandela Metropolitan University.

[ece373100-bib-0010] Bouchet, P. J. , J. J. Meeuwig , C. P. Salgado Kent , T. B. Letessier , and C. K. Jenner . 2015. “Topographic Determinants of Mobile Vertebrate Predator Hotspots: Current Knowledge and Future Directions.” Biological Reviews 90, no. 3: 699–728. 10.1111/brv.12130. 25125200

[ece373100-bib-0011] Bouderka, S. , T. W. Perry , D. M. Parker , M. Beukes , and N. Mgqatsa . 2023. “Count Me in: Leopard Population Density in an Area of Mixed Land‐Use, Eastern Cape, South Africa.” African Journal of Ecology 61, no. 1: 228–232. 10.1111/aje.13078.

[ece373100-bib-0012] Buckland, S. T. , A. E. Magurran , R. E. Green , and R. M. Fewster . 2005. “Monitoring Change in Biodiversity Through Composite Indices.” Philosophical Transactions of the Royal Society, B: Biological Sciences 360, no. 1454: 243–254. 10.1098/rstb.2004.1589. PMC1569463 15814343

[ece373100-bib-0013] Burton, A. C. , E. Neilson , D. Moreira , et al. 2015. “Wildlife Camera Trapping: A Review and Recommendations for Linking Surveys to Ecological Processes.” Journal of Applied Ecology 52, no. 3: 675–685. 10.1111/1365-2664.12432.

[ece373100-bib-0014] Colyn, R. B. , F. G. T. Radloff , and M. J. O'Riain . 2018. “Camera Trapping Mammals in the Scrubland's of the Cape Floristic Kingdom—The Importance of Effort, Spacing and Trap Placement.” Biodiversity and Conservation 27: 503–520. 10.1007/s10531-017-1448-z.

[ece373100-bib-0015] Davies, A. B. , C. J. Tambling , G. I. Kerley , and G. P. Asner . 2016. “Limited Spatial Response to Direct Predation Risk by African Herbivores Following Predator Reintroduction.” Ecology and Evolution 6, no. 16: 5728–5748. 10.1002/ece3.2312. 27547350 PMC4983587

[ece373100-bib-0016] del Río‐Mena, T. , L. Willemen , A. Vrieling , A. Snoeys , and A. Nelson . 2021. “Long‐Term Assessment of Ecosystem Services at Ecological Restoration Sites Using Landsat Time Series.” PLoS One 16, no. 6: e0243020. 10.1371/journal.pone.0243020. 34161335 PMC8221468

[ece373100-bib-0017] Dilts, T. E. , M. E. Blum , K. T. Shoemaker , P. J. Weisberg , and K. M. Stewart . 2023. “Improved Topographic Ruggedness Indices More Accurately Model Fine‐Scale Ecological Patterns.” Landscape Ecology 38, no. 6: 1395–1410. 10.1007/s10980-023-01646-6.

[ece373100-bib-0018] Dobrowski, S. Z. 2011. “A Climatic Basis for Microrefugia: The Influence of Terrain on Climate.” Global Change Biology 17, no. 2: 1022–1035. 10.1111/j.1365-2486.2010.02263.x.

[ece373100-bib-0019] Dorazio, R. M. , and J. A. Royle . 2005. “Estimating Size and Composition of Biological Communities by Modeling the Occurrence of Species.” Journal of the American Statistical Association 100, no. 470: 389–398. 10.1198/016214505000000015.

[ece373100-bib-0020] Dorner, B. , K. Lertzman , and J. Fall . 2002. “Landscape Pattern in Topographically Complex Landscapes: Issues and Techniques for Analysis.” Landscape Ecology 17, no. 8: 729–743.

[ece373100-bib-0021] Drouilly, M. , A. Clark , and M. J. O'Riain . 2018. “Multi‐Species Occupancy Modelling of Mammal and Ground Bird Communities in Rangeland in the Karoo: A Case for Dryland Systems Globally.” Biological Conservation 224: 16–25. 10.1016/j.biocon.2018.05.013.

[ece373100-bib-0022] Efford, M. G. , and D. K. Dawson . 2012. “Occupancy in Continuous Habitat.” Ecosphere 3, no. 4: 1–15. 10.1890/ES11-00308.1.

[ece373100-bib-0023] Euston‐Brown, D. 2006. “Baviaanskloof Mega‐Reserve Project: Vegetation Mapping Contract: Report on Methodology, Vegetation Classification and Short Descriptions of Habitat Units.” Unpublished report. South Africa.

[ece373100-bib-0024] Farr, T. G. , P. A. Rosen , E. Caro , et al. 2007. “The Shuttle Radar Topography Mission.” Reviews of Geophysics 45, no. 2: RG2004. 10.1029/2005RG000183.

[ece373100-bib-0025] Fine, P. V. 2015. “Ecological and Evolutionary Drivers of Geographic Variation in Species Diversity.” Annual Review of Ecology, Evolution, and Systematics 46, no. 1: 369–392. 10.1146/annurev-ecolsys-112414-054102.

[ece373100-bib-0026] Florinsky, I. V. 2012. Digital Terrain Analysis in Soil Science and Geology. Elsevier.

[ece373100-bib-0027] Fu, P. , and P. M. Rich . 2002. “A Geometric Solar Radiation Model With Applications in Agriculture and Forestry.” Computers and Electronics in Agriculture 37, no. 1–3: 25–35. 10.1016/S0168-1699(02)00115-1.

[ece373100-bib-0028] Gao, H. , M. Hrachowitz , S. J. Schymanski , F. Fenicia , N. Sriwongsitanon , and H. H. G. Savenije . 2014. “Climate Controls How Ecosystems Size the Root Zone Storage Capacity at Catchment Scale.” Geophysical Research Letters 41, no. 22: 7916–7923. 10.1002/2014GL061668.

[ece373100-bib-0029] Gedir, J. V. , J. W. Cain III , T. L. Swetnam , P. R. Krausman , and J. R. Morgart . 2020. “Extreme Drought and Adaptive Resource Selection by a Desert Mammal.” Ecosphere 11, no. 7: e03175. 10.1002/ecs2.3175.

[ece373100-bib-0030] Gelman, A. , J. Hwang , and A. Vehtari . 2014. “Understanding Predictive Information Criteria for Bayesian Models.” Statistics and Computing 24: 997–1016. 10.1007/s11222-013-9416-2.

[ece373100-bib-0031] Ghimirey, Y. , R. Acharya , and J. Mintz . 2024. “Factors Affecting Mammalian Occupancy and Species Richness in Annapurna Conservation Area, Nepal.” Ecology and Evolution 14, no. 11: e70572. 39563699 10.1002/ece3.70572 PMC11573729

[ece373100-bib-0032] Glenday, J. A. 2015. Modeling the Hydrologic Impacts of Vegetation and Channel Network Change for a Semi‐Arid, Mountainous, Meso‐Scale Catchment: The Baviaanskloof, South Africa. University of California.

[ece373100-bib-0033] GRASS Development Team . 2024. Geographic Resources Analysis Support System (GRASS) Software, Version 8.4. Open Source Geospatial Foundation. https://grass.osgeo.org.

[ece373100-bib-0034] Green, D. G. , N. Klomp , G. Rimmington , and S. Sadedin . 2006. Complexity in Landscape Ecology. Vol. 217. Springer. 10.1007/978-3-030-46773-9.

[ece373100-bib-0035] Greenberg, S. , T. Godin , and J. Whittington . 2019. “Design Patterns for Wildlife‐Related Camera Trap Image Analysis.” Ecology and Evolution 9, no. 24: 13706–13730. 10.1002/ece3.5767. 31938476 PMC6953665

[ece373100-bib-0036] Heit, D. R. , C. C. Wilmers , W. Ortiz‐Calo , and R. A. Montgomery . 2023. “Incorporating Vertical Dimensionality Improves Biological Interpretation of Hidden Markov Model Outputs.” Oikos 2023, no. 5: e09820. 10.1111/oik.09820.

[ece373100-bib-0037] Hinde, K. , A. Wilkinson , S. Tokota , R. Amin , M. J. O'Riain , and K. S. Williams . 2023. “Leopard Density and the Ecological and Anthropogenic Factors Influencing Density in a Mixed‐Use Landscape in the Western Cape, South Africa.” PLoS One 18, no. 10: e0293445. 10.1371/journal.pone.0293445. 37889916 PMC10610481

[ece373100-bib-0038] Hofmeester, T. R. , N. H. Thorsen , J. P. Cromsigt , et al. 2021. “Effects of Camera‐Trap Placement and Number on Detection of Members of a Mammalian Assemblage.” Ecosphere 12, no. 7: e03662. 10.1002/ecs2.3662.

[ece373100-bib-0039] Holmes, P. J. 2012. Southern African Geomorphology: Recent Trends and New Directions. African Sun Media.

[ece373100-bib-0040] Jarvis, A. , H. I. Reuter , and A. E. Nelson . 2008. “Hole‐Filled Seamless SRTM Data V4. International Centre for Tropical Agriculture (CIAT).” http://srtm.csi.cgiar.org.

[ece373100-bib-0041] Jenness, J. S. 2004. “Calculating Landscape Surface Area From Digital Elevation Models.” Wildlife Society Bulletin 32, no. 3: 829–839. 10.2193/0091-7648(2004)032[0829:CLSAFD]2.0.CO;2.

[ece373100-bib-0042] Kays, R. , B. S. Arbogast , M. Baker‐Whatton , et al. 2020. “An Empirical Evaluation of Camera Trap Study Design: How Many, How Long and When?” Methods in Ecology and Evolution 11, no. 6: 700–713. 10.2478/vzoo-2019-0004.

[ece373100-bib-0043] Keppel, G. , and G. W. Wardell‐Johnson . 2012. “Refugia: Keys to Climate Change Management.” Global Change Biology 18, no. 8: 2389–2391. 10.1111/j.1365-2486.2012.02729.x.

[ece373100-bib-0095] Kéry, M. , and J. A. Royle . 2016. “Applied Hierarchical Modeling in Ecology: Analysis of Distribution, Abundance and Species Richness in R and BUGS.” In Volume 1: Prelude and Static Models. Academic Press.

[ece373100-bib-0044] Kéry, M. , and J. A. Royle . 2021. Applied Hierarchical Modeling in Ecology: Analysis of Distribution, Abundance and Species Richness in R and BUGS. Dynamic and Advanced Models. Vol. 2. Academic Press.

[ece373100-bib-0045] Kok, A. 2016. Land‐Use Effects on Mammal Communities in the Fish‐Kowie Corridor, Eastern Cape, South Africa, With Particular Reference to Carnivores. Masters thesis, Rhodes University, South Africa.

[ece373100-bib-0046] Lagendijk, D. G. , M. Thaker , W. F. De Boer , B. R. Page , H. H. Prins , and R. Slotow . 2015. “Change in Mesoherbivore Browsing Is Mediated by Elephant and Hillslope Position.” PLoS One 10, no. 6: e0128340. 10.1371/journal.pone.0128340. 26083248 PMC4471177

[ece373100-bib-0047] Lawson, C. R. , J. Bennie , J. A. Hodgson , C. D. Thomas , and R. J. Wilson . 2014. “Topographic Microclimates Drive Microhabitat Associations at the Range Margin of a Butterfly.” Ecography 37, no. 8: 732–740. 10.1111/ecog.00535.

[ece373100-bib-0048] Lemenkova, P. 2022. “GRASS GIS Scripts for Satellite Image Analysis by Raster Calculations Using Modules r. Mapcalc, d. Rgb, r. Slope. Aspect.” Tehnički Vjesnik 29, no. 6: 1956–1963. 10.17559/TV-20220322091846.

[ece373100-bib-0049] Li, X. , W. V. Bleisch , and X. Jiang . 2018. “Using Large Spatial Scale Camera Trap Data and Hierarchical Occupancy Models to Evaluate Species Richness and Occupancy of Rare and Elusive Wildlife Communities in Southwest China.” Diversity and Distributions 24, no. 11: 1560–1572. 10.1111/ddi.12792.

[ece373100-bib-0050] Lichtenberg, D. , E. Kreuzberg , K. von Dürckheim , et al. 2025. “Landscape Connectivity for Biodiversity Conservation: A Mammal‐Based Multi‐Species Corridor Approach for the Eden to Addo Corridor Initiative, South Africa.” Biodiversity and Conservation 34: 1–21. 10.1007/s10531-025-03140-8.

[ece373100-bib-0051] Ludwig, J. A. , B. P. Wilcox , D. D. Breshears , D. J. Tongway , and A. C. Imeson . 2005. “Vegetation Patches and Runoff–erosion as Interacting Ecohydrological Processes in Semiarid Landscapes.” Ecology 86, no. 2: 288–297. 10.1890/03-0569.

[ece373100-bib-0052] MacKenzie, D. I. , J. D. Nichols , G. B. Lachman , S. Droege , J. A. Royle , and C. A. Langtimm . 2002. “Estimating Site Occupancy Rates When Detection Probabilities Are Less Than One.” Ecology 83: 2248–2255. 10.1890/0012-9658(2002)083[2248:ESORWD]2.0.CO;2.

[ece373100-bib-0053] Mann, G. K. , M. J. O'Riain , and D. M. Parker . 2020. “A Leopard's Favourite Spots: Habitat Preference and Population Density of Leopards in a Semi‐Arid Biodiversity Hotspot.” Journal of Arid Environments 181: 104218. 10.1016/j.jaridenv.2020.104218.

[ece373100-bib-0054] Marchese, C. 2015. “Biodiversity Hotspots: A Shortcut for a More Complicated Concept.” Global Ecology and Conservation 3: 297–309. 10.1016/j.gecco.2014.12.008.

[ece373100-bib-0055] McCain, C. M. 2005. “Elevational Gradients in Diversity of Small Mammals.” Ecology 86, no. 2: 366–372. 10.1890/03-3147.

[ece373100-bib-0056] Montgomery, R. A. , W. Ortiz‐Calo , and D. R. Heit . 2020. “Integrating the Multi‐Domainal and Multi‐Dimensional Nature of Animal Movement Into Ecological Modelling.” Ecological Modelling 436: 109220. 10.1016/j.ecolmodel.2020.109220.

[ece373100-bib-0057] Morelli, T. L. , C. Daly , S. Z. Dobrowski , et al. 2016. “Managing Climate Change Refugia for Climate Adaptation.” PLoS One 11, no. 8: e0159909. 10.1371/journal.pone.0159909. 27509088 PMC4980047

[ece373100-bib-0058] Ndeketeya, A. 2012. An Assessment of the Economic Water Use Efficiency and Productivity of the Upstream and Downstream Catchments' Agricultural Production, South Africa. Wageningen University.

[ece373100-bib-0059] Neteler, M. , and H. Mitasova . 2008. Open Source GIS: A GRASS GIS Approach. Springer. 10.1007/978-0-387-68574-8.

[ece373100-bib-0060] Niedballa, J. , R. Sollmann , A. B. Mohamed , J. Bender , and A. Wilting . 2015. “Defining Habitat Covariates in Camera‐Trap Based Occupancy Studies.” Scientific Reports 5, no. 1: 17041. 10.1038/srep17041. 26596779 PMC4657010

[ece373100-bib-0061] North, M. A. 2009. A Method for Implementing a Statistically Significant Number of Data Classes in the Jenks Algorithm. In 2009 Sixth International Conference on Fuzzy Systems and Knowledge Discovery. Vol. 1, 35–38. IEEE. 10.1109/FSKD.2009.319.

[ece373100-bib-0062] Petz, K. , J. Glenday , and R. Alkemade . 2014. “Land Management Implications for Ecosystem Services in a South African Rangeland.” Ecological Indicators 45: 692–703. 10.1016/j.ecolind.2014.05.023.

[ece373100-bib-0063] Plummer, M. 2003. “JAGS: A Program for Analysis of Bayesian Graphical Models Using Gibbs Sampling.” Pages 1–10 in Proceedings of the 3rd International Workshop on Distributed Statistical Computing, Vienna, Austria.

[ece373100-bib-0064] Powell, M. J. 2009. Restoration of Degraded Subtropical Thickets in the Baviaanskloof Megareserve, South Africa: The Role of Carbon Stocks and Portulacaria afra Survivorship. Master's Thesis, Rhodes University.

[ece373100-bib-0097] R Core Team . 2022. R: A Language and Environment for Statistical Computing (Version 4.2.2). R Foundation for Statistical Computing.

[ece373100-bib-0098] Rahbek, C. , M. K. Borregaard , A. Antonelli , et al. 2019. “Building Mountain Biodiversity: Geological and Evolutionary Processes.” Science 365, no. 6458: 1114–1119. 10.1126/science.aax0151. 31515384

[ece373100-bib-0065] Reuter, H. I. , A. Nelson , and A. Jarvis . 2007. “An Evaluation of Void Filling Interpolation Methods for SRTM Data.” International Journal of Geographic Information Science 21, no. 9: 983–1008. 10.1080/13658810601169899.

[ece373100-bib-0066] Riley, S. J. , S. D. DeGloria , and R. Elliot . 1999. “Index That Quantifies Topographic Heterogeneity.” Intermountain Journal of Sciences 5, no. 1–4: 23–27.

[ece373100-bib-0067] Roell, Y. E. , J. G. Phillips , and C. E. Parent . 2021. “Effect of Topographic Complexity on Species Richness in the Galápagos Islands.” Journal of Biogeography 48, no. 10: 2645–2657. 10.1111/jbi.14230.

[ece373100-bib-0068] Rota, C. T. , M. A. R. Ferreira , R. W. Kays , et al. 2016. “A Multispecies Occupancy Model for Two or More Interacting Species.” Methods in Ecology and Evolution 7: 1164–1173. 10.1111/2041-210X.12587.

[ece373100-bib-0069] Rowe‐Rowe, D. T. 1983. “Habitat Preferences of Five Drakensberg Antelopes.” South African Journal of Wildlife Research 13, no. 1: 1–8.

[ece373100-bib-0070] Sappington, J. M. , K. M. Longshore , and D. B. Thompson . 2007. “Quantifying Landscape Ruggedness for Animal Habitat Analysis: A Case Study Using Bighorn Sheep in the Mojave Desert.” Journal of Wildlife Management 71, no. 5: 1419–1426. 10.2193/2005-723.

[ece373100-bib-0071] Savenije, H. H. 2010. “HESS Opinions ‘Topography Driven Conceptual Modelling (FLEX‐Topo)’.” Hydrology and Earth System Sciences 14, no. 12: 2681–2692. 10.5194/hess-14-2681-2010.

[ece373100-bib-0072] Scott, J. M. , F. W. Davis , R. G. McGhie , R. G. Wright , C. Groves , and J. Estes . 2001. “Nature Reserves: Do They Capture the Full Range of America's Biological Diversity?” Ecological Applications 11, no. 4: 999–1007. 10.1890/1051-0761(2001)011[0999:NRDTCT]2.0.CO;2.

[ece373100-bib-0073] Sears, M. W. , and M. J. Angilletta Jr. 2015. “Costs and Benefits of Thermoregulation Revisited: Both the Heterogeneity and Spatial Structure of Temperature Drive Energetic Costs.” American Naturalist 185, no. 4: E94–E102. 10.1086/680008. 25811092

[ece373100-bib-0074] Shepard, E. L. , R. P. Wilson , W. G. Rees , E. Grundy , S. A. Lambertucci , and S. B. Vosper . 2013. “Energy Landscapes Shape Animal Movement Ecology.” American Naturalist 182, no. 3: 298–312. 10.1086/671257. 23933722

[ece373100-bib-0075] Si, X. , R. Kays , and P. Ding . 2014. “How Long Is Enough to Detect Terrestrial Animals? Estimating the Minimum Trapping Effort on Camera Traps.” PeerJ 2: e374. 10.7717/peerj.374. 24868493 PMC4017883

[ece373100-bib-0076] Singh, H. , A. Sharief , B. D. Joshi , et al. 2022. “Multi‐Species Occupancy Modeling Suggests Interspecific Interaction Among the Three Ungulate Species.” Scientific Reports 12, no. 1: 17602. 10.1038/s41598-022-20953-7. 36266303 PMC9584884

[ece373100-bib-0077] Skinner, J. D. , and C. T. Chimimba . 2005. The Mammals of the Southern African Subregion. 3rd ed. Cambridge University Press.

[ece373100-bib-0078] Smith, J. A. , E. Donadio , J. N. Pauli , M. J. Sheriff , and A. D. Middleton . 2019. “Integrating Temporal Refugia Into Landscapes of Fear: Prey Exploit Predator Downtimes to Forage in Risky Places.” Oecologia 189, no. 4: 883–890. 10.1007/s00442-019-04381-5. 30868375

[ece373100-bib-0079] Snider, M. H. , K. M. Helgen , H. S. Young , et al. 2024. “Shifting Mammal Communities and Declining Species Richness Along an Elevational Gradient on Mount Kenya.” Ecology and Evolution 14, no. 4: e11151. 10.1002/ece3.11151. 38601855 PMC11004549

[ece373100-bib-0080] Sollmann, R. , A. Mohamed , H. Samejima , and A. Wilting . 2013. “Risky Business or Simple Solution–Relative Abundance Indices From Camera‐Trapping.” Biological Conservation 159: 405–412. 10.1016/j.biocon.2012.12.025.

[ece373100-bib-0081] Sosibo, M. T. , D. A. Ehlers Smith , Y. C. Ehlers Smith , S. T. Gumede , S. P. Ngcobo , and C. T. Downs . 2023. “Influence of Microhabitat Structure on Large‐and Medium‐Sized Mammals in South African Forests.” African Journal of Ecology 61, no. 3: 617–627. 10.1111/aje.13149.

[ece373100-bib-0096] Spatial Ecology, L. L. C. 2012. “Geospatial Modelling Environment (Version 0.7.2 RC2).” Available at: http://www.spatialecology.com/gme. Accessed November 18, 2024.

[ece373100-bib-0082] Sultaire, S. M. , J. J. Millspaugh , P. J. Jackson , and R. A. Montgomery . 2023. “The Influence of Fine‐Scale Topography on Detection of a Mammal Assemblage at Camera Traps in a Mountainous Landscape.” Wildlife Biology 2023, no. 2: e01026. 10.1002/wlb3.01026.

[ece373100-bib-0083] Tambling, C. J. , L. Minnie , J. Meyer , et al. 2015. “Temporal Shifts in Activity of Prey Following Large Predator Reintroductions.” Behavioral Ecology and Sociobiology 69, no. 7: 1153–1161. 10.1007/s00265-015-1929-6.

[ece373100-bib-0084] Tarolli, P. 2014. “High‐Resolution Topography for Understanding Earth Surface Processes: Opportunities and Challenges.” Geomorphology 216: 295–312. 10.1016/j.geomorph.2014.03.008.

[ece373100-bib-0085] Theobald, D. M. , A. L. Jacob , P. R. Elsen , E. A. Beever , L. Ehlers , and J. Hilty . 2024. “Evaluating Ecosystem Protection and Fragmentation of the World's Major Mountain Regions.” Conservation Biology 38, no. 3: e14240. 10.1111/cobi.14240. 38407527

[ece373100-bib-0086] Thuiller, W. , F. Midgley , M. Rougeti , and R. Cowling . 2006. “Predicting Patterns of Plant Species Richness in Megadiverse South Africa.” Ecography 29, no. 5: 733–744. 10.1111/j.0906-7590.2006.04674.x.

[ece373100-bib-0087] Tobler, M. W. , S. E. Carrillo‐Percastegui , R. Leite Pitman , R. Mares , and G. Powell . 2008. “An Evaluation of Camera Traps for Inventorying Large‐and Medium‐Sized Terrestrial Rainforest Mammals.” Animal Conservation 11, no. 3: 169–178. 10.1111/j.1469-1795.2008.00169.x.

[ece373100-bib-0088] Tobler, M. W. , and G. V. Powell . 2013. “Estimating Jaguar Densities With Camera Traps: Problems With Current Designs and Recommendations for Future Studies.” Biological Conservation 159: 109–118. 10.1016/j.biocon.2012.12.009.

[ece373100-bib-0089] Van Luijk, G. , R. M. Cowling , M. J. P. M. Riksen , and J. Glenday . 2013. “Hydrological Implications of Desertification: Degradation of South African Semi‐Arid Subtropical Thicket.” Journal of Arid Environments 91: 14–21. 10.1016/j.jaridenv.2012.10.022.

[ece373100-bib-0090] Viviroli, D. , and R. Weingartner . 2004. “The Hydrological Significance of Mountains: From Regional to Global Scale.” Hydrology and Earth System Sciences 8, no. 6: 1017–1030. 10.5194/hess-8-1017-2004.

[ece373100-bib-0091] Webster, A. B. , M. E. Pretorius , and M. J. Somers . 2021. “The Determinants of Mesocarnivore Activity Patterns in Highveld Grassland and Riparian Habitats.” African Journal of Wildlife Research 51, no. 1: 178–192.

[ece373100-bib-0092] Wilson, J. P. , and J. C. Gallant . 2000. Terrain Analysis: Principles and Applications. Wiley.

[ece373100-bib-0093] Yang, J. , Y. A. El‐Kassaby , and W. Guan . 2020. “The Effect of Slope Aspect on Vegetation Attributes in a Mountainous Dry Valley, Southwest China.” Scientific Reports 10, no. 1: 16465. 10.1038/s41598-020-73496-0. 33020576 PMC7536199

[ece373100-bib-0094] Yu, F. , T. Wang , T. A. Groen , et al. 2015. “Multi‐Scale Comparison of Topographic Complexity Indices in Relation to Plant Species Richness.” Ecological Complexity 22: 93–101. 10.1016/j.ecocom.2015.02.007.

