# Supplementary material for: Light Paths and Dark Valleys: Topographic Complexity and Mammal Occupancy in a Semi‐Arid Mountain Landscape
Source: Ecol Evol. 2026 Feb 13;16(2):e73100. doi: 10.1002/ece3.73100 (PMC12904844; doi:10.1002/ece3.73100)
Supplement: Supplementary file 1 — Appendix S1: Representation of topographic complexity (landscape units, aspect, ruggedness, solar gain, and slope) within the Baviaanskloof catchment and the distribution of camera trap deployments across the study area. Appendix S2: Topographical landscape units. Appendix S3: Summary of camera deployments, detections, and community occupancy. Appendix S4: List of mammal species recorded during the camera‐trap survey, total number of independent capture events, and number of camera trap sites at which each species was recorded. Appendix S5: Posterior summaries of species occupancy and detection probabilities across topographic covariates. Appendix S6: Occupancy probabilities for mammal species across five landscape categories. Appendix S7: Detailed discussion on species‐specific habitat preferences. [file ECE3-16-e73100-s001.zip › light paths and dark valleys_supplementary material.docx]

# **Supplementary**

**Appendix S1:** Representation of topographic complexity (landscape units, aspect, ruggedness, solar gain, and slope) within the Baviaanskloof catchment and the distribution of camera trap deployments across the study area


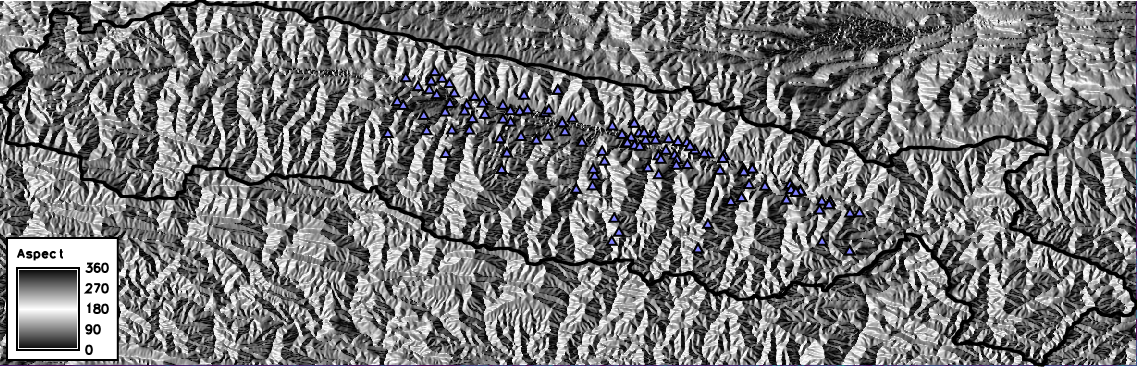


**b**


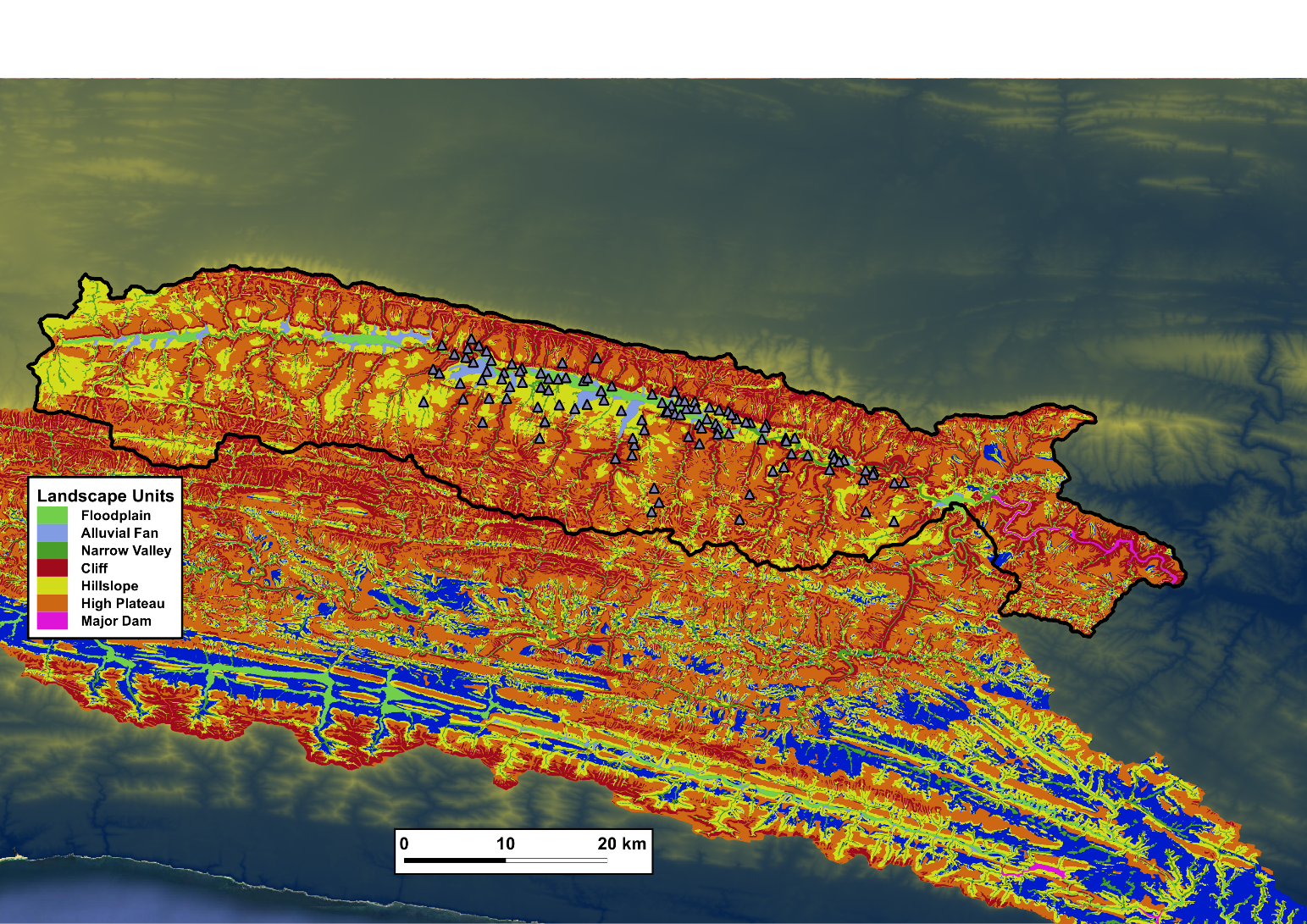


**a**


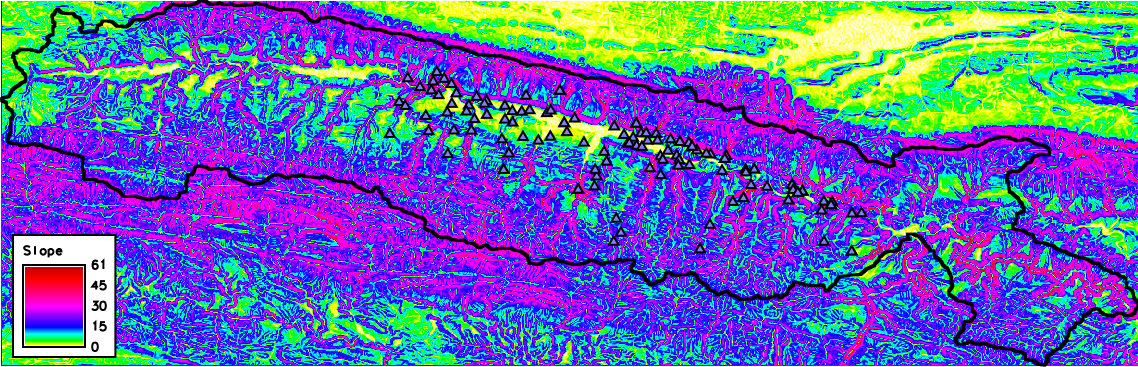


**e**


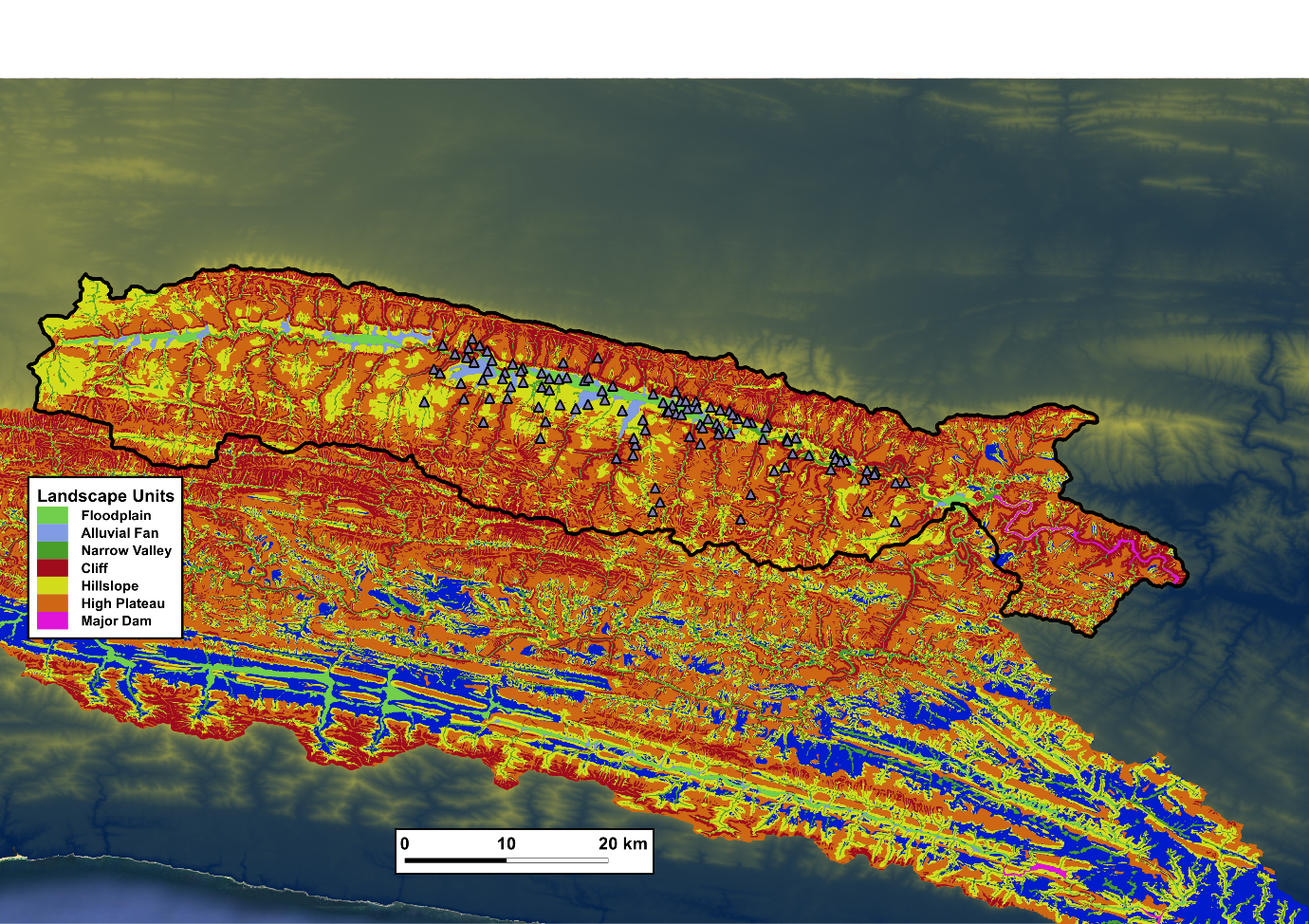

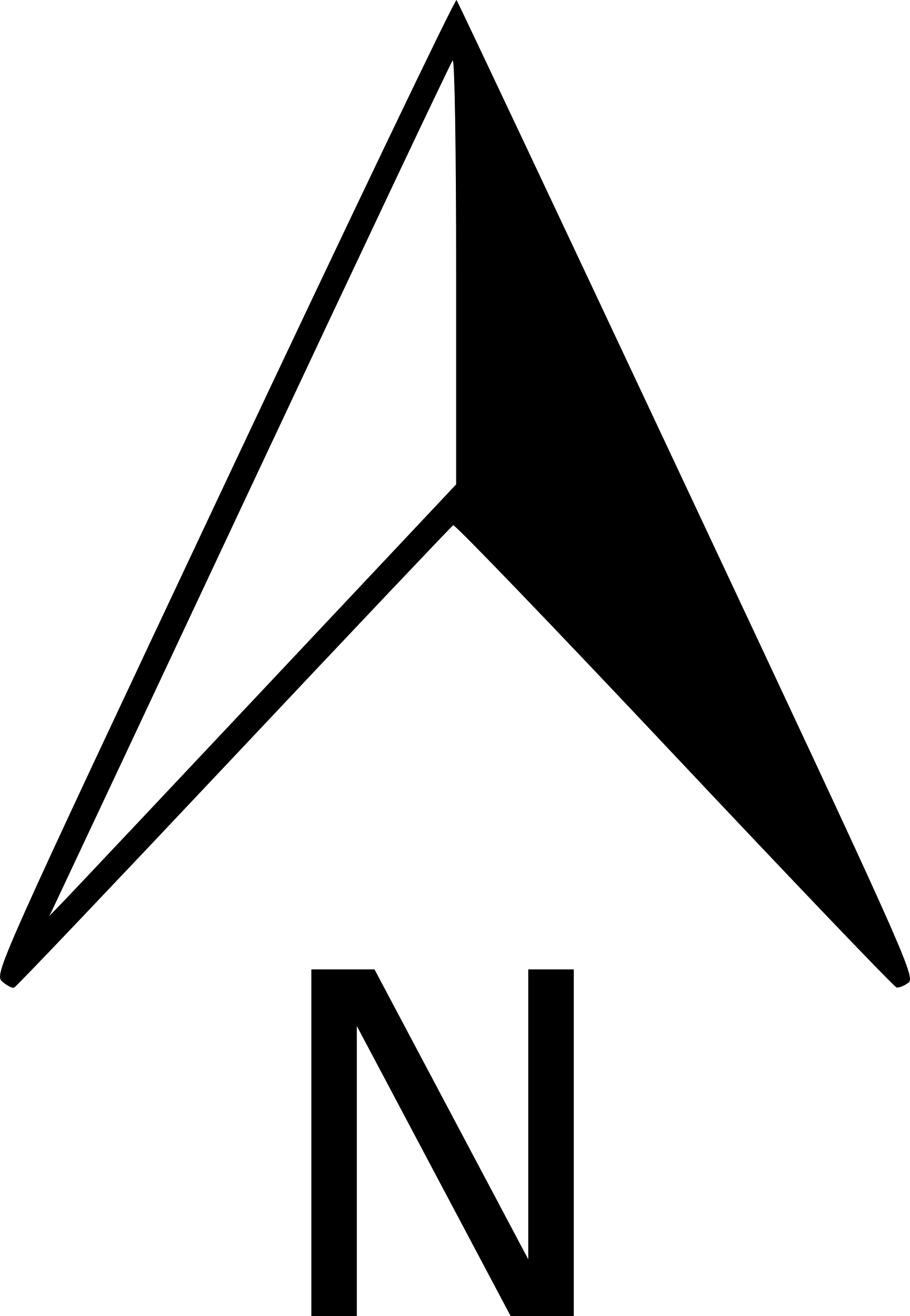

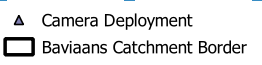

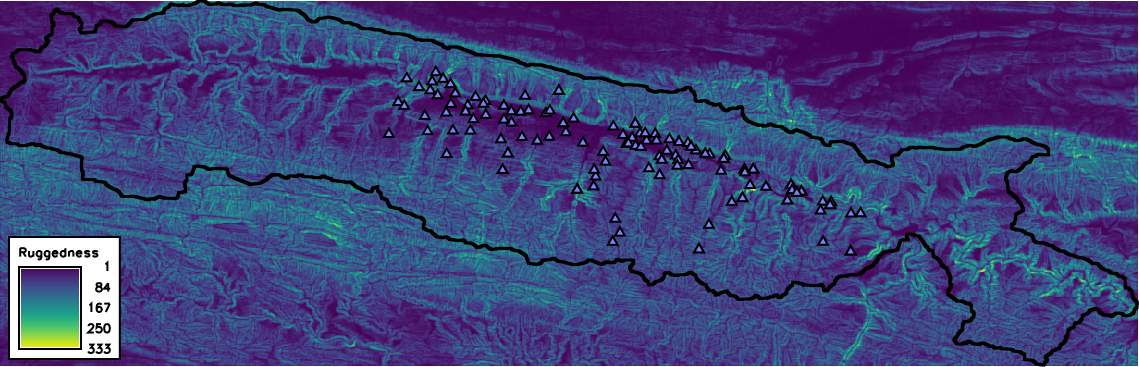


**c**


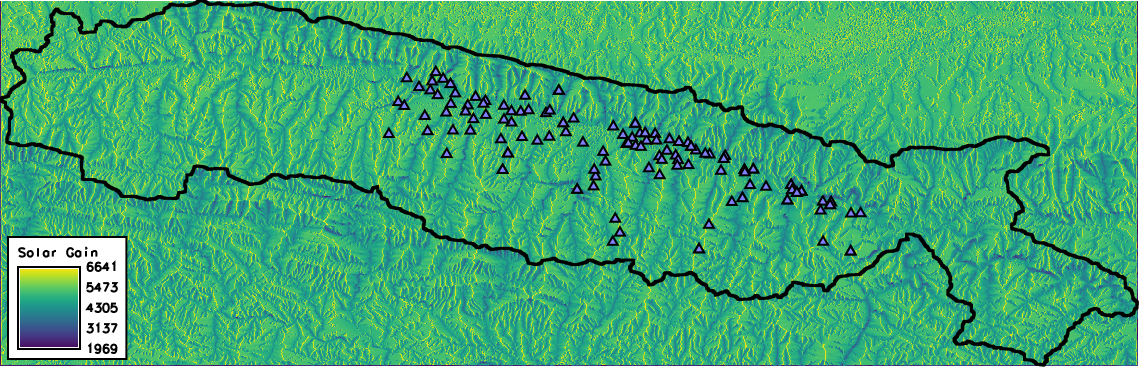


**d**

**Fig.** Representation of topographic complexity within the Baviaanskloof catchment: (a) landscape units, (b) aspect, (c) ruggedness, (d) solar gain, and (e) slope. Camera deployments were distributed across five landscape units (alluvial fan = 48 deployments, floodplain = 40, high plateau = 28, hillslope = 62, narrow valley = 24; Table 2). Aspect (1–365°) was grouped into four categories (north = 59 deployments, east = 41, south = 75, west = 27). Ruggedness (1–130) was mostly low (118 deployments), with fewer medium (65) and high (19). Solar gain (360–6400 Wh/m²) was classified into five levels (low = 37, medium-low = 76, medium-high = 61, high = 22, very high = 6 deployments). Slope (0–32°) was dominated by low (102 deployments) and medium (68), with fewer high (22) and very high (10).

**Appendix S2:** Topographical landscape units

| **Landscape unit** | **Area (km2)** | **Percent of catchment** |
| --- | --- | --- |
| **Tributary catchments** |  |  |
| Plateau | 357 | 29% |
| Hillslope | 494 | 40% |
| Cliff | 209 | 17% |
| Canyon floor | 118 | 10% |
| Subtotal (tributaries) | 1179 | 95% |
| **Central valley** |  |  |
| Floodplain | 44 | 4% |
| Alluvial fan | 12 | 1% |
| Subtotal (central valley) | 56 | 5% |
| **Catchment total** | **1234** | **100%** |

**Table** Topographical landscape units presented in the Baviaanskloof catchment (adapted from Glenday, 2015). Although seven geomorphological landscape units were identified, cliff habitats were excluded from subsequent analyses due to inaccessibility and the absence of camera trap deployments in these areas. As a result, six landscape units were retained for analysis.

**Appendix S3:** Summary of camera deployments, detections, and community occupancy

| **Category** | **Level** | **Deployments** | **Detections** | **Proportion** | **Species** | **Mean *ψ* (BCI)** |
| --- | --- | --- | --- | --- | --- | --- |
| landscape unit | valley | 30 | 1393 | 22.97 | 28 | 0.29 (0.05, 0.53) |
| landscape unit | alluvial fan | 21 | 1277 | 21.06 | 21 | 0.29 (0.07, 0.51) |
| landscape unit | floodplain | 27 | 1715 | 28.28 | 23 | 0.31 (0.08, 0.54) |
| landscape unit | hillslope | 34 | 1236 | 20.38 | 29 | 0.30 (0.07, 0.54) |
| landscape unit | high plateau | 17 | 443 | 7.31 | 22 | 0.26 (0.05, 0.48) |
| aspect | north | 38 | 1778 | 29.32 | 30 | 0.29 (0.07, 0.50) |
| aspect | south | 40 | 1590 | 26.22 | 28 | 0.33 (0.13, 0.54) |
| aspect | west | 13 | 540 | 8.91 | 24 | 0.24 (-0.00, 0.49) |
| aspect | east | 38 | 2156 | 35.55 | 27 | 0.28 (0.07, 0.49) |
| ruggedness | low | 83 | 3715 | 61.26 | 32 | 0.29 (0.08, 0.51) |
| ruggedness | medium | 36 | 1902 | 31.37 | 26 | 0.29 (0.09, 0.49) |
| ruggedness | high | 10 | 447 | 7.37 | 24 | 0.25 (0.07, 0.44) |
| solar gain | low | 34 | 1877 | 30.95 | 24 | 0.28 (0.07, 0.49) |
| solar gain | medium-low | 34 | 1759 | 29.01 | 24 | 0.26 (0.07, 0.45) |
| solar gain | medium-high | 44 | 1877 | 30.95 | 29 | 0.34 (0.13, 0.55) |
| solar gain | high | 17 | 551 | 9.09 | 23 | 0.24 (0.01, 0.46) |
| slope | low | 68 | 3987 | 65.75 | 30 | 0.29 (0.04, 0.54) |
| slope | medium | 31 | 1051 | 17.33 | 30 | 0.26 (0.05, 0.46) |
| slope | high | 22 | 669 | 11.03 | 24 | 0.34 (0.12, 0.59) |
| slope | very high | 8 | 357 | 5.89 | 20 | 0.27 (0.01, 0.52) |

**Table** Summary of camera deployments, species detections (species independent capture events), and community occupancy (*ψ*) estimates across landscape unit, aspect, ruggedness, solar gain, and slope categories. Independent capture events represent temporally filtered records (≥30 min separation) and are reported as descriptive summaries. Proportion of capture events refers to the proportion of total independent events recorded within each category. Mean community occupancy probabilities (ψ) with Bayesian credible intervals (BCIs) are derived from multispecies occupancy models

## **Appendix S4:** List of mammal species recorded during the camera-trap survey, total number of independent capture events, and number of camera trap sites at which each species was recorded.

| **Latin name** | **Common name** | **Taxonomic order** | **Captures** | **Sites** |
| --- | --- | --- | --- | --- |
| Papio ursinus | Chacma baboon | Omnivore | 6327 | 126 |
| Tragelaphus strepsiceros | greater kudu | Large Herbivore | 3886 | 112 |
| Tragelaphus sylvaticus | bushbuck | Large Herbivore | 2353 | 58 |
| Chlorocebus pygerythrus | vervet monkey | Omnivore | 785 | 62 |
| Syncerus caffer | Cape buffalo | Large Herbivore | 622 | 39 |
| Lepus saxatilis | scrub hare | Small Herbivore | 514 | 49 |
| Panthera pardus | leopard | Carnivore | 400 | 68 |
| Potamochoerus larvatus | bushpig | Large Herbivore | 357 | 49 |
| Raphicerus melanotis | Cape grysbok | Medium Herbivore | 248 | 43 |
| Antidorcas marsupialis | springbok | Medium Herbivore | 215 | 8 |
| Hystrix africaeaustralis | Cape porcupine | Medium Herbivore | 213 | 43 |
| Sylvicapra grimmia | common duiker | Medium Herbivore | 182 | 25 |
| Oreotragus oreotragus | klipspringer | Medium Herbivore | 150 | 25 |
| Redunca fulvorufula | mountain reedbuck | Medium Herbivore | 142 | 22 |
| Equus zebra | Cape mountain zebra | Large Herbivore | 91 | 13 |
| Otocyon megalotis | bat-eared fox | Insectivore | 74 | 12 |
| Procavia capensis | rock hyrax | Small Herbivore | 59 | 12 |
| Genetta tigrina | large-spotted genet | Carnivore | 58 | 23 |
| Taurotragus oryx | eland | Large Herbivore | 47 | 5 |
| Caracal caracal | caracal | Carnivore | 43 | 25 |
| Proteles cristata | aardwolf | Insectivore | 40 | 10 |
| Mellivora capensis | honey badger | Carnivore | 36 | 24 |
| Herpestes pulverulentus | Cape grey mongoose | Insectivore | 33 | 22 |
| Aepyceros melampus | impala | Large Herbivore | 30 | 6 |
| Damaliscus pygargus | bontebok | Large Herbivore | 28 | 5 |
| Pronolagus saundersiae | Smith's red rock hare | Small Herbivore | 23 | 5 |
| Canis mesomelas | black-backed jackal | Carnivore | 21 | 16 |
| Pelea capreolus | grey rhebok | Medium Herbivore | 15 | 2 |
| Felis silvestris lybica | African wild cat | Carnivore | 14 | 7 |
| Alcelaphus buselaphus | red hartebeest | Large Herbivore | 14 | 7 |
| Ictonyx striatus | polecat | Carnivore | 10 | 4 |
| Orycteropus afer | aardvark | Insectivore | 6 | 4 |
| Aonyx capensis | African clawless otter | Carnivore | 5 | 3 |

**Table:** List of mammal species recorded during the camera-trap survey, including scientific and common names, taxonomic grouping, total number of independent capture events, and the number of camera-trap sites at which each species was recorded. Capture events represent temporally independent records (≥30 min separation) aggregated across all survey periods. Species are ordered in descending order of total capture events.

## **Appendix S5:** Posterior summaries of species occupancy and detection probabilities across topographic covariates

| **Species** | **Covariate Name** | **Covariate Level** | **Mean Occupancy** | **SD Occupancy** | **lower BCI** | **upper BCI** |
| --- | --- | --- | --- | --- | --- | --- |
| aardvark | landscape_cat | floodplain | 0.92 | 0.06 | 0.81 | 1.03 |
| aardvark | landscape_cat | hillslope | 0.17 | 0.08 | 0.02 | 0.32 |
| aardvark | rugged_cat | high | 0.92 | 0.08 | 0.76 | 1.08 |
| aardvark | solar_cat | medium high | 0.79 | 0.09 | 0.61 | 0.97 |
| aardvark | aspect_cat | south | 0.95 | 0.03 | 0.89 | 1.02 |
| aardvark | slope_cat | high | 0.92 | 0.05 | 0.81 | 1.03 |
| aardwolf | landscape_cat | alluvial fan | 0.42 | 0.1 | 0.22 | 0.61 |
| aardwolf | landscape_cat | valley | 0.5 | 0.1 | 0.3 | 0.7 |
| aardwolf | landscape_cat | floodplain | 0.34 | 0.1 | 0.15 | 0.52 |
| aardwolf | landscape_cat | hillslope | 0.45 | 0.17 | 0.12 | 0.77 |
| aardwolf | rugged_cat | low | 0.28 | 0.14 | 0.01 | 0.55 |
| aardwolf | aspect_cat | north | 0.49 | 0.08 | 0.34 | 0.64 |
| aardwolf | aspect_cat | south | 0.58 | 0.08 | 0.43 | 0.73 |
| aardwolf | aspect_cat | west | 0.54 | 0.08 | 0.38 | 0.69 |
| aardwolf | slope_cat | high | 0.17 | 0.07 | 0.02 | 0.31 |
| aardwolf | slope_cat | very high | 0.25 | 0.09 | 0.09 | 0.42 |
| African clawless otter | landscape_cat | alluvial fan | 0.17 | 0.06 | 0.05 | 0.29 |
| African clawless otter | landscape_cat | valley | 0.89 | 0.05 | 0.79 | 0.99 |
| African clawless otter | landscape_cat | floodplain | 0.14 | 0.07 | 0.01 | 0.27 |
| African clawless otter | landscape_cat | hillslope | 0.45 | 0.08 | 0.29 | 0.62 |
| African clawless otter | landscape_cat | high plateau | 0.23 | 0.07 | 0.09 | 0.36 |
| African clawless otter | rugged_cat | medium | 0.29 | 0.07 | 0.15 | 0.43 |
| African clawless otter | rugged_cat | high | 0.44 | 0.08 | 0.29 | 0.6 |
| African clawless otter | solar_cat | low | 0.09 | 0.05 | 0 | 0.19 |
| African clawless otter | solar_cat | medium high | 0.34 | 0.07 | 0.21 | 0.48 |
| African clawless otter | solar_cat | high | 0.11 | 0.05 | 0.02 | 0.2 |
| African clawless otter | aspect_cat | east | 0.19 | 0.09 | 0.02 | 0.36 |
| African clawless otter | aspect_cat | south | 0.25 | 0.07 | 0.12 | 0.38 |
| African wild cat | landscape_cat | floodplain | 0.16 | 0.08 | 0 | 0.32 |
| African wild cat | landscape_cat | high plateau | 0.72 | 0.2 | 0.33 | 1.11 |
| African wild cat | rugged_cat | medium | 0.55 | 0.08 | 0.39 | 0.71 |
| African wild cat | solar_cat | medium low | 0.54 | 0.09 | 0.37 | 0.72 |
| African wild cat | solar_cat | medium high | 0.52 | 0.08 | 0.36 | 0.68 |
| African wild cat | aspect_cat | east | 0.27 | 0.11 | 0.05 | 0.5 |
| African wild cat | aspect_cat | north | 0.26 | 0.11 | 0.04 | 0.49 |
| African wild cat | aspect_cat | south | 0.41 | 0.13 | 0.16 | 0.66 |
| African wild cat | aspect_cat | west | 0.48 | 0.22 | 0.05 | 0.91 |
| bat-eared fox | landscape_cat | alluvial fan | 0.44 | 0.21 | 0.04 | 0.84 |
| bat-eared fox | landscape_cat | floodplain | 0.49 | 0.24 | 0.02 | 0.95 |
| bat-eared fox | landscape_cat | high plateau | 0.92 | 0.06 | 0.8 | 1.03 |
| bat-eared fox | rugged_cat | high | 0.33 | 0.13 | 0.08 | 0.59 |
| bat-eared fox | solar_cat | medium high | 0.48 | 0.16 | 0.17 | 0.79 |
| bat-eared fox | aspect_cat | east | 0.48 | 0.21 | 0.06 | 0.89 |
| bat-eared fox | aspect_cat | south | 0.25 | 0.08 | 0.1 | 0.4 |
| bat-eared fox | aspect_cat | west | 0.46 | 0.2 | 0.06 | 0.85 |
| black-backed jackal | landscape_cat | alluvial fan | 0.55 | 0.22 | 0.11 | 0.98 |
| black-backed jackal | landscape_cat | floodplain | 0.9 | 0.06 | 0.79 | 1.01 |
| black-backed jackal | landscape_cat | high plateau | 0.39 | 0.09 | 0.21 | 0.56 |
| black-backed jackal | rugged_cat | low | 0.45 | 0.22 | 0.03 | 0.87 |
| black-backed jackal | rugged_cat | medium | 0.19 | 0.04 | 0.1 | 0.27 |
| black-backed jackal | rugged_cat | high | 0.85 | 0.04 | 0.77 | 0.92 |
| black-backed jackal | solar_cat | low | 0.09 | 0.04 | 0.01 | 0.18 |
| black-backed jackal | solar_cat | medium high | 0.96 | 0.03 | 0.9 | 1.02 |
| black-backed jackal | solar_cat | high | 0.18 | 0.06 | 0.06 | 0.29 |
| black-backed jackal | aspect_cat | south | 0.86 | 0.05 | 0.75 | 0.96 |
| black-backed jackal | slope_cat | high | 0.91 | 0.05 | 0.82 | 1.01 |
| bontebok | landscape_cat | valley | 0.34 | 0.1 | 0.15 | 0.53 |
| bontebok | rugged_cat | high | 0.36 | 0.15 | 0.07 | 0.65 |
| bontebok | solar_cat | low | 0.53 | 0.24 | 0.06 | 0.99 |
| bontebok | solar_cat | medium high | 0.28 | 0.11 | 0.07 | 0.5 |
| bontebok | solar_cat | high | 0.9 | 0.07 | 0.76 | 1.03 |
| bontebok | aspect_cat | south | 0.17 | 0.06 | 0.05 | 0.29 |
| bontebok | aspect_cat | west | 0.88 | 0.05 | 0.78 | 0.98 |
| bushbuck | landscape_cat | alluvial fan | 0.54 | 0.1 | 0.35 | 0.74 |
| bushbuck | landscape_cat | floodplain | 0.75 | 0.09 | 0.58 | 0.92 |
| bushbuck | rugged_cat | low | 0.34 | 0.13 | 0.08 | 0.6 |
| bushbuck | rugged_cat | medium | 0.66 | 0.13 | 0.41 | 0.91 |
| bushbuck | aspect_cat | south | 0.34 | 0.08 | 0.19 | 0.49 |
| bushpig | landscape_cat | valley | 0.91 | 0.05 | 0.81 | 1.01 |
| bushpig | landscape_cat | high plateau | 0.32 | 0.08 | 0.16 | 0.48 |
| bushpig | rugged_cat | high | 0.25 | 0.12 | 0.02 | 0.48 |
| bushpig | solar_cat | medium high | 0.25 | 0.11 | 0.03 | 0.48 |
| bushpig | aspect_cat | east | 0.15 | 0.06 | 0.04 | 0.26 |
| bushpig | aspect_cat | south | 0.49 | 0.07 | 0.34 | 0.63 |
| Cape buffalo | landscape_cat | valley | 0.41 | 0.11 | 0.2 | 0.62 |
| Cape buffalo | solar_cat | low | 0.37 | 0.13 | 0.12 | 0.61 |
| Cape buffalo | solar_cat | medium high | 0.27 | 0.1 | 0.07 | 0.46 |
| Cape buffalo | aspect_cat | east | 0.18 | 0.08 | 0.03 | 0.33 |
| Cape buffalo | aspect_cat | north | 0.57 | 0.08 | 0.42 | 0.72 |
| Cape grey mongoose | landscape_cat | alluvial fan | 0.34 | 0.09 | 0.17 | 0.51 |
| Cape grey mongoose | landscape_cat | valley | 0.54 | 0.26 | 0.02 | 1.05 |
| Cape grey mongoose | landscape_cat | floodplain | 0.4 | 0.09 | 0.22 | 0.57 |
| Cape grey mongoose | landscape_cat | high plateau | 0.18 | 0.07 | 0.05 | 0.32 |
| Cape grey mongoose | rugged_cat | low | 0.94 | 0.03 | 0.89 | 0.99 |
| Cape grey mongoose | rugged_cat | medium | 0.07 | 0.03 | 0.02 | 0.13 |
| Cape grey mongoose | rugged_cat | high | 0.05 | 0.02 | 0 | 0.09 |
| Cape grey mongoose | solar_cat | medium low | 0.14 | 0.06 | 0.03 | 0.25 |
| Cape grey mongoose | solar_cat | low | 0.64 | 0.22 | 0.21 | 1.07 |
| Cape grey mongoose | aspect_cat | east | 0.5 | 0.08 | 0.35 | 0.66 |
| Cape grey mongoose | aspect_cat | west | 0.57 | 0.22 | 0.14 | 1.01 |
| Cape grysbok | landscape_cat | alluvial fan | 0.93 | 0.05 | 0.84 | 1.02 |
| Cape grysbok | landscape_cat | valley | 0.14 | 0.06 | 0.02 | 0.27 |
| Cape grysbok | landscape_cat | hillslope | 0.38 | 0.09 | 0.21 | 0.56 |
| Cape grysbok | landscape_cat | high plateau | 0.65 | 0.09 | 0.48 | 0.82 |
| Cape grysbok | rugged_cat | low | 0.28 | 0.09 | 0.1 | 0.45 |
| Cape grysbok | rugged_cat | medium | 0.1 | 0.04 | 0.02 | 0.19 |
| Cape grysbok | solar_cat | medium low | 0.18 | 0.09 | 0 | 0.36 |
| Cape grysbok | aspect_cat | east | 0.25 | 0.07 | 0.12 | 0.39 |
| Cape grysbok | aspect_cat | north | 0.23 | 0.07 | 0.1 | 0.35 |
| Cape porcupine | landscape_cat | floodplain | 0.39 | 0.08 | 0.24 | 0.54 |
| Cape porcupine | landscape_cat | hillslope | 0.11 | 0.05 | 0.01 | 0.21 |
| Cape porcupine | landscape_cat | high plateau | 0.2 | 0.07 | 0.07 | 0.32 |
| Cape porcupine | rugged_cat | medium | 0.19 | 0.06 | 0.06 | 0.31 |
| Cape porcupine | rugged_cat | high | 0.42 | 0.2 | 0.03 | 0.82 |
| Cape porcupine | solar_cat | medium low | 0.89 | 0.05 | 0.79 | 0.99 |
| Cape porcupine | solar_cat | high | 0.53 | 0.09 | 0.36 | 0.71 |
| Cape porcupine | aspect_cat | east | 0.93 | 0.06 | 0.81 | 1.06 |
| Cape porcupine | aspect_cat | west | 0.2 | 0.1 | 0 | 0.41 |
| Cape porcupine | slope_cat | medium | 0.9 | 0.09 | 0.71 | 1.08 |
| caracal | landscape_cat | alluvial fan | 0.43 | 0.09 | 0.26 | 0.61 |
| caracal | landscape_cat | valley | 0.6 | 0.08 | 0.43 | 0.77 |
| caracal | rugged_cat | high | 0.67 | 0.18 | 0.31 | 1.03 |
| caracal | solar_cat | medium low | 0.37 | 0.11 | 0.15 | 0.58 |
| caracal | aspect_cat | east | 0.61 | 0.07 | 0.47 | 0.76 |
| caracal | slope_cat | very high | 0.09 | 0.03 | 0.02 | 0.16 |
| chacma baboon | landscape_cat | valley | 0.33 | 0.1 | 0.14 | 0.52 |
| chacma baboon | landscape_cat | high plateau | 0.34 | 0.09 | 0.15 | 0.52 |
| chacma baboon | rugged_cat | medium | 0.26 | 0.13 | 0.01 | 0.52 |
| chacma baboon | solar_cat | medium low | 0.62 | 0.22 | 0.19 | 1.05 |
| chacma baboon | solar_cat | high | 0.21 | 0.09 | 0.03 | 0.38 |
| chacma baboon | aspect_cat | east | 0.3 | 0.13 | 0.05 | 0.55 |
| chacma baboon | aspect_cat | west | 0.19 | 0.06 | 0.07 | 0.32 |
| common duiker | landscape_cat | alluvial fan | 0.79 | 0.08 | 0.65 | 0.94 |
| common duiker | landscape_cat | hillslope | 0.56 | 0.26 | 0.05 | 1.07 |
| common duiker | rugged_cat | low | 0.49 | 0.21 | 0.08 | 0.9 |
| common duiker | rugged_cat | high | 0.1 | 0.04 | 0.03 | 0.17 |
| common duiker | solar_cat | medium high | 0.44 | 0.21 | 0.04 | 0.84 |
| common duiker | aspect_cat | north | 0.2 | 0.06 | 0.08 | 0.32 |
| eland | landscape_cat | valley | 0.66 | 0.2 | 0.28 | 1.05 |
| eland | landscape_cat | hillslope | 0.16 | 0.07 | 0.03 | 0.29 |
| eland | landscape_cat | high plateau | 0.73 | 0.17 | 0.39 | 1.07 |
| eland | solar_cat | medium low | 0.94 | 0.04 | 0.87 | 1.02 |
| eland | solar_cat | high | 0.42 | 0.08 | 0.26 | 0.57 |
| eland | aspect_cat | east | 0.92 | 0.04 | 0.84 | 1 |
| eland | aspect_cat | north | 0.15 | 0.06 | 0.04 | 0.26 |
| eland | aspect_cat | west | 0.27 | 0.07 | 0.14 | 0.41 |
| eland | slope_cat | medium | 0.91 | 0.03 | 0.85 | 0.98 |
| eland | slope_cat | low | 0.11 | 0.04 | 0.04 | 0.19 |
| eland | slope_cat | high | 0.06 | 0.03 | 0 | 0.12 |
| eland | slope_cat | very high | 0.4 | 0.06 | 0.29 | 0.51 |
| gemsbok | landscape_cat | floodplain | 0.22 | 0.07 | 0.08 | 0.36 |
| gemsbok | landscape_cat | hillslope | 0.59 | 0.09 | 0.42 | 0.76 |
| gemsbok | landscape_cat | high plateau | 0.43 | 0.11 | 0.21 | 0.64 |
| gemsbok | rugged_cat | low | 0.65 | 0.16 | 0.34 | 0.96 |
| gemsbok | rugged_cat | high | 0.51 | 0.14 | 0.23 | 0.78 |
| gemsbok | solar_cat | medium low | 0.56 | 0.08 | 0.4 | 0.72 |
| gemsbok | solar_cat | low | 0.67 | 0.08 | 0.52 | 0.82 |
| gemsbok | aspect_cat | east | 0.4 | 0.08 | 0.25 | 0.55 |
| gemsbok | aspect_cat | north | 0.38 | 0.08 | 0.22 | 0.53 |
| gemsbok | aspect_cat | south | 0.47 | 0.19 | 0.1 | 0.83 |
| gemsbok | slope_cat | medium | 0.56 | 0.06 | 0.44 | 0.67 |
| gemsbok | slope_cat | low | 0.42 | 0.06 | 0.3 | 0.54 |
| gemsbok | slope_cat | high | 0.44 | 0.15 | 0.15 | 0.73 |
| gemsbok | slope_cat | very high | 0.1 | 0.04 | 0.02 | 0.19 |
| greater kudu | landscape_cat | alluvial fan | 0.56 | 0.26 | 0.06 | 1.07 |
| greater kudu | landscape_cat | hillslope | 0.59 | 0.09 | 0.4 | 0.77 |
| greater kudu | rugged_cat | low | 0.08 | 0.03 | 0.02 | 0.15 |
| greater kudu | solar_cat | medium low | 0.21 | 0.08 | 0.06 | 0.36 |
| greater kudu | solar_cat | high | 0.22 | 0.06 | 0.1 | 0.34 |
| greater kudu | aspect_cat | west | 0.25 | 0.09 | 0.07 | 0.44 |
| greater kudu | slope_cat | medium | 0.35 | 0.14 | 0.08 | 0.62 |
| grey rhebok | landscape_cat | valley | 0.38 | 0.09 | 0.21 | 0.55 |
| grey rhebok | rugged_cat | low | 0.33 | 0.05 | 0.23 | 0.42 |
| grey rhebok | rugged_cat | medium | 0.45 | 0.05 | 0.35 | 0.56 |
| grey rhebok | rugged_cat | high | 0.41 | 0.05 | 0.3 | 0.51 |
| grey rhebok | solar_cat | medium low | 0.17 | 0.06 | 0.04 | 0.29 |
| grey rhebok | solar_cat | low | 0.72 | 0.07 | 0.58 | 0.86 |
| grey rhebok | solar_cat | medium high | 0.3 | 0.08 | 0.14 | 0.46 |
| grey rhebok | solar_cat | high | 0.7 | 0.08 | 0.54 | 0.85 |
| grey rhebok | aspect_cat | east | 0.23 | 0.1 | 0.04 | 0.42 |
| grey rhebok | aspect_cat | north | 0.88 | 0.05 | 0.78 | 0.98 |
| grey rhebok | aspect_cat | west | 0.51 | 0.08 | 0.36 | 0.67 |
| honey badger | landscape_cat | alluvial fan | 0.39 | 0.09 | 0.21 | 0.56 |
| honey badger | landscape_cat | valley | 0.25 | 0.12 | 0.01 | 0.49 |
| honey badger | landscape_cat | high plateau | 0.41 | 0.09 | 0.24 | 0.59 |
| honey badger | rugged_cat | low | 0.19 | 0.04 | 0.1 | 0.27 |
| honey badger | solar_cat | medium low | 0.46 | 0.09 | 0.28 | 0.64 |
| honey badger | solar_cat | medium high | 0.27 | 0.08 | 0.11 | 0.42 |
| honey badger | aspect_cat | east | 0.34 | 0.08 | 0.19 | 0.49 |
| honey badger | aspect_cat | west | 0.11 | 0.05 | 0.01 | 0.21 |
| impala | landscape_cat | alluvial fan | 0.55 | 0.21 | 0.13 | 0.97 |
| impala | landscape_cat | floodplain | 0.18 | 0.07 | 0.04 | 0.32 |
| impala | rugged_cat | low | 0.32 | 0.05 | 0.22 | 0.41 |
| impala | rugged_cat | medium | 0.32 | 0.12 | 0.08 | 0.55 |
| impala | solar_cat | medium low | 0.34 | 0.08 | 0.18 | 0.49 |
| impala | solar_cat | medium high | 0.42 | 0.08 | 0.25 | 0.58 |
| impala | aspect_cat | east | 0.42 | 0.08 | 0.28 | 0.57 |
| impala | aspect_cat | south | 0.32 | 0.07 | 0.19 | 0.46 |
| klipspringer | landscape_cat | alluvial fan | 0.14 | 0.06 | 0.02 | 0.26 |
| klipspringer | landscape_cat | high plateau | 0.42 | 0.1 | 0.22 | 0.61 |
| klipspringer | rugged_cat | low | 0.26 | 0.09 | 0.09 | 0.44 |
| klipspringer | rugged_cat | medium | 0.54 | 0.05 | 0.44 | 0.64 |
| klipspringer | rugged_cat | high | 0.2 | 0.04 | 0.12 | 0.29 |
| klipspringer | solar_cat | low | 0.22 | 0.08 | 0.06 | 0.37 |
| klipspringer | solar_cat | medium high | 0.28 | 0.06 | 0.16 | 0.41 |
| klipspringer | aspect_cat | north | 0.19 | 0.06 | 0.07 | 0.31 |
| klipspringer | aspect_cat | south | 0.38 | 0.07 | 0.24 | 0.52 |
| klipspringer | aspect_cat | west | 0.26 | 0.07 | 0.13 | 0.4 |
| klipspringer | slope_cat | low | 0.58 | 0.2 | 0.19 | 0.97 |
| klipspringer | slope_cat | high | 0.36 | 0.08 | 0.19 | 0.52 |
| klipspringer | slope_cat | very high | 0.35 | 0.08 | 0.19 | 0.51 |
| large spotted genet | landscape_cat | alluvial fan | 0.79 | 0.07 | 0.64 | 0.94 |
| large spotted genet | landscape_cat | high plateau | 0.92 | 0.05 | 0.83 | 1.01 |
| large spotted genet | rugged_cat | low | 0.3 | 0.06 | 0.19 | 0.42 |
| large spotted genet | rugged_cat | high | 0.09 | 0.03 | 0.03 | 0.16 |
| large spotted genet | solar_cat | medium high | 0.32 | 0.07 | 0.19 | 0.46 |
| large spotted genet | aspect_cat | south | 0.14 | 0.05 | 0.04 | 0.25 |
| large spotted genet | aspect_cat | west | 0.48 | 0.22 | 0.05 | 0.91 |
| leopard | landscape_cat | alluvial fan | 0.11 | 0.05 | 0.01 | 0.22 |
| leopard | landscape_cat | floodplain | 0.3 | 0.07 | 0.16 | 0.45 |
| leopard | landscape_cat | hillslope | 0.36 | 0.08 | 0.2 | 0.52 |
| leopard | landscape_cat | high plateau | 0.25 | 0.07 | 0.11 | 0.39 |
| leopard | rugged_cat | medium | 0.36 | 0.05 | 0.26 | 0.47 |
| leopard | rugged_cat | high | 0.05 | 0.02 | 0 | 0.09 |
| leopard | solar_cat | medium low | 0.11 | 0.05 | 0.02 | 0.21 |
| leopard | solar_cat | low | 0.68 | 0.19 | 0.3 | 1.05 |
| leopard | solar_cat | medium high | 0.22 | 0.06 | 0.1 | 0.35 |
| leopard | solar_cat | high | 0.85 | 0.05 | 0.75 | 0.95 |
| leopard | aspect_cat | east | 0.1 | 0.05 | 0 | 0.2 |
| leopard | aspect_cat | south | 0.15 | 0.05 | 0.04 | 0.25 |
| leopard | aspect_cat | west | 0.71 | 0.07 | 0.57 | 0.85 |
| mountain reedbuck | landscape_cat | alluvial fan | 0.35 | 0.14 | 0.08 | 0.61 |
| mountain reedbuck | rugged_cat | low | 0.38 | 0.05 | 0.28 | 0.49 |
| mountain reedbuck | solar_cat | medium low | 0.19 | 0.09 | 0.01 | 0.38 |
| mountain reedbuck | solar_cat | low | 0.4 | 0.08 | 0.25 | 0.55 |
| mountain reedbuck | solar_cat | medium high | 0.22 | 0.06 | 0.1 | 0.34 |
| mountain reedbuck | solar_cat | high | 0.09 | 0.04 | 0.01 | 0.17 |
| mountain reedbuck | aspect_cat | east | 0.24 | 0.08 | 0.09 | 0.39 |
| mountain reedbuck | aspect_cat | north | 0.46 | 0.08 | 0.31 | 0.61 |
| mountain reedbuck | landscape_cat | valley | 0.48 | 0.08 | 0.32 | 0.65 |
| mountain reedbuck | landscape_cat | floodplain | 0.38 | 0.18 | 0.03 | 0.72 |
| mountain reedbuck | landscape_cat | hillslope | 0.12 | 0.06 | 0.01 | 0.24 |
| mountain reedbuck | landscape_cat | high plateau | 0.7 | 0.19 | 0.33 | 1.07 |
| mountain reedbuck | rugged_cat | medium | 0.87 | 0.05 | 0.76 | 0.98 |
| mountain reedbuck | rugged_cat | high | 0.11 | 0.05 | 0.01 | 0.2 |
| mountain reedbuck | solar_cat | medium high | 0.31 | 0.07 | 0.18 | 0.45 |
| mountain reedbuck | aspect_cat | south | 0.33 | 0.08 | 0.17 | 0.48 |
| polecat | landscape_cat | alluvial fan | 0.2 | 0.07 | 0.06 | 0.33 |
| polecat | landscape_cat | hillslope | 0.27 | 0.08 | 0.12 | 0.43 |
| polecat | landscape_cat | high plateau | 0.55 | 0.26 | 0.04 | 1.06 |
| polecat | rugged_cat | low | 0.32 | 0.07 | 0.17 | 0.46 |
| polecat | rugged_cat | medium | 0.43 | 0.19 | 0.06 | 0.8 |
| polecat | solar_cat | medium low | 0.44 | 0.07 | 0.3 | 0.58 |
| polecat | solar_cat | low | 0.44 | 0.21 | 0.03 | 0.85 |
| polecat | solar_cat | high | 0.15 | 0.06 | 0.02 | 0.27 |
| polecat | aspect_cat | east | 0.41 | 0.08 | 0.26 | 0.56 |
| polecat | aspect_cat | west | 0.21 | 0.1 | 0.01 | 0.42 |
| red hartebeest | landscape_cat | alluvial fan | 0.45 | 0.23 | 0.01 | 0.9 |
| red hartebeest | landscape_cat | floodplain | 0.27 | 0.1 | 0.07 | 0.48 |
| red hartebeest | landscape_cat | hillslope | 0.84 | 0.08 | 0.68 | 1 |
| red hartebeest | rugged_cat | medium | 0.38 | 0.11 | 0.17 | 0.6 |
| red hartebeest | rugged_cat | high | 0.5 | 0.25 | 0.01 | 0.99 |
| red hartebeest | solar_cat | medium low | 0.62 | 0.08 | 0.47 | 0.78 |
| red hartebeest | solar_cat | low | 0.44 | 0.08 | 0.28 | 0.6 |
| red hartebeest | solar_cat | medium high | 0.45 | 0.16 | 0.15 | 0.76 |
| red hartebeest | aspect_cat | east | 0.2 | 0.1 | 0 | 0.39 |
| red hartebeest | aspect_cat | north | 0.21 | 0.1 | 0 | 0.41 |
| red hartebeest | aspect_cat | south | 0.32 | 0.14 | 0.04 | 0.6 |
| red hartebeest | slope_cat | medium | 0.4 | 0.14 | 0.12 | 0.68 |
| red hartebeest | slope_cat | low | 0.55 | 0.24 | 0.08 | 1.01 |
| rock hyrax | landscape_cat | hillslope | 0.21 | 0.09 | 0.03 | 0.39 |
| rock hyrax | landscape_cat | high plateau | 0.69 | 0.17 | 0.35 | 1.03 |
| rock hyrax | rugged_cat | low | 0.16 | 0.08 | 0.01 | 0.32 |
| rock hyrax | solar_cat | low | 0.25 | 0.07 | 0.11 | 0.38 |
| rock hyrax | aspect_cat | north | 0.2 | 0.1 | 0.01 | 0.39 |
| scrub hare | landscape_cat | alluvial fan | 0.49 | 0.11 | 0.27 | 0.7 |
| scrub hare | landscape_cat | hillslope | 0.55 | 0.23 | 0.1 | 1 |
| scrub hare | landscape_cat | high plateau | 0.48 | 0.11 | 0.25 | 0.7 |
| scrub hare | rugged_cat | low | 0.11 | 0.05 | 0.01 | 0.2 |
| scrub hare | rugged_cat | medium | 0.11 | 0.05 | 0.01 | 0.2 |
| scrub hare | solar_cat | medium low | 0.11 | 0.05 | 0.01 | 0.21 |
| scrub hare | solar_cat | low | 0.84 | 0.06 | 0.72 | 0.95 |
| scrub hare | solar_cat | high | 0.6 | 0.09 | 0.43 | 0.77 |
| scrub hare | aspect_cat | east | 0.34 | 0.12 | 0.11 | 0.56 |
| scrub hare | aspect_cat | north | 0.8 | 0.1 | 0.6 | 0.99 |
| scrub hare | aspect_cat | west | 0.53 | 0.15 | 0.24 | 0.81 |
| Smith’s red rock hare | landscape_cat | floodplain | 0.22 | 0.09 | 0.03 | 0.4 |
| Smith’s red rock hare | rugged_cat | low | 0.81 | 0.06 | 0.69 | 0.93 |
| Smith’s red rock hare | rugged_cat | medium | 0.19 | 0.07 | 0.06 | 0.32 |
| Smith’s red rock hare | rugged_cat | high | 0.53 | 0.09 | 0.36 | 0.7 |
| Smith’s red rock hare | solar_cat | medium low | 0.5 | 0.1 | 0.31 | 0.69 |
| Smith’s red rock hare | solar_cat | low | 0.56 | 0.2 | 0.18 | 0.95 |
| Smith’s red rock hare | aspect_cat | east | 0.65 | 0.16 | 0.34 | 0.97 |
| springbok | landscape_cat | alluvial fan | 0.79 | 0.09 | 0.61 | 0.97 |
| springbok | landscape_cat | valley | 0.26 | 0.12 | 0.02 | 0.5 |
| springbok | landscape_cat | floodplain | 0.53 | 0.11 | 0.32 | 0.75 |
| springbok | landscape_cat | hillslope | 0.43 | 0.11 | 0.21 | 0.65 |
| springbok | rugged_cat | medium | 0.35 | 0.08 | 0.2 | 0.5 |
| springbok | solar_cat | medium low | 0.12 | 0.06 | 0 | 0.24 |
| springbok | solar_cat | low | 0.11 | 0.05 | 0.01 | 0.22 |
| springbok | aspect_cat | east | 0.41 | 0.12 | 0.16 | 0.65 |
| vervet monkey | landscape_cat | floodplain | 0.23 | 0.1 | 0.04 | 0.42 |
| vervet monkey | rugged_cat | low | 0.16 | 0.07 | 0.02 | 0.31 |
| vervet monkey | rugged_cat | high | 0.32 | 0.07 | 0.17 | 0.46 |
| vervet monkey | solar_cat | medium low | 0.76 | 0.16 | 0.44 | 1.08 |
| vervet monkey | solar_cat | medium high | 0.11 | 0.05 | 0.01 | 0.21 |
| vervet monkey | aspect_cat | east | 0.48 | 0.22 | 0.04 | 0.92 |

**Table:** Posterior summaries of species-specific occupancy across topographic covariates, including posterior means, standard deviations, and 95% Bayesian credible intervals (BCIs).

**Appendix S6:** Occupancy probabilities for mammal species across five landscape categories

**
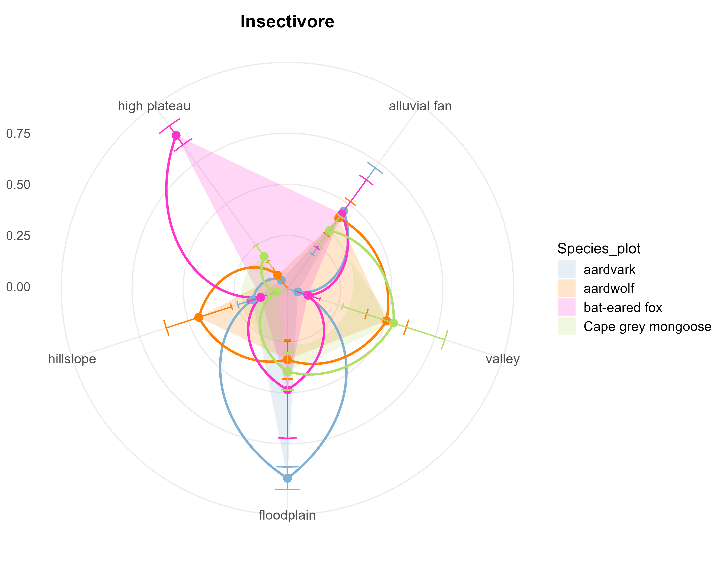
**

**
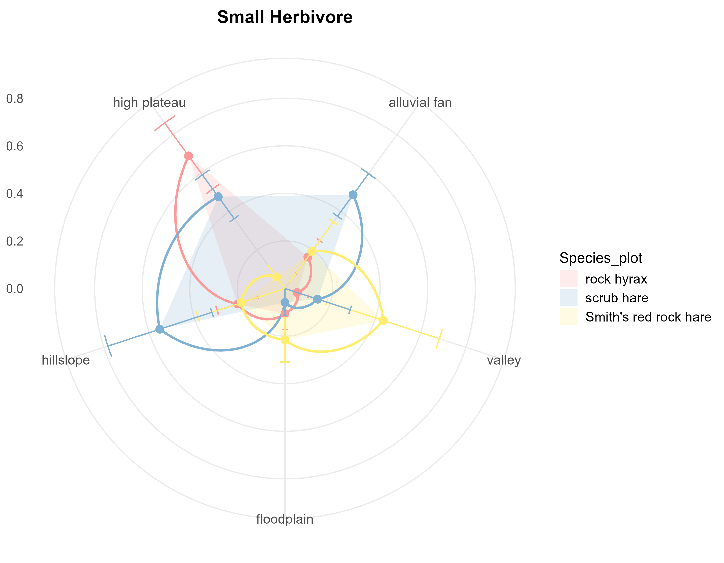
**

**
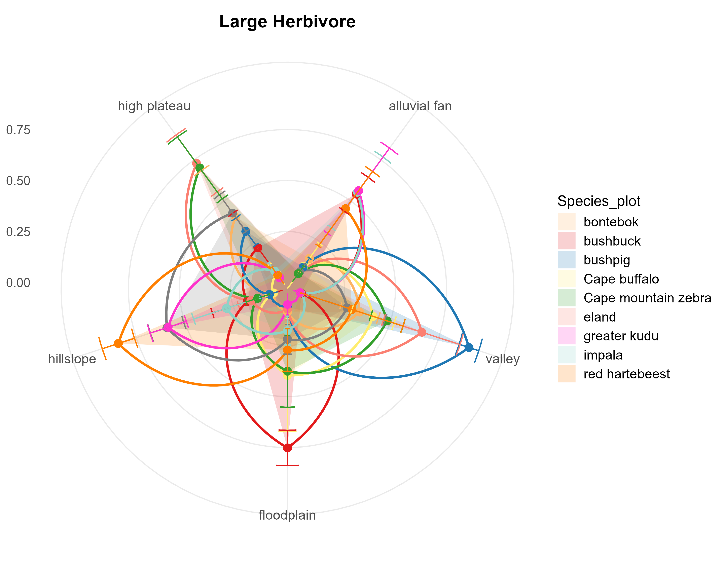

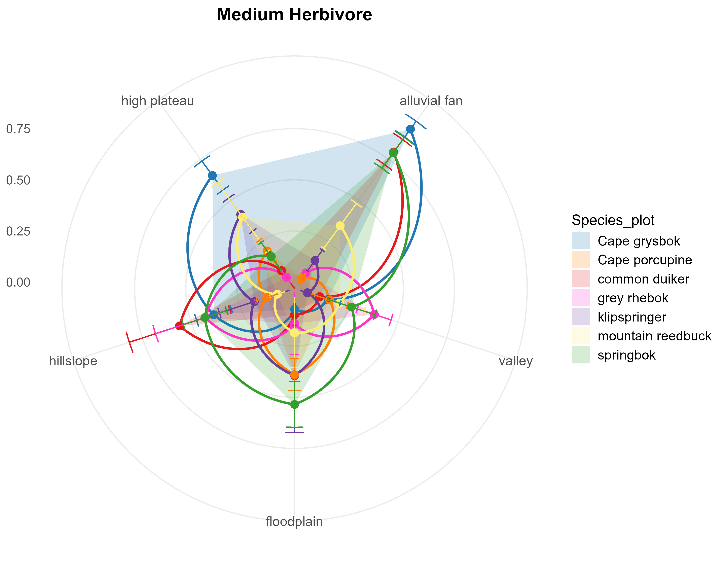
**

**
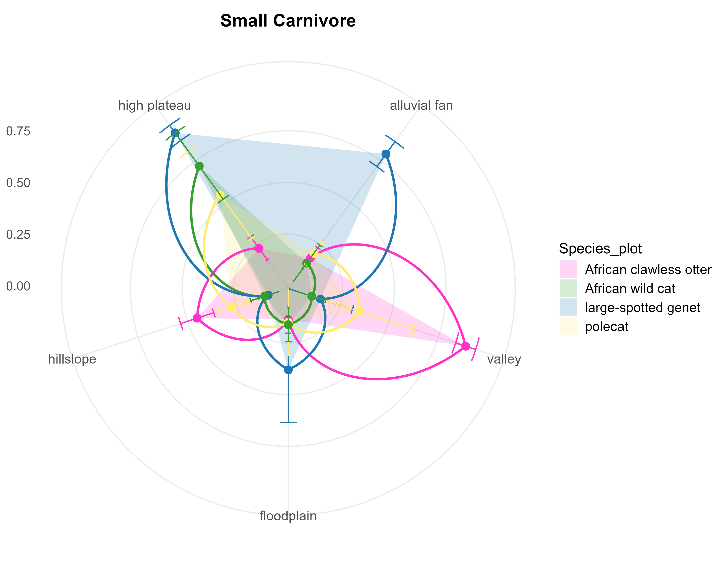
**

**
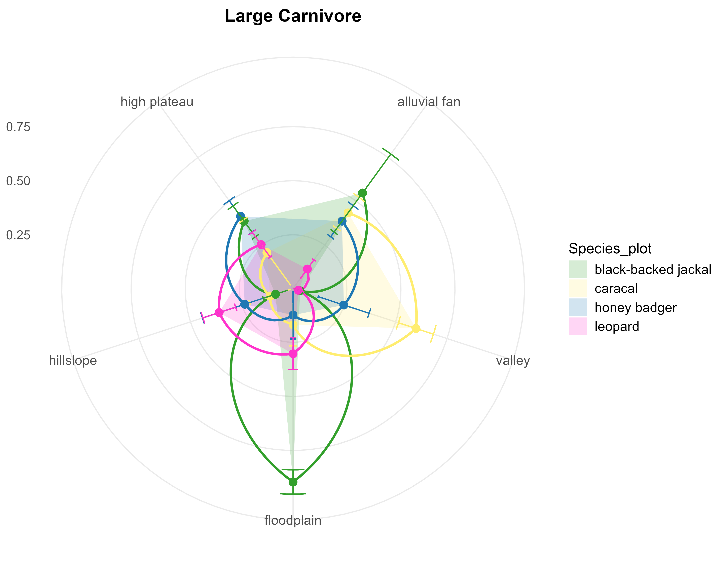
**

**
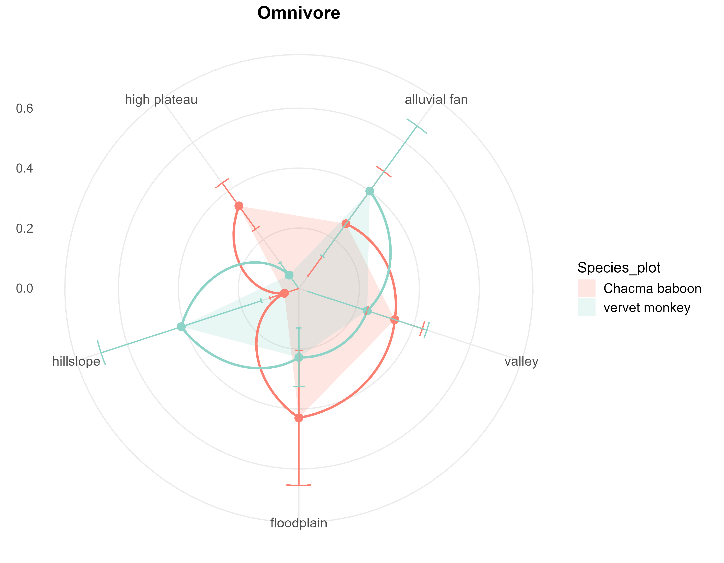
**

**Fig.** Occupancy probabilities for mammal species across five landscape categories (alluvial fan, valley, floodplain, hillslope, and high plateau), grouped by trophic level (see Appendix S4). Polygons represent mean occupancy probabilities. Species are distinguished by unique colours, and radial axes scale probabilities from 0 to 1. Error bars represent standard deviations (SD), reflecting variation around the mean.

## **Appendix S7:** Detailed discussion on species-specific habitat preferences

## This appendix presents a detailed discussion of the significant topographic categories associated with each species in relation to individual topographic features. Where possible, these findings are interpreted alongside existing literature on species’ responses to topographic complexity. For some species, extensive published information allowed for more in-depth comparisons, while for others, available data are limited, resulting in briefer descriptions. This uneven coverage underscores both the variation in species-level responses to topography and the broader gap in the literature, where research has largely emphasized community-level patterns while overlooking the fine-scale, species-specific relationships that can provide critical ecological insights.

### **S7.1 Large herbivores**

Greater kudu (*Tragelaphus strepsiceros*), prefer hillslopes and areas with low slopes, low ruggedness, south aspect, and high solar gain. Habitat use by greater kudu is primarily influenced by forage availability, with occasional nocturnal visits to plantations (Dorgeloh, Jewitt, and Slingsby 2001; Butler, 2017). Their habitat selection varies between day and night, likely reflecting shifts in activity patterns (Makhado et al. 2016). During the coldest periods, greater kudu utilise hills to avoid extreme cold, particularly at night when temperatures drop below freezing. Seasonal changes in habitat use further highlight their adaptive behaviour in response to resource availability and environmental conditions (Butler, 2017). Greater kudu are known to prefer dense cover for shelter (Fabricius, and Mentis, 1992), which explains their occurrence on south-facing slopes. Their use of slopes exposed to high solar radiation may align with their nocturnal foraging behaviour. Additionally, ruggedness influences their habitat use, with occupancy decreasing as ruggedness increases during the day but either remaining stable or increasing at night (Davies et al. 2016). These patterns underscore the greater kudu's adaptability and reliance on topographic features to meet their ecological needs.

Bontebok (*Damaliscus pygargus*) preferred high plateaus, with low radiation, a north aspect, low slopes, and medium ruggedness. These findings align with the known habitat preferences of *Damaliscus* species, which are well adapted to mountain plateaus but tend to avoid steep slopes and rocky surfaces, favouring open, flat areas instead (Luyt, 2005; Skinner and Chimimba, 2005).

Cape buffalo (*Syncerus caffer*) occupancy was only significant in indicating a preference for hillslopes with high ruggedness, showing their high variability in topographical habitat preferences. Seasonal movements are a key feature of Cape buffalo behaviour, with individuals shifting from depleted areas during the dry season to higher elevations such as plateaus. They utilise uplands and midslopes where temporary resources like water and new green grass become available, while bottomlands are used more moderately (Owen-Smith and Cross, 2004; Cornélis et al. 2011; Macandza, Snider et al. 2024).

Bushbuck (*Tragelaphus sylvaticus*) demonstrated a strong preference for high plateaus, followed by alluvial fans, with a weaker association with east-facing aspects. Primarily nocturnal, bushbuck typically occupy easily traversable terrain (Atkins et al. 2019; Ehlers Smith et al. 2020). However, wide confidence intervals for their use of more rugged areas have been recorded, suggesting they may exploit such terrains if other favourable elements, such as forage or cover, are present (Jatani, Datiko, and Worku, 2019). Additionally, bushbuck may resort to higher altitudes to reduce competition with other species (Reece et al. 2023), while also showing strong utilisation of low-lying areas where essential resources are more readily available (Jatani, Datiko, and Worku, 2019; Snider et al. 2024).

Bushpig (*Potamochoerus larvatus*) showed a preference for hillslopes with a south-facing aspect, medium-high solar gain, and low ruggedness. Bushpigs demonstrate a high degree of habitat adaptability, exploiting a wide range of conditions and topographies, with the primary driver of their distribution being access to optimal forage (Seydack, 1990). In other studies, elevation has shown a weak but nonsignificant positive association with bushpig occupancy and that bushpigs can occur at higher elevations, often restricted to mountain slopes (Leslie and Huffman, 2015; Gorczynski et al. 2023), while they are also known to utilize agricultural lands opportunistically at lower elevations (Snider et al. 2024).

Eland (*Taurotragus oryx*) demonstrated a clear preference for medium slopes, east-facing aspects, high plateaus, and areas with medium-low solar gain. In contrast, ruggedness had a weaker influence on occupancy and showed no significant effects. In other mountainous regions, such as the Drakensberg, eland are known to migrate seasonally, moving from higher elevations during summer to lower-altitude areas in winter, while avoiding steep cliffs and highly rugged terrain (Patel et al. 2019; Herrik et al. 2023). Eland are highly nomadic and adapt their foraging strategies by shifting from grazing to browsing during the dry season, when the nutritional quality of grasses declines (Buys and Dott, 1991; Kasiringua, Procheş, and Kopij, 2019). However, increasing human settlement and agricultural expansion in lowland areas appear to be limiting eland use of these habitats. Although eland historically utilised these zones more extensively, they now tend to avoid areas with human activity (Patel et al. 2019; Woodgate et al. 2023). Nevertheless, they may still access livestock grazing areas when these are temporarily free of livestock (Herrik et al. 2023).

Gemsbok (*Oryx gazella*) showed a preference for south-facing hillslopes with medium gradients, low ruggedness, and low solar gain. These habitat characteristics are consistent with observations from other regions, where gemsbok are known to favour flat to gently undulating terrain while avoiding steep, mountainous, or rocky areas (Saiz et al. 1975; Hoenes and Bender, 2010; Rocha, Bennett, and Monterroso, 2022). They also tend to avoid areas associated with human disturbance, further reinforcing their selection for more accessible and less rugged environments (Woodgate et al. 2023).

Impala (*Aepyceros melampus*) exhibited a preference for floodplains, characterised by medium-low solar gain, medium slope, an east-facing aspect, and high ruggedness. Previous studies have shown that impala tend to favour footslopes and flat to moderately sloped landscapes, while generally avoiding areas of high elevation (Grant and Scholes, 2006; Mahakata and Mapaure, 2022; Ang'ila et al. 2023; Reece et al. 2023).

Cape mountain zebra (*Equus zebra*) displayed a slight preference for hillslopes and medium slopes, while other topographical variables did not significantly influence their occupancy. The species' association with mountainous habitats, as suggested by its name, has been argued to be misleading, with insufficient historical evidence to support this being their primary habitat (Owen-Smith and Festa-Bianchet, 2003; Weel et al. 2015). Contradictory evidence suggests that Cape mountain zebras historically preferred open grasslands, but climatic and land-use changes forced them into mountainous areas (Faith, 2012). Studies support a broader habitat use by mountain zebras, showing frequent utilisation of artificial watering points in low-lying areas (Weel et al. 2015; Olivier, 2019), with low ruggedness supporting higher zebra densities (Rocha, Bennett, and Monterroso, 2022). Additionally, they have also been observed grazing on high, flat grassy plateaus (Grobler, 1983), reflecting their adaptability in habitat selection. This wide range in habitat use suggests that mountain zebras adjust their preferences to access high-quality resources throughout the year (Winkler and Owen-Smith, 1995).

Red hartebeest (*Alcelaphus buselaphus*) exhibited the highest occupancy in high plateaus, with a preference for areas characterized by medium-high solar gain, high slopes, and high ruggedness. Hartebeest abundance has been shown to decrease with increasing ruggedness during the day but not at night, suggesting a temporal component to their habitat use (Davies et al. 2016). These findings align with observations from other studies, which frequently report hartebeest utilizing high plateaus for grazing (Grobler, 1983; Ang'ila et al. 2023).

### **S7.2 Medium herbivores**

Common duiker (*Sylvicapra grimmia*) showed a preference for high plateaus, characterized by high solar gain, medium ruggedness, high slopes, and a south-facing aspect. In other studies, duikers were more commonly found in easily traversable terrain, avoiding rocky areas that could impede escape potential (Abu Baker and Brown, 2014; Reece et al. 2023). However, wide confidence intervals associated with their use of rugged terrain suggest they may occupy such areas if they offer other critical resources, such as cover or forage. Conversely, duikers have also been found to be most abundant at higher elevations and in relatively level areas well dissected by drainage lines (King, 1975; Snider et al. 2024), highlighting their adaptability to varied environmental conditions.

Grey rhebok (*Pelea capreolus*) exhibited a preference for valleys, medium ruggedness, low solar gain, west-facing aspects, and low slopes. While they are often associated with mountainous habitats featuring steep slopes and high altitudes, they are also known to utilise the full altitudinal range (Taylor, Skinner, and Krecek, 2007). Grey rhebok elsewhere have been found to predominantly occupy the Subalpine Belt, which includes the lower reaches of escarpment slopes, favouring areas with either east- or west-facing aspects (Rowe-Rowe, 1983).

Mountain reedbuck (*Redunca fulvorufula*) demonstrated a preference for valleys, characterised by low solar gain, low slopes, a north-facing aspect, and high ruggedness. While commonly found on mountain slopes (Grobler, 1983), they also favour steep areas with ample hiding places, such as boulders (Taylor and Skinner, 2006). Findings from this study align with observations that mountain reedbuck prefer lower altitudes, warmer north-facing aspects, and habitats within mountain valley bottoms and valley sides (Rowe-Rowe, 1983). These results suggest that mountain reedbuck use steep slopes in conjunction with lower slopes associated with mountain valleys, highlighting their adaptability to varied topographic features within these landscapes.

Cape grysbok (*Raphicerus melanotis*) exhibited a preference for floodplains, characterised by low slopes, a north-facing aspect, low solar gain, and high ruggedness. While near-endemic to the Cape Floristic Region (Kerley et al. 2003), Cape grysbok are found across a wide range of topographical habitats throughout their distribution and can occur in relatively small patches of indigenous vegetation surrounded by cultivated lands (Schnetler, Radloff, and O’Riain, 2021). There is some evidence suggesting a preference for shrubby thickets along low-gradient hills, foothills, kloofs, and broken landscapes (Novellie, Manson, and Bigalke, 1984; Skinner and Chimimba, 2005). However, published data on Cape grysbok’s habitat preferences in relation to topographic complexity remain limited, highlighting the need for further research into their spatial ecology.

Klipspringer (*Oreotragus oreotragus*) showed a strong preference for valleys, characterized by medium-high solar gain, high slopes, a south-facing aspect, and medium ruggedness. Klipspringer have been found to favour high-altitude areas with southwest-facing slopes ranging from 15 to 48 degrees, beyond which slopes become too steep (Smith, 2015). They prefer habitats with rocky outcrops and steep gorge sides, while using lower-lying areas for foraging (Dunbar and Dunbar, 1974: Norton, 1980; Tilson, 1980). These findings are consistent with the suggestion that, similar to mountain reedbuck, klipspringer utilize steep slopes in conjunction with adjacent lower slopes in mountain valleys, balancing their need for refuge, foraging opportunities, and predator avoidance.

Cape porcupine (*Hystrix africaeaustralis*) exhibited a slight preference for high plateaus, characterized by a south-facing aspect, high slopes, medium-high solar gain, and high ruggedness. Cape porcupines are nocturnal and display wide ecological tolerance, inhabiting forests, woodlands, savannas, grasslands, semi-arid areas, and deserts (van Aarde, 1998). They have been recorded at elevations ranging from sea level to 2,000 m (Snider et al. 2024; Skinner and Chimimba, 2005; Kingdon, 1988). Their adaptability extends to agricultural lands and urban areas, suggesting that forage availability in various land-use types is a primary driver of their range use (van Aarde, 1998; Ngcobo, Wilson, and Downs, 2019). Additionally, porcupines have been observed selecting rock outcrop formations, possibly for shelter (Viviano et al. 2020), which may explain their preference for areas with high ruggedness and steep slopes observed in this study.

Springbok (*Antidorcas marsupialis*) primarily occupied floodplains, with an east-facing aspect and medium ruggedness. Slope and solar gain did not have a significant effect on their occupancy. Springbok generally avoid mountains, rocky hills, woodlands, and other areas where vegetation restricts movement and visibility (Bigalke, 1972; Skinner and Chimimba, 2005). They tend to use foot slopes and valley bottoms during the day but may move to gentle slopes at night (Reid, 2005). While typically associated with open landscapes, they also showed some preference for relatively rugged terrain, which may provide additional foraging or shelter opportunities (Rocha, Bennett, and Monterroso, 2022).

### **S7.3 Carnivores**

Leopards (*Panthera pardus*) displayed a strong preference for hillslopes, with high solar gain, very steep slopes, and north-facing aspects, while ruggedness did not significantly influence their occupancy. As generalist carnivores, leopards are dominant predators across much of their range, occupying a wide variety of habitats, including mixed mosaics of natural and agriculture areas (Minnie, Boshoff, and Kerley, 2015; Devens et al. 2018). They are particularly abundant in mountain habitats and across elevational zones, reflecting their adaptability to diverse environmental conditions (Müller et al. 2022; McKaughan et al. 2024; Snider et al. 2024). Elevation and ruggedness have been positively associated with leopard density; however, extreme elevations and excessive ruggedness correspond with lower densities, likely due to reduced prey availability or habitat accessibility (Hinde et al. 2023). Leopards demonstrate a preference for relatively dry, rugged terrain typical of mountainous areas, which may provide refugia from human persecution and lower levels of human activity. Rugged environments also reduce direct competition for space, offering advantages over less rugged terrain (Gavashelishvili and Lukarevskiy, 2008; Swanepoel et al. 2013; Mann, O'Riain, and Parker, 2020). These findings highlight the leopard’s ecological flexibility and ability to exploit challenging terrains, underscoring the importance of conserving rugged mountainous habitats to ensure their continued survival.

Caracal (*Caracal caracal*) demonstrated mixed habitat preferences, with slightly higher but not significant occupancy in floodplains and valleys. Stronger preferences were observed for areas with medium-high solar gain, south-facing aspects, high slopes, and low ruggedness. Despite this, caracals are also known to favour rugged terrains, likely due to their suitability for providing cover, hunting opportunities, shelter, and access to prey species (Singh et al. 2014; Teichman et al. 2023). In most regions, caracals tend to avoid high elevations above 1,200 m, being more frequently found in lowland habitats and the lower thirds of slopes (Nowell and Jackson, 1996; Avenant and Nell, 1998; Ramesh, Kalle, and Downs, 2017). Conversely, in some areas, caracals have been recorded at elevations exceeding 2,500 m (Yalden, Largen, and Kock, 1980). Caracals are known to utilise modified habitats over natural ones, but their habitat use varies significantly between individuals, highlighting their adaptability and opportunistic behaviour (Ramesh, Kalle, and Downs, 2017; Teichman et al. 2023).

Honey badger (*Mellivora capensis*) exhibited a strong preference for valleys, with less pronounced influences from low ruggedness and an east-facing aspect. Known for their adaptability, honey badgers utilise a wide range of habitats, reflecting their broad niche selectivity (Allen, Peterson and Krofel, 2018). Elevation has shown mixed effects on honey badger occurrence, contributing both positively and negatively depending on the study (Chatterjee, Nigam, and Habib, 2020; Snider et al. 2024), while ruggedness appears to play a minor role in shaping their habitat use (Sharifi, Malekian, and Shahnaseri, 2020). Despite their ecological flexibility, detailed preferences for specific habitats and the influence of habitat characteristics on local abundance remain poorly understood, warranting further research (Begg et al. 2003; Allen, Peterson and Krofel, 2018).

Black-backed jackal (*Canis mesomelas*) showed a preference for valleys and areas characterised by high slopes, high ruggedness, east-facing aspects, and medium-high solar gain. They have been observed utilising areas of high terrain ruggedness, which provide cover for stalking prey and protection against human persecution (Avenant et al. 2006; Nattrass et al. 2020; Matusal and Megaze, 2023). Despite these tendencies, black-backed jackals are highly adaptable and have been found to occupy a wide range of habitats, including mountainous regions and agricultural lands, with significant variation in habitat use between individuals (Rowe-Rowe, 1982; Humphries et al. 2016; Botha, Bruns and le Roux, 2022). While they demonstrate some preference for open areas (Nattrass et al. 2020; Webster, Pretorius and Somers, 2021), their generalist behaviour allows them to thrive across diverse landscapes by exploiting a variety of resources and adapting effectively to environmental changes (Webster, Pretorius and Somers, 2021; Coulton, 2024). Multiple studies have investigated jackal home ranges (Rowe-Rowe, 1982; Kamler et al. 2019; Humphries et al. 2016; Botha, Bruns and le Roux, 2022). However, there remains a lack of research specifically addressing their habitat preferences in relation to topographic complexity, underscoring the need for further studies in this area.

Large spotted genet (*Genetta tigrina*) showed slightly higher but not significant occupancy in valleys and floodplains. Medium slopes and high ruggedness were the only significant factors influencing their occupancy. In other studies, genets have been found to prefer low-lying areas and valleys near water, avoiding higher elevations and favouring rugged areas for the shelter they provide (Virgós and Casanovas, 1997; Costa and Santos-Reis, 2002; Ramesh and Downs, 2014). However, ruggedness has also been observed to have an ambiguous association with genet habitat use, reflecting their adaptability to a variety of environments. Genets are known to occupy urban and agricultural areas, suggesting that multiple factors, including resource availability and shelter, influence their distribution (Galantinho and Mira, 2009; Widdows, Ramesh, and Downs, 2015).

African wild cat (*Felis lybica cafra*) occupied hillslopes, with preferences for medium ruggedness, medium-low solar gain, and west-facing aspects. African wild cats are distributed throughout Africa and are known for their ability to tolerate a wide range of habitats (Skinner and Chimimba, 2005; Herbst, and Mills, 2010). Studies indicate that wild cats prefer areas with low to mid-range elevations and moderate topographic complexity, which may enhance shelter and hunting opportunities (Oliveira et al. 2018; Čonč et al. 2022). Rugged terrain can improve habitat quality by providing diverse microhabitats that support prey populations, thus benefiting wildcat survival and population stability (Čonč et al. 2022).

African clawless otter (*Aonyx capensis*) and polecat (*Ictonyx striatus*) were detected infrequently, limiting the robustness of fine-scale habitat analysis. However, where detected, African clawless otters showed a preference for hillslopes, with low ruggedness and medium slopes. Polecats, on the other hand, preferred alluvial fans with high slopes, medium ruggedness, and medium-high solar gain. African clawless otters are known to occupy a variety of freshwater and marine systems, including both natural and anthropogenic environments (Okes and O’Riain, 2017). They favour riparian vegetation and geomorphological features, often selecting habitats with rocky riverbanks and boulders that provide cover and facilitate foraging (Somers and Nel, 2004; Haring, Weier, and Linden, 2023). polecats exhibit wide distribution and habitat tolerance, including preferences for dry, open grasslands and rocky outcrops (Webster, Pretorius and Somers, 2021). As habitat generalists, they have been detected across multiple habitat types and elevational zones, reflecting their adaptability to varied landscapes (Snider et al. 2024).

### **S7.4 Omnivores**

Chacma baboon (*Papio ursinus*) exhibited a preference for hillslopes, characterized by medium-low solar gain, a south-facing aspect, and high ruggedness. Widely distributed across southern Africa, chacma baboons demonstrate significant ecological flexibility, occupying a variety of habitats and showing notable morphological variability across their range (Sithaldeen, Ackermann, and Bishop, 2015; Winder, 2015; Mukuve, 2024). Often associated with low altitudes, as well as steep slopes, and human-modified habitats, chacma baboons also adapt their range use seasonally. During dry and warm summer months, they often frequent higher elevations and expand their home ranges (Hoffman and O’Riain, 2011; Mukuve, 2024). Their large home ranges and day ranges are traversed through dense, habitual route networks, which frequently follow streams, hill ridges, and established tracks within their territory (Noser and Byrne, 2007; De Raad and Hill, 2019). The preference for rugged landscapes may be linked to foraging behaviour, as baboons overturn rocks in search of food, acting as zoogeomorphic agents. This behaviour is shaped by environmental factors such as rock size, shape, and slope, highlighting their intricate interaction with their surroundings (Maré, Landman, and Kerley, 2019). This adaptability and the multifunctional use of diverse landscapes underscore the baboon's ability to thrive across a range of topographic conditions.

Vervet monkey (*Chlorocebus pygerythrus pygerythrus*) showed a preference for floodplains, characterized by medium-low solar gain, an east-facing aspect, and high ruggedness. This combination of topographical features is often encountered in broad, dry riverbeds running through the floodplain. vervet monkeys are widespread, with habitats spanning a diverse range of ecological zones, from woodland savannas to urban environments, highlighting their exceptional adaptability across East and Southern Africa (Barrett, 2005; Skinner and Chimimba, 2005; Isbell and Jaffe, 2013). In semi-arid regions, they are commonly associated with narrow riparian woodlands, where they rely on both natural and artificial water sources to sustain their populations (Pasternak et al. 2013). This adaptability enables them to exploit a variety of landscapes, including those shaped by human activity.

### **S7.5 Insectivores**

Aardvark (*Orycteropus afer*) showed a strong preference for floodplains with a south-facing aspect, high ruggedness, high slopes, and medium-high solar radiation. Although aardvarks are sometimes observed in higher elevations (Yalden et al. 1996; Epps et al. 2021), they are generally thought to avoid rocky hills, steep slopes, high ruggedness, and areas prone to seasonal flooding (Van Aarde et al. 1992; Lindsey, 1999; Taylor and Skinner, 2003; Epps et al. 2021). The findings of this study suggest that while aardvarks prefer low-lying areas, they will also utilise hillslopes and rugged terrains, indicating some adaptability in their habitat use. This highlights their ability to exploit varying topographies when other habitat requirements, such as food availability, are met.

Aardwolf (*Proteles cristata*) demonstrated a preference for hillslopes, with a south-facing aspect and high solar gain. However, other studies have found no significant effect of slope on aardwolf occupancy (van den Bosch et al. 2023). Aardwolves are generally found in low densities and occupy a wide range of dry habitats, including semi-deserts, grasslands, savanna woodlands, and gravel plains (Skinner and Chimimba, 2005; Criado, 2020; Nieman et al. 2021). Their habitat use is heavily influenced by the presence of burrows and the availability of termites, their primary food source. These factors play a critical role in determining their distribution and range use (Williams, Anderson, and Richardson, 1997; de Vries et al. 2011). This dependence on termite abundance aligns with their ecological role as specialised insectivores in arid and semi-arid ecosystems.

Bat-eared fox (*Otocyon megalotis*) exhibited a strong preference for floodplains, with high solar gain, a northern aspect, and low slope. This species is commonly found in semi-arid and arid regions of eastern and southern Africa (Skinner and Chimimba, 2005). Bat-eared foxes thrive in environments that support their primary food sources, such as ants and termites, which are abundant in short grass habitats (Klare, Kamler, and Macdonald, 2011). These include wide sandy plains where harvester termites are prevalent, enabling foxes to efficiently locate and capture food (Schuette et al. 2013; Criado, 2020). This dependency on specific prey availability highlights the importance of maintaining open, short-grass ecosystems for their survival.

### **S7.6 Small herbivores**

Cape grey mongoose (*Herpestes pulverulentus*) showed a preference for valleys, with low ruggedness, east-facing aspects, medium-high solar gain, and medium slopes. This species utilizes a wide variety of habitats, including anthropogenically modified landscapes, but showed a preference for dry shrubland and dry riverbeds. They are also known to use rocky mountain habitats while avoiding high elevations (Crawford, Crawford and Crawford, 1983; Cavallini and Nel, 1990; Criado, 2020; Schnetler, Radloff, and O’Riain, 2021).

Rock hyrax (*Procavia capensis*) exhibited a strong preference for alluvial fans characterized by high slopes, medium-low solar gain, medium ruggedness, and an eastern aspect. These colonial herbivores primarily inhabited areas with rocky outcrops, where they den in rock crevices (Skinner and Chimimba, 2005). Rock hyraxes were the most commonly found species on mountain tops within rocky afro-alpine landscapes (Snider et al. 2024). They highly value cover, which is crucial across various spatial scales, and their safety from predation largely depends on either the density of the population or the structural complexity of the habitat (Druce et al. 2006).

Smith’s red rock hare (*Pronolagus rupestris*) had the highest occupancy in alluvial fans, with preferences for low ruggedness, east-facing aspects, low solar gain, and medium slopes. Endemic to southern Africa, rock hares typically occur in low densities at higher elevations, primarily in rocky landscapes that provide natural shelter (Skinner and Chimimba, 2005). These habitats include rocky hillsides, boulder-strewn koppies, rocky ravines, and dry riverbeds with rock formations, which offer both cover and foraging opportunities (Pringle, 1974; Happold, 2013; Nieman et al. 2021; Kovacs and Oroian, 2023).

Scrub hare (*Lepus saxatilis*) showed a preference for valleys, with low solar gain, high slopes, and a north-facing aspect. The species is widespread across the southern African subcontinent and primarily occupies scrub or savanna woodland habitats with grass cover, though it readily adapts to agriculturally developed areas (Kryger, Robinson, and Bloomer, 2004; Skinner and Chimimba, 2005). Some studies have observed preferences for steep slopes and valleys (Ang'ila et al. 2023), as well as low to medium elevations (Snider et al. 2024), highlighting the hare's adaptability to a variety of landscapes that provide shelter and foraging opportunities.

**S7.7** **References**

Abu Baker, M.A. and Brown, J.S., 2014. Foraging and habitat use of common duikers, Sylvicapra grimmia, in a heterogeneous environment within the Soutpansberg, South Africa. African Journal of Ecology, 52(3), pp.318-327. https://doi.org/10.1111/aje.12122

Allen, M.L., Peterson, B. and Krofel, M., 2018. No respect for apex carnivores: distribution and activity patterns of honey badgers in the Serengeti. Mammalian Biology, 89, pp.90-94. https://doi.org/10.1016/j.mambio.2018.01.001

Ang'ila, R.O., Kimuyu, D.M., Wambugu, G.M., Kenfack, D., Musili, P.M. and Kartzinel, T.R., 2023. Fine‐scale variation in soil and topography influences herbaceous vegetation and the distribution of large mammalian herbivores. African Journal of Ecology, 61(3), pp.706-716. https://doi.org/10.1111/aje.13166

Atkins, J.L., Long, R.A., Pansu, J., Daskin, J.H., Potter, A.B., Stalmans, M.E., Tarnita, C.E. and Pringle, R.M., 2019. Cascading impacts of large-carnivore extirpation in an African ecosystem. Science, 364(6436), pp.173-177. https://doi.org/10.1126/science.aau3561

Avenant, N.L. and Nel, J.J., 1998. Home‐range use, activity, and density of caracal in relation to prey density. African Journal of Ecology, 36(4), pp.347-359. <https://doi.org/10.1046/j.1365-2028.1998.00152.x>

Avenant, N., De Waal, H.O. and Combrinck, W., 2006. The Canis Caracal Programme: a holistic approach. *B. Daly, H. Davies-Mostert, W. Davies-Mostert, S. Evans, Y. Friedmann, N. King, T. Snow, and H. Stadler, editors*, pp.23-25.

Barrett, A.S., 2005. Foraging ecology of the vervet monkey (Chlorocebus aethiops) in mixed lowveld bushveld and sour lowveld bushveld of the Blydeberg Conservancy, Northern Province, South Africa. Doctoral dissertation, University of South Africa.

Begg, C.M., Begg, K.S., Du Toit, J.T. and Mills, M.G.L., 2003. Sexual and seasonal variation in the diet and foraging behaviour of a sexually dimorphic carnivore, the honey badger (Mellivora capensis). Journal of Zoology, 260(3), pp.301-316. https://doi.org/10.1017/S0952836903003789

Bigalke, R.C., 1972. Observations on the behaviour and feeding habits of the springbok, Antidorcas marsupialis. African Zoology, 7(1), pp.333-359.

Botha, A.E., Bruns, A.C. and le Roux, A., 2022. The spatial ecology of black-backed jackals (Canis mesomelas) in a protected mountainous grassland area. African Zoology, 57(1), pp.43-55.

Butler, V.P., 2017. Feeding ecology of the greater kudu (*Tragelaphus strepsiceros*) in the central Free State. MSc thesis. University of the Free State, Bloemfontein. South Africa.

Buys, D. and Dott, H.M., 1991. Population fluctuations and breeding of eland Taurotragus oryx in a western Transvaal nature reserve. Koedoe, 34(1), pp.31-36. https://doi.org/10.4102/koedoe.v34i1.411

Cavallini, P. and Nel, J.A.J., 1990. Ranging behaviour of the Cape grey mongoose *Galerella pulverulenta* in a coastal area. Journal of Zoology, 222(3), pp.353-362. https://doi.org/10.1111/j.1469-7998.1990.tb04037.x

Chatterjee, N., Nigam, P. and Habib, B., 2020. Population estimate, habitat-use and activity patterns of the honey badger in a dry-deciduous forest of central India. Frontiers in Ecology and Evolution, 8, p.585256. <https://doi.org/10.3389/fevo.2020.585256>

Čonč, Š., Oliveira, T., Portas, R., Černe, R., Breg Valjavec, M. and Krofel, M., 2022. Dolines and cats: remote detection of karst depressions and their application to study wild felid ecology. *Remote Sensing*, *14*(3), p.656. https://doi.org/10.3390/rs14030656

Coulton, V., 2024. *Population densities of mesocarnivores across protected and non-protected private landscapes in the Eastern Cape of South Africa* (Doctoral dissertation, Durham University).

Cornélis, D., Benhamou, S., Janeau, G., Morellet, N., Ouedraogo, M. and De Visscher, M.N., 2011. Spatiotemporal dynamics of forage and water resources shape space use of West African savanna buffaloes. Journal of Mammalogy, 92(6), pp.1287-1297. https://doi.org/10.1644/10-MAMM-A-397.1

Costa, H. and Santos-Reis, M., 2002. Use of middens by the common genet (Genetta genetta L.) and its relation with the landscape structure in Grândola Mountain (SW Portugal). Revista de Biologia (Lisboa), 20, pp.135-145.

Criado, A.A., 2020. Small-carnivores population survey in Southern Namibia. Master's Thesis, Czech University of Life Sciences Prague.

Crawford, P.D., Crawford, S.A.H. and Crawford, R.M., 1983. Some observations on Cape grey mongooses Herpestes pulverulentus in the Tsitsikamma National Parks. South African Journal of Wildlife Research, 13(2), pp.35-40.

Davies, A.B., Tambling, C.J., Kerley, G.I. and Asner, G.P., 2016. Limited spatial response to direct predation risk by African herbivores following predator reintroduction. Ecology and Evolution, 6(16), pp.5728-5748. https://doi.org/10.1002/ece3.2312

De Raad, A.L. and Hill, R.A., 2019. Topological spatial representation in wild chacma baboons (Papio ursinus). Animal Cognition, 22(3), pp.397-412. https://doi.org/10.1007/s10071-019-01253-6

De Vries, J.L., Pirk, C.W.W., Bateman, P.W., Cameron, E.Z. and Dalerum, F., 2011. Extension of the diet of an extreme foraging specialist, the aardwolf (Proteles cristata). African Zoology, 46(1), pp.194-196.

Devens, C., Tshabalala, T., McManus, J. and Smuts, B., 2018. Counting the spots: The use of a spatially explicit capture–recapture technique and GPS data to estimate leopard (Panthera pardus) density in the Eastern and Western Cape, South Africa. African Journal of Ecology, 56(4), pp.850-859. https://doi.org/10.1111/aje.12512

Dorgeloh, A.L., Jewitt, D. and Slingsby, J.A., 2021. Rates and patterns of habitat loss across South Africa's vegetation biomes. South African Journal of Science, 117(1-2), pp.1-5. https://doi.org/10.17159/sajs.2021/8182

Druce, D.J., Brown, J.S., Castley, J.G., Kerley, G.I., Kotler, B.P., Slotow, R. and Knight, M.H., 2006. Scale‐dependent foraging costs: habitat use by rock hyraxes (Procavia capensis) determined using giving‐up densities. Oikos, 115(3), pp.513-525. <https://doi.org/10.1111/j.2006.0030-1299.15179.x>

Dunbar, R.I.M. and Dunbar, E.P., 1974. Ecological relations and niche separation between sympatric terrestrial primates in Ethiopia. *Folia primatologica*, *21*(1), pp.36-60. https://doi.org/10.1159/000155595

Ehlers Smith, Y.C., Ehlers Smith, D.A., Ramesh, T. and Downs, C.T., 2020. Co‐occurrence modelling highlights conservation implications for two competing spiral‐horned antelope. Austral ecology, 45(3), pp.305-318. https://doi.org/10.1111/aec.12856

Epps, C.W., Weldy, M.J., Crowhurst, R.S. and Spaan, R.S., 2021. Estimating the distribution and habitat suitability for aardvarks (Orycteropus afer) in Kruger National Park, South Africa. African Journal of Ecology, 59(4), pp.854-865. https://doi.org/10.1111/aje.12916

Fabricius, C. and Mentis, M., 1992. Modelling the habitat relations of kudu in arid savanna. South African Journal of Science, 88(5), pp.280-284.

Faith, J.T., 2012. Palaeozoological insights into management options for a threatened mammal: southern Africa’s Cape mountain zebra (Equus zebra zebra). Diversity and Distributions, 18(5), pp.438-447. https://doi.org/10.1111/j.1472-4642.2011.00841.x

Galantinho, A. and Mira, A., 2009. The influence of human, livestock, and ecological features on the occurrence of genet (Genetta genetta): a case study on Mediterranean farmland. Ecological Research, 24, pp.671-685. https://doi.org/10.1007/s11284-008-0538-5

Gavashelishvili, A. and Lukarevskiy, V., 2008. Modelling the habitat requirements of leopard Panthera pardus in west and central Asia. Journal of Applied Ecology, 45(2), pp.579-588. <https://doi.org/10.1111/j.1365-2664.2007.01432.x>

Gorczynski, D., 2023. *The Functional Side of Diversity: Effects of Environmental Conditions and Human Disturbance on Tropical Forest Mammal Communities* (Doctoral dissertation, Rice University).

Grobler, J.H., 1983. Feeding habits of the Cape mountain zebra Equus zebra zebra Linn. 1758. Koedoe, 26(1), pp.159-168. https://doi.org/10.4102/koedoe.v26i1.596

Grant, C.C. and Scholes, M.C., 2006. The importance of nutrient hot-spots in the conservation and management of large wild mammalian herbivores in semi-arid savannas. Biological Conservation, 130(3), pp.426-437. <https://doi.org/10.1016/j.biocon.2006.01.004>

Happold, D., 2013. Order LAGOMORPHA Hares, Rock-hares, Rabbits, Pikas. In *Mammals of Africa Volume III: Rodents, Hares and Rabbits* (p. 693). Bloomsbury.

Haring, C., Weier, S. and Linden, B., 2023. Distribution and Habitat Preference of Cape Clawless otters (Aonyx capensis) and Water Mongooses (Atilax paludinosus) in the Soutpansberg, South Africa. IUCN otter Specialist Group Bulletin, 40(1), pp.26-38.

Herbst, M. and Mills, M.G.L., 2010. The feeding habits of the Southern African wildcat, a facultative trophic specialist, in the southern Kalahari (Kgalagadi Transfrontier Park, South Africa/Botswana). Journal of Zoology, 280(4), pp.403-413. https://doi.org/10.1111/j.1469-7998.2009.00679.x

Herrik, A.L., Mogensen, N., Svenning, J.C. and Buitenwerf, R., 2023. Rotational grazing with cattle‐free zones supports the coexistence of cattle and wild herbivores in African rangelands. Journal of Applied Ecology, 60(10), pp.2154-2166. https://doi.org/10.1111/1365-2664.14493

Hinde, K., Wilkinson, A., Tokota, S., Amin, R., O’Riain, M.J. and Williams, K.S., 2023. Leopard density and the ecological and anthropogenic factors influencing density in a mixed-use landscape in the Western Cape, South Africa. Plos one, 18(10), p.e0293445. https://doi.org/10.1371/journal.pone.0293445

Hoffman, T.S. and O'Riain, M.J., 2011. The spatial ecology of chacma baboons (Papio ursinus) in a human-modified environment. International Journal of Primatology, 32, pp.308-328. https://doi.org/10.1007/s10764-010-9467-6

Hoenes, B.D. and Bender, L.C., 2010. Relative habitat-and browse-use of native desert mule deer and exotic oryx in the greater San Andres Mountains, New Mexico. Human-Wildlife Interactions, 4(1), pp.12-24.

Humphries, B.D., Ramesh, T., Hill, T.R. and Downs, C.T., 2016. Habitat use and home range of black-backed jackals (Canis mesomelas) on farmlands in the Midlands of KwaZulu-Natal, South Africa. African Zoology, 51(1), pp.37-45.

Isbell, L.A. and Jaffe, K. E. (2013). Chlrocebus pygerythrus: Vervet Monkey. In T.M. Butynski, J. Kingdon, and J. Kalina (Eds.), Mammals of Africa, Volume 2: Primates. New York: Bloomsbury Press, pp. 277-283.

Jatani, D., Datiko, D. and Worku, D., 2019. Population Size and Habitat Utilization of Menelik’s Bushbuck (Tragelaphus scriptus Meneliki Neumann, 1902) in Abasheba-Demero Controlled Hunting Area, Southeastern Ethiopia. Adv Life Sci Technol, 76, p.10.7176.

Kamler, J.F., Stenkewitz, U., Gharajehdaghipour, T. and Macdonald, D.W., 2019. Social organization, home ranges, and extraterritorial forays of black‐backed jackals. The Journal of Wildlife Management, 83(8), pp.1800-1808. https://doi.org/10.1002/jwmg.21748

Kasiringua, E., Procheş, Ş. and Kopij, G., 2019. Population structure of ungulates in Waterberg National Park, Namibia. Vestnik Zoologii, 53(1), pp.31-46. DOI 10.2478/vzoo-2019-0004

Kerley, G.I., Pressey, R.L., Cowling, R.M., Boshoff, A.F. and Sims-Castley, R., 2003. Options for the conservation of large and medium-sized mammals in the Cape Floristic Region hotspot, South Africa. Biological Conservation, 112(1-2), pp.169-190. https://doi.org/10.1016/S0006-3207(02)00426-3

King, D.G., 1975. The afro-alpine grey duiker of Kilimanjaro. Journal of East African Natural History, 1975(152), pp.1-9.

Kingdon, J., 1988. East African mammals: an atlas of evolution in Africa, volume 3, Part A: Carnivores. University of Chicago Press. USA.

Klare, U., Kamler, J.F. and Macdonald, D.W., 2011. The bat-eared fox: a dietary specialist?. Mammalian Biology, 76, pp.646-650. https://doi.org/10.1016/j.mambio.2011.06.005

Kovacs, E. and Oroian, I., 2023. Genus Pronolagus Lyon 1904. Rabbit Genetics, 13(1).

Kryger, U., Robinson, T.J. and Bloomer, P., 2004. Population structure and history of southern African scrub hares, Lepus saxatilis. Journal of Zoology, 263(2), pp.121-133. https://doi.org/10.1017/S0952836904004947

Leslie Jr, D.M. and Huffman, B.A., 2015. *Potamochoerus porcus* (Artiodactyla: Suidae). Mammalian Species, 47(919), pp.15-31. https://doi.org/10.1093/mspecies/sev002

Lindsey, P.A., 1999. The feeding ecology and habitat use of the aardvark, Orycteropus afer. MSc Thesis. University of Pretoria, 124 pp.

Luyt, E.D.C., 2005. Models of bontebok (Damaliscus pygargus pygargus, Pallas 1766) habitat preferences in the bontebok National Park and sustainable stocking rates. Doctoral dissertation, Stellenbosch University. South Africa.

Macandza, V.A., Owen-Smith, N. and Cross, P.C., 2004. Forage selection by African buffalo in the late dry season in two landscapes. South African Journal of Wildlife Research, 34(2), pp.113-121.

Mahakata, I. and Mapaure, I., 2022. Effects of prescribed burning on abundance of common herbivores in Matekenya Vlei, Sengwa Wildlife Research Area (SWRA), Zimbabwe. Open Access Library Journal, 9(11), pp.1-14.

Makhado, R., Potgieter, M., Luus-Powell, W., Cooper, S., Oppong, C., Kopij, G., Mutisi, C. and Makhabu, S., 2016. Tragelaphus strepsiceros browse during the dry season in the mopani veld of Limpopo Province, South Africa. Transactions of the Royal Society of South Africa, 71(1), pp.17-21. https://doi.org/10.1080/0035919X.2015.1102174

Mann, G.K., O'Riain, M.J. and Parker, D.M., 2020. A leopard's favourite spots: Habitat preference and population density of leopards in a semi-arid biodiversity hotspot. Journal of Arid Environments, 181, p.104218. https://doi.org/10.1016/j.jaridenv.2020.104218

Maré, C., Landman, M. and Kerley, G.I., 2019. Rocking the landscape: Chacma baboons (Papio ursinus) as zoogeomorphic agents. Geomorphology, 327, pp.504-510. https://doi.org/10.1016/j.geomorph.2018.11.028

Matusal, M. and Megaze, A., 2023. Diet of black-backed jackal (*Canis mesomelas*, Schreber, 1775), impacts on livelihood and perceptions of farmers in Konasa Pulasa community conserved forest, Omo Valley of Ethiopia. BMC Zoology, 8(1), p.27. https://doi.org/10.1186/s40850-023-00186-5

McKaughan J.E.T., Stephens P.A., Lucas C., Guichard-Kruger N., Guichard-Kruger F. and Hill R.A. 2024. Leopard density and determinants of space use in a farming landscape in South Africa. Dental Science Reports, 14. https://doi.org/10.1038/s41598-024-61013-6

Minnie, L., Boshoff, A.F. and Kerley, G.I., 2015. Vegetation type influences livestock predation by leopards: implications for conservation in agro-ecosystems. African Journal of Wildlife Research, 45(2), pp.204-214. <https://doi.org/10.3957/056.045.0204>

Mukuve, B.Z., 2024. *Analysis of seasonal variation in dietary composition and behaviour of wild chacma baboons (Papio ursinus) in two habitats of different human influence around windhoek, central Namibia* (Doctoral dissertation, University of Namibia).

Müller, L., Briers-Louw, W.D., Seele, B.C., Stefanus Lochner, C. and Amin, R., 2022. Population size, density, and ranging behaviour in a key leopard population in the Western Cape, South Africa. PLoS One, 17(5), p.e0254507. https://doi.org/10.1371/journal.pone.0254507

Nattrass, N., Conradie, B., Stephens, J. and Drouilly, M., 2020. Culling recolonizing mesopredators increases livestock losses: Evidence from the South African Karoo. Ambio, 49, pp.1222-1231. https://doi.org/10.1007/s13280-019-01260-4

Nieman, W.A., Schultz, B.C., Wilkinson, A. and Leslie, A.J., 2021. Stakeholders’ perceptions of mammal occurrence and abundance on agricultural properties bordering the Boland Mountain Complex, South Africa. African Zoology, 56(2), pp.104-116.

Ngcobo, S.P., Wilson, A.L. and Downs, C.T., 2019. Habitat selection of Cape porcupines in a farmland-suburban context in KwaZulu-Natal, South Africa. *Mammalian Biology*, *98*(1), pp.111-118. https://doi.org/10.1016/j.mambio.2019.08.004

Norton, P.M., 1980. *The habitat and feeding ecology of the klipspringer Oreotragus Oreotragus (Zimmermann, 1973) in two areas of the Cape Province*. University of Pretoria (South Africa).

Noser, R. and Byrne, R.W., 2007. Travel routes and planning of visits to out-of-sight resources in wild chacma baboons, Papio ursinus. Animal Behaviour, 73(2), pp.257-266. https://doi.org/10.1016/j.anbehav.2006.04.012

Novellie, P.A., Manson, J. and Bigalke, R.E., 1984. Behavioural ecology and communication in the Cape grysbok. African Zoology, 19(1), pp.22-30.

Nowell, K. and Jackson, P., eds., 1996. Wild cats: status survey and conservation action plan (Vol. 382). Gland, Switzerland: IUCN.

Okes, N.C. and O'Riain, M.J., 2017. otter occupancy in the Cape Peninsula: Estimating the probability of river habitat use by Cape clawless otters, Aonyx capensis, across a gradient of human influence. Aquatic Conservation: Marine and Freshwater Ecosystems, 27(3), pp.706-716. https://doi.org/10.1002/aqc.2738

Oliveira, T., Urra, F., López‐Martín, J.M., Ballesteros‐Duperón, E., Barea‐Azcón, J.M., Moléon, M., Gil‐Sánchez, J.M., Alves, P.C., Díaz‐Ruíz, F., Ferreras, P. and Monterroso, P., 2018. Females know better: Sex‐biased habitat selection by the European wildcat. Ecology and Evolution, 8(18), pp.9464-9477. https://doi.org/10.1002/ece3.4442

Olivier, A.J., 2019. Ecology and habitat suitability of Cape mountain zebra (*Equus zebra zebra*) in the Western Cape, South Africa. Doctoral dissertation, Stellenbosch University. South Africa.

Owen-Smith, N., and Festa-Bianchet, M., 2003. Foraging behavior, habitat suitability, and translocation success, with special reference to large mammalian herbivores. *Animal behavior and wildlife conservation*, 93e109.

Pasternak, G., Kienzle, S., Barrett, L., Henzi, P., Brown, L.R. and Fuller, A., 2013. Population ecology of vervet monkeys in a high latitude, semi-arid riparian woodland. Koedoe: African Protected Area Conservation and Science, 55(1), pp.1-9.

Patel, T., O’Connor, T., Parrini, F. and Krüger, S., 2019. Common eland (*Tragelaphus oryx*) population trends in the uKhahlamba-Drakensberg Park and surrounds, South Africa, between 1942 and 2018. African Journal of Wildlife Research, 49(1), pp.121-136.

Pringle, J.A., 1974. The distribution of mammals in Natal. Part 1. Primates, Hyracoidea, Lagomorpha (except Lepus), Pholidota and Tubulidentata. Annals of the Natal Museum, 22, pp.173-186.

Ramesh, T., Kalle, R. and Downs, C.T., 2017. Space use in a South African agriculture landscape by the caracal (Caracal caracal). European Journal of Wildlife Research, 63(1), p.11. https://doi.org/10.1007/s10344-016-1072-3.

Reece, S.J., Tambling, C.J., Leslie, A.J. and Radloff, F.G.T., 2023. Patterns and predictors of ungulate space use across an isolated Miombo woodland reserve. Journal of Zoology, 320(2), pp.143-159. https://doi.org/10.1111/jzo.13059

Reid, C., 2005. Habitat Suitability and Behaviour of springbok (Antidorcas marsupialis) at Augrabies Falls National Park, South Africa. Doctoral dissertation, University of Port Elizabeth. South Africa.

Rocha, F., Bennett, B. and Monterroso, P., 2022. Understanding top-down and bottom-up processes in an ungulate community to define conservation priorities in a desert environment. Biodiversity and Conservation, 31(8), pp.2179-2203. https://doi.org/10.1007/s10531-022-02438-1

Rowe-Rowe, D.T., 1982. Home range and movements of black-backed jackals in an African montane region. South African Journal of Wildlife Research, 12(3), pp.79-84.

Rowe-Rowe, D.T., 1983. Habitat preferences of five Drakensberg antelopes. South African Journal of Wildlife Research, 13(1), pp.1-8.

Saiz, R.B., Decker, E., Lehner, P.N. and Welch, R.D., 1975. Ecology and behavior of the gemsbok at White Sands Missile Range, New Mexico. Prepared for the New Mexico Department of Game and Fish, Federal Aid Project W-111-R-8. USA.

Schuette, P., Wagner, A.P., Wagner, M.E. and Creel, S., 2013. Occupancy patterns and niche partitioning within a diverse carnivore community exposed to anthropogenic pressures. Biological Conservation, 158, pp.301-312. https://doi.org/10.1016/j.biocon.2012.08.008

Schnetler, A.K., Radloff, F.G. and O’Riain, M.J., 2021. Medium and large mammal conservation in the City of Cape Town: factors influencing species richness in urban nature reserves. Urban Ecosystems, 24(2), pp.215-232. <https://doi.org/10.1007/s11252-020-01027-w>

Seydack, A.H., Bigalke, R.C. and Jacobs, I., 1990. Ecology of the bushpig Potamochoerus porcus linn. 1758 in the Cape Province, South Africa.

Sharifi, H., Malekian, M. and Shahnaseri, G., 2020. Habitat selection of honey badgers: are they at the risk of an ecological trap? Hystrix, 31(2), p.131. DOI: 10.4404/hystrix-00352-2020

Singh, R., Qureshi, Q., Sankar, K., Krausman, P.R. and Goyal, S.P., 2014. Population and habitat characteristics of caracal in semi-arid landscape, western India. Journal of arid environments, 103, pp.92-95. <https://doi.org/10.1016/j.jaridenv.2014.01.004>

Sithaldeen, R., Ackermann, R.R. and Bishop, J.M., 2015. Pleistocene aridification cycles shaped the contemporary genetic architecture of Southern African baboons. *PLoS One*, *10*(5), p.e0123207. https://doi.org/10.1371/journal.pone.0123207

Skinner, J.D. and Chimimba, C.T., 2005. The Mammals of the Southern African Subregion. 3rd ed. Cambridge University Press, Cambridge, UK.

Smith, R.J., 2015. *Development of a habitat suitability model to determine the potential distribution of Klipspringer (Oreotragus Oreotragus subsp. Oreotragus) in Table Mountain National Park* (Doctoral dissertation, Cape Peninsula University of Technology).

Snider, M.H., Helgen, K.M., Young, H.S., Agwanda, B., Schuttler, S., Titcomb, G.C., Branch, D., Dommain, R. and Kays, R., 2024. Shifting mammal communities and declining species richness along an elevational gradient on Mount Kenya. Ecology and Evolution, 14(4), p.e11151. https://doi.org/10.1002/ece3.11151

Somers, M.J. and Nel, J.A., 2004. Habitat selection by the Cape clawless otter (Aonyx capensis) in rivers in the Western Cape Province, South Africa. African Journal of Ecology, 42(4), pp.298-305. https://doi.org/10.1111/j.1365-2028.2004.00526.x

Swanepoel, L.H., Lindsey, P., Somers, M.J., Van Hoven, W. and Dalerum, F., 2013. Extent and fragmentation of suitable leopard habitat in South Africa. Animal Conservation, 16(1), pp.41-50. https://doi.org/10.1111/j.1469-1795.2012.00566.x

Taylor, W.A. and Skinner, J.D., 2003. Activity patterns, home ranges and burrow use of aardvarks (*Orycteropus afer*) in the Karoo. Journal of Zoology, 261(3), pp.291-297. https://doi.org/10.1017/S0952836903004217

Taylor, W.A. and Skinner, J.D., 2006. A review of the social organisation of mountain reedbuck, *Redunca fulvorufula*, and grey rhebok, *Pelea capreolus*, in relation to their ecology. Transactions of the Royal Society of South Africa, 61(1), pp.8-10.

Taylor, W.A., Skinner, J.D. and Krecek, R.C., 2007. Home ranges of sympatric grey rhebok and mountain reedbuck in a South African highveld grassland. *African Zoology*, *42*(2), pp.145-151. https://doi.org/10.1080/15627020.2007.11407390

Teichman, K.J., Cristescu, B., Crevier, L., O'Riain, M.J. and Hodges, K.E., 2023. Movement choices of persecuted caracals on farmlands in South Africa. Rangeland Ecology and Management, 88, pp.77-84. https://doi.org/10.1016/j.rama.2023.02.004

Tilson, R.L., 1980. Klipspringer (*Oreotragus oreotragus*) social structure and predator avoidance in a desert canyon. Madoqua, 11(4), pp.303-314.

Van Aarde, R.J., Willis, C.K., Skinner, J.D. and Haupt, M.A., 1992. Range utilization by the aardvark, Orycteropus afer (Pallas, 1766) in the Karoo, South Africa. Journal of arid environments, 22(4), pp.387-394. <https://doi.org/10.1016/S0140-1963(18)30581-0>

van Aarde, R., 1998. An ecological perspective of reproduction in the Cape porcupine. Transactions of the royal society of South Africa, 53(2), pp.237-243. <https://doi.org/10.1080/00359199809520390>

van den Bosch, M., Kellner, K.F., Mkasanga, I., Mwampeta, S.B., Fyumagwa, R., Gantchoff, M.G., Patterson, B.R. and Belant, J.L., 2023. Spatial and temporal niche overlap of aardwolves and aardvarks in Serengeti National Park, Tanzania. *Ecology and Evolution*, *13*(11), p.e10718. https://doi.org/10.1002/ece3.10718

Virgós, E. and Casanovas, J.G., 1997. Habitat selection of genet Genetta genetta in the mountains of central Spain. *Acta Theriologica*, *42*(2), pp.169-177.

Viviano, A., Amori, G., Luiselli, L., Oebel, H., Bahleman, F. and Mori, E., 2020. Blessing the rains down in Africa: spatiotemporal behaviour of the crested porcupine *Hystrix cristata* (Mammalia: Rodentia) in the rainy and dry seasons, in the African savanna. Tropical Zoology, 33(4). https://doi.org/10.4081/tz.2020.80

Webster, A.B., Pretorius, M.E. and Somers, M.J., 2021. The determinants of mesocarnivore activity patterns in highveld grassland and riparian habitats. African Journal of Wildlife Research, 51(1), pp.178-192.

Weel, S., Watson, L.H., Weel, J., Venter, J.A. and Reeves, B., 2015. Cape mountain zebra in the Baviaanskloof Nature Reserve, South Africa: Resource use reveals limitations to zebra performance in a dystrophic mountainous ecosystem. African Journal of Ecology, 53(4), pp.428-438. https://doi.org/10.1111/aje.12215

Widdows, C.D., Ramesh, T. and Downs, C.T., 2015. Factors affecting the distribution of large spotted genets (*Genetta tigrina*) in an urban environment in South Africa. Urban Ecosystems, 18, pp.1401-1413. <https://doi.org/10.1007/s11252-015-0449-5>

Williams, J.B., Anderson, M.D. and Richardson, P.R.K., 1997. Seasonal differences in field metabolism, water requirements, and foraging behavior of free‐living aardwolves. *Ecology*, *78*(8), pp.2588-2602. https://doi.org/10.1890/0012-9658(1997)078[2588:SDIFMW]2.0.CO;2

Winder, I.C., 2015. The biogeography of the Papio baboons: a GIS-based analysis of range characteristics and variability. *Folia primatologica*, *85*(5), pp.292-318. https://doi.org/10.1159/000362545

Winkler, A. and Owen-Smith, N., 1995. Habitat utilisation by Cape mountain zebras in the mountain zebra National Park, South Africa. Koedoe, 38(1), pp.83-93. https://doi.org/10.4102/koedoe.v38i1.308

Woodgate, Z., Drouilly, M., Distiller, G. and O’Riain, M.J., 2023. The effect of multi-use landscapes on mammal assemblages and its implication for conservation. Land, 12(3), p.599. https://doi.org/10.3390/land12030599

Yalden, D.W., Largen, M.J. and Kock, D., 1980. Catalogue of the Mammals of Ethiopia: 4. Carnivora. Monitore Zoologico Italiano. Supplemento, 13(1), pp.169-272. https://doi.org/10.1080/00269786.1980.11758553

Yalden, D.W., Largen, M.J., Kock, D. and Hillman, J.C., 1996. Catalogue of the mammals of Ethiopia and Eritrea. 7. Revised checklist, zoogeography and conservation. *Tropical Zoology*, *9*(1), pp.73-164. https://doi.org/10.1080/03946975.1996.10539304
